# Spectrum of Movement Disorders in Hematological Malignancies: A Comprehensive Systematic Review of Clinical Phenotypes, Mechanisms, and Outcomes

**DOI:** 10.5334/tohm.1147

**Published:** 2026-03-19

**Authors:** Ravindra Kumar Garg, Amita Jain, Ritu Karoli, Shweta Pandey, Vimal Paliwal, Vinay Suresh, Sanjay Singhal

**Affiliations:** 1 Department of Neurology, Era’s Lucknow Medical College & Hospital, Era University, Lucknow, India; 2 All India Institute of Medical Sciences, Rae Bareli, Uttar Pradesh, India; 3 Department of Medicine, Dr RML Institute of Medical Sciences, Lucknow, India; 4 Department of Neurology, King George’s Medical University, Lucknow, India; 5 Department of Neurology, Sanjay Gandhi Institute of Medical Sciences, Lucknow, India; 6 Department of Psychiatry, Warne ford Hospital, University of Oxford, United Kingdom; 7 Department of Pulmonary Medicine, TS Mishra Medical College, Lucknow, India

**Keywords:** Hematologic Neoplasms, Movement Disorders, Central Nervous System Diseases, Paraneoplastic Syndromes, Myeloproliferative Disorders

## Abstract

**Background:**

Movement disorders associated with hematologic malignancies remain incompletely defined. This review synthesized published articles to clarify clinical patterns, mechanisms, and outcomes across hematologic malignancies.

**Methods:**

A PRISMA-compliant systematic search of PubMed, Embase, Scopus, and Google Scholar was conducted to identify reports describing movement disorders associated with hematologic malignancies. Eligible reports were screened according to predefined criteria, and data were extracted on demographics, hematologic diagnosis, central nervous system (CNS) involvement, movement-disorder phenotype, investigative findings, treatments administered, and neurological outcomes.

**Results:**

A total of 252 cases were included: 152 lymphoid, 78 myeloid, and 22 plasma-cell neoplasms. Lymphoid malignancies most commonly presented with cerebellar ataxia (55.3 percent), followed by parkinsonism (18.4 percent) and chorea (11.8 percent), with CNS involvement frequent, including infiltration (33.6 percent) and immune-mediated injury (30.3 percent). Myeloid disorders showed a predominantly hyperkinetic profile, especially generalized chorea (41 percent) and hemichorea or hemiballismus (17.9 percent), while structural CNS disease was rare (2.6 percent) and most cases reflected systemic or treatment-related mechanisms. Plasma-cell neoplasms were characterized mainly by parkinsonism (45.5 percent) and cerebellar syndromes (27.3 percent). Neurological improvement occurred in most patients, particularly after treatment of underlying hematologic disorder. In a cohort of 232 CNS lymphoma patients, prodromal neuropsychiatric features occurred in 9 percent, parkinsonism in 25 percent of those, and 80 percent improved following lymphoma-directed therapy.

**Conclusions:**

Movement disorders in hematologic cancers show distinct, disease-specific profiles, with cerebellar, hyperkinetic, and parkinsonian patterns corresponding to lymphoid, myeloid, and plasma-cell neoplasms. CNS involvement mechanisms differ, providing essential diagnostic and therapeutic guidance.

## Introduction

Hematological malignancies constitute a heterogeneous group of clonal disorders of the blood, bone marrow, lymphatic system, and reticuloendothelial tissues. According to the World Health Organization (WHO) Classification of Haemato-lymphoid Tumours, these diseases are broadly categorized into four major groups: lymphoid neoplasms, myeloid neoplasms, plasma-cell neoplasms, and histiocytic/dendritic-cell neoplasms. The myeloid neoplasm category includes myeloproliferative neoplasms such as polycythemia vera and related disorders. Each of these groups encompasses diverse biological processes, clinical behaviors, and patterns of organ involvement. While these malignancies primarily affect hematopoietic tissues, they frequently involve the central or peripheral nervous system, either directly through infiltration or indirectly through immune-mediated, metabolic, vascular, or treatment-related mechanisms [1,2].

Among the neurological complications associated with hematologic cancers, movement disorders represent an uncommon but clinically significant subset. Their presentations are highly variable and may include parkinsonism, dystonia, chorea, myoclonus, tremor, cerebellar ataxia, opsoclonus-myoclonus, or mixed extrapyramidal syndromes. These manifestations may precede the diagnosis of the underlying malignancy, occur during active disease, or emerge during treatment and survivorship. Pathogenetically, movement disorders in hematologic cancers arise through four principal pathways: paraneoplastic or immune-mediated mechanisms, direct infiltration of the central nervous system (CNS), treatment-related neurotoxicity from cytotoxic agents, immunotherapies, or targeted therapies, and secondary systemic complications such as infections, hyperviscosity, leukostasis, metabolic derangements, or amyloid deposition. Because of this pathogenic diversity, early recognition of a movement disorder in a patient with a hematological neoplasm requires a high index of suspicion and an integrative clinical approach [3,4,5,6,7,8,9,10,11,12].

Despite many case reports, available evidence is inconsistent, limiting recognition of the full clinical spectrum and mechanisms of movement disorders across hematologic malignancies. A systematic review is needed to integrate existing data, clarify phenotype patterns, and support better diagnostic and therapeutic decisions. The primary objective of this systematic review was to synthesize the published information on movement disorders linked to hematological malignancies, structured according to the WHO classification of haemato-lymphoid tumors. By doing so, the review sought to establish an integrated, evidence-based framework to enhance clinical recognition, diagnostic precision, and therapeutic decision-making for movement disorders arising in the context of hematologic malignancies.

## Methods

### Study Design

This systematic review was conducted according to the PRISMA 2020 guidelines and adhered strictly to a predefined methodological protocol. All methodological elements, including construction of the research question, eligibility criteria, search strategies, study selection procedures, data extraction framework, classification systems, and quality appraisal methods, had been finalized prior to initiation of the review. The review protocol was prospectively registered in PROSPERO with registration number CRD420251168610 [13].

### Research Question and PICO Framework

The research question was formulated using the Population-Intervention-Comparator-Outcome paradigm. The population of interest consisted of human patients diagnosed with hematological malignancies as classified by the WHO. The exposure of interest was the onset of a movement disorder temporally or mechanistically attributable to the hematologic disease or its treatment. A formal comparator group was not required because the evidence base comprised descriptive case literature. The outcomes of interest included the movement disorder phenotype, the underlying pathophysiological mechanism, associated clinical and diagnostic features, therapeutic interventions, and neurological or hematological outcomes.

### Eligibility Criteria

Reports were eligible for inclusion if they presented original clinical data involving one or more cases of movement disorders occurring in the context of a confirmed hematological malignancy. Eligible hematologic diagnoses included lymphoid neoplasms, myeloid neoplasms, plasma-cell neoplasms, and histiocytic or dendritic-cell neoplasms. Reports needed to provide a clearly described movement disorder and sufficient information to classify the hematologic condition. Case reports, case series, and observational cohort studies were included without restrictions on language, year of publication, or geographic region. Studies were excluded if they lacked original patient-level data, involved non-hematologic tumors, or described movement disorders fully attributable to unrelated degenerative, genetic, toxic, nutritional, or metabolic conditions. Editorials, reviews without extractable cases, and animal or in-vitro studies were also excluded.

### Information Sources

Multiple electronic databases were searched systematically up to 13 October, 2025. These databases included PubMed, Embase, Scopus, and Google Scholar. Google Scholar was screened to the first 50 pages because of its broad coverage and the high prevalence of non-peer-reviewed material beyond that threshold. In addition to these electronic sources, reference lists of included articles and relevant narrative reviews were examined manually. English translations were obtained for non-English articles when needed.

### Search Strategy

A comprehensive Boolean search strategy was developed and implemented exactly as described in the protocol.

(“Movement Disorder” OR “Movement Disorders” OR Parkinsonism OR Parkinson OR Dystonia OR Chorea OR Choreiform OR Choreoathetosis OR Athetosis OR Myoclonus OR Tremor OR Tics OR “Tourette Syndrome” OR Ataxia OR “Cerebellar Syndrome” OR “Cerebellar Ataxia” OR “Cerebellar Gait” OR Akathisia OR Bradykinesia OR Dyskinesia OR Ballismus OR Hemiballismus OR Rigidity OR “Rest Tremor” OR “Action Tremor” OR Extrapyramidal OR Hypokinetic OR Hyperkinetic OR Stereotypy OR Opsoclonus OR “Ocular Flutter” OR “Gait Disorder”)

AND

(“Hematologic Neoplasm” OR “Hematologic Neoplasms” OR Leukemia OR Lymphoma OR Myeloma OR “Myelodysplastic Syndrome” OR “Myeloproliferative Disorder” OR “Myeloproliferative Disorders” OR “Polycythemia Vera” OR Myelofibrosis)

AND

(“Case Report” OR “Case Reports” OR “Case Series” OR “Clinical Observation” OR “Cohort Study” OR “Cohort Studies” OR “Neurologic Manifestation” OR “CNS Involvement”)*.

### Definition

Movement disorders were categorized based on the standard clinical criteria of the International Parkinson and Movement Disorder Society, encompassing parkinsonism, chorea, dystonia, tremor, myoclonus, ataxia, tics, stereotypies, and other hyperkinetic or hypokinetic extrapyramidal syndromes [14]. Each patient was counted only once. If more than one movement disorder was present, the case was classified according to the main or predominant movement disorder. Other associated movements were noted but not counted separately.

### Study Selection Process

All references identified through database searches were imported into EndNote 21 (Clarivate Analytics, Philadelphia, United States) for duplicate removal. Two reviewers (AJ and VS) independently screened titles and abstracts, and full texts were obtained for all eligible studies. Full-text articles were then reviewed independently by two reviewers (RKG and SP) to determine final inclusion, with disagreements resolved by consensus. The study selection process followed PRISMA guidelines and is presented in a PRISMA flow diagram.

### Data Extraction Procedures

Data were extracted by two reviewers (RK and VP), with uncertainties resolved through consensus with a third reviewer (SS). A standardized 30-variable template was used to capture detailed patient-level information. Demographics, hematologic diagnosis per WHO classification, disease stage, and available molecular or cytogenetic data were recorded. Neurological data included the specific movement-disorder phenotype, timing, progression, associated features, and antibody results. Magnetic resonance imaging (MRI), computed tomography (CT), positron emission tomography–computed tomography (PET-CT), and cerebrospinal fluid (CSF), electrophysiology, and laboratory findings were documented when reported. Hematologic treatments and neurological therapies were extracted, along with outcomes, relapse patterns, mortality, follow-up duration, and time to neurological improvement whenever available.

### Classification as per Pathogenesis

Each case was classified into one of four mechanisms: paraneoplastic immune-mediated, direct CNS infiltration, treatment related, or secondary to systemic complications. Movement disorders were also categorized by phenotype, including parkinsonism, tremor, dystonia, chorea, myoclonus, opsoclonus–myoclonus, ocular flutter, cerebellar ataxia, gait disorders, or mixed presentations.

### Quality Assessment

The methodological quality of all included case reports and case series was evaluated using the Murad et al. assessment framework, which examines four key domains: selection, ascertainment, causality, and reporting. Each case was classified as good, fair, or poor based on the number of domains satisfactorily addressed [15]. Quality Assessment was done by three reviewers (RKG, SP and SS). This task was performed by consensus of the three reviewers.

### Data Synthesis

Because the available evidence consisted predominantly of case reports and case series with substantial clinical heterogeneity, meta-analytic pooling was not feasible. Extracted data were summarized using descriptive statistics. Categorical variables were reported as counts and percentages, and continuous variables as mean, median, range, and interquartile range when available. Cases were grouped by movement-disorder phenotype and mechanism, and neuroimaging patterns, associated features, treatments, and outcomes were analyzed within these categories for the current review.

## Results

**Figure 1:**
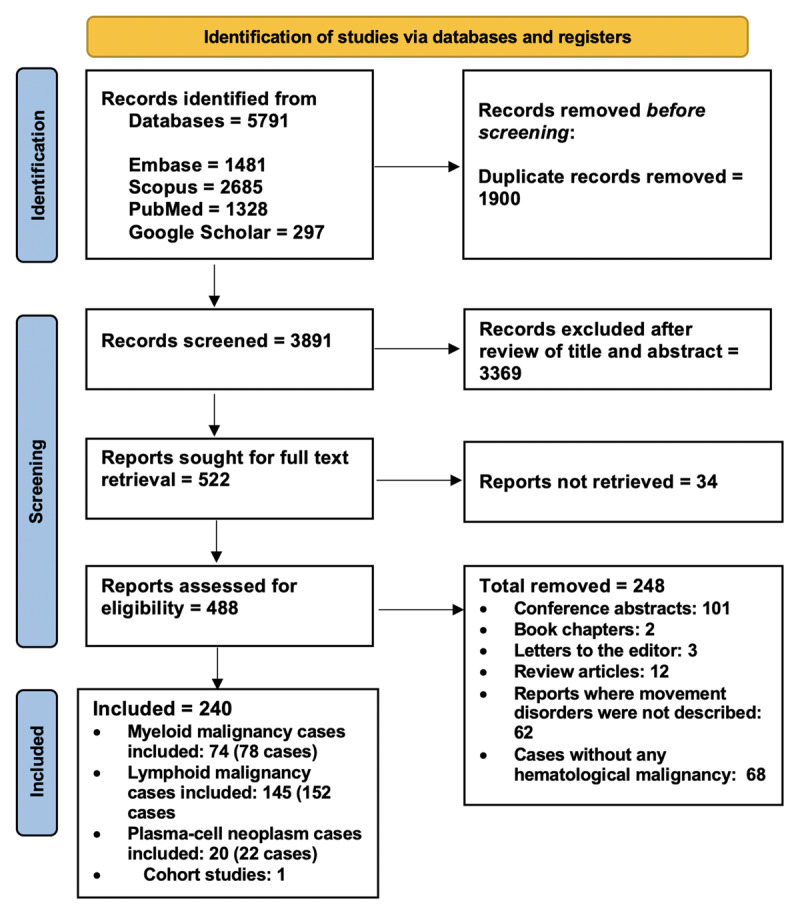
The PRISMA flow diagram summarizing the study selection process. It shows the number of records identified, screened, assessed for eligibility, and finally included in the review.

Across the aggregated 252 cases, which included 152 lymphoid malignancies (145 publications), 78 myeloid malignancies (74 publications), and 22 plasma cell neoplasms (20 publications), the extracted data and their summaries are provided in Table 1 and Supplementary Table 1, Table 2 and Supplementary Table 2, and Table 3 and Supplementary Table 3 respectively. One cohort study that met our PICO-based eligibility criteria was also included. The overall study selection process is depicted in the PRISMA flow chart (Figure 1). All included cases were judged to be of either good or fair methodological quality based on the quality assessment. (Supplementary Table 1, Supplementary Table 2 and Supplementary Table 3) PRISMA checklist is provided as Supplementary Table 4.

**Table 1:** Summary of Clinical and Investigative Features in 152 Lymphoid Malignancy Associated Movement Disorder Cases.


**Age**	Mean: 51 Median: 56 Range: 1–86 IQR: 33

**Sex**	Female (F): 68 (44.7%) Male (M): 84 (55.3%)

**Continent-wise Distribution of Reported Cases (n = 145)**	Europe: 46 (31.7%) Asia: 46 (31.7%) North America: 42 (29.0%) Oceania: 5 (3.4%) South America: 4 (2.8%) Africa: 1 (0.7%)

**Specific lymphoid malignancy diagnosis as per WHO classification**	**Mature B-cell Neoplasms: 82 (53.9%)** *Diffuse Large B-Cell Lymphoma (DLBCL + PCNSL): 62* *Marginal Zone Lymphoma/MALT/SMZL: 5* *Follicular Lymphoma (FL): 2* *Waldenström Macroglobulinemia/Bing-Neel: 3* *Burkitt Lymphoma: 1* *Intravascular Large B-Cell Lymphoma (IVLBCL): 1* *Other Low-Grade B-cell NHL (NOS): 8* **Mature T-cell & NK-cell Neoplasms:14 (9.2%)** *Peripheral T-Cell Lymphoma (PTCL, T-FH, intestinal, NOS): 7* *NK/T-cell Lymphoma (nasal type, CNS-restricted): 4* *Lymphomatoid Granulomatosis (T-cell/EBV associated): 2* *Cutaneous T-cell Lymphoma/Mycosis Fungoides: 1* **Hodgkin Lymphoma:36 (23.7%)** *Classical Hodgkin Lymphoma (all subtypes): 35* *Nodular sclerosis* *Mixed cellularity* *Lymphocyte-depleted* *NOS/Stage IIB, III, IV-B* *Nodular Lymphocyte-Predominant HL (NLPHL): 1* **Precursor Lymphoid Neoplasms: 17 (11.2%)** *B-Acute Lymphoblastic Leukemia/Lymphoblastic Lymphoma (B-ALL/LBL): 17* *Ph+* *Ph–* *FAB L1/L2* *Childhood ALL survivors* *Post-HSCT ALL* **Chronic Lymphocytic Leukemia/SLL: 7 (4.6%)** *CLL (longstanding/treated/with PNS)* *SLL (Stage IV-B)* **Other/Overlap Entities: 5 (3.3%)** *Gray zone lymphoma (B-cell/Hodgkin overlap)* *Composite lymphoma (Hodgkin + B-LPD)* *CML* → *ALL transformation* *Thymoma-associated SPS + remote NHL* *No active lymphoma (stiff-limb syndrome)*

**Staging of the disease**	CNS-only disease: 36 (23.7%)Systemic disease with CNS involvement: 58 (38.2%)Systemic disease without CNS involvement: 19 (12.5%)Relapsed/refractory disease: 22 (14.5%)Post-transplant states: 12 (7.9%)Not staged/insufficient information: 25 (16.4%)

**Molecular Markers**	Lymphoma-defining immunophenotype: 108 (71.1%) *(B-cell, T-cell, NK-cell, HL, ALCL markers showing definite lymphoma biology)* Paraneoplastic/autoimmune antibodies: 18 (11.8%) Molecular/cytogenetic abnormalities: 14 (9.2%) Not reported/insufficient information: 12 (7.9%)

**CNS Involvement**	Direct CNS involvement (tumor infiltration, lymphoma, leukemia, metastasis): 51(33.6%) Paraneoplastic/autoimmune CNS involvement (antibody-mediated, immune): 46 (30.3%) No CNS involvement/other non-tumor causes (drug toxicity, metabolic, infection, PML, radiation, normal): 55 (36.1%)

**Spectrum of Movement Disorders** **(Many Cases Had Multiple Movement Disorders)**	Cerebellar ataxia/cerebellar syndrome (all forms): 84 (55.3%) Parkinsonism (all forms): 28 (18.4%) Chorea/choreoathetosis/hemichorea:18 (11.8%) Myoclonus (all types): 14 (9.2%) Dystonia (all forms): 11 (7.2%) Tremor (action, postural, palatal, oculo-palatal): 7 (4.6%) Dyskinesias (oro-facial, limb, paroxysmal, acute): 7 (4.6%) Stiff-person spectrum (SPS/stiff-limb/PERM): 6 (3.9%) Opsoclonus–myoclonus–ataxia (OMA): 4 (2.6%) Tic disorder: 1 (0.7%) Other rare/mixed syndromes (perseverative behaviour, myorhythmia, etc.): 2 (1.3%)

**Associated Neurological Manifestations**	Cognitive impairment/encephalopathy: 48 (31.6%) Cranial nerve involvement: 42 (27.6%) Cerebellar signs (non-movement): 39 (25.7%) Motor weakness/hemiparesis: 28 (18.4%) Bulbar dysfunction: 24 (15.8%) Sensory loss: 23 (15.1%) Headache/raised ICP signs: 21 (13.8%) Speech/language disturbance (aphasia/dysphasia): 18 (11.8%) Behavioral/psychiatric symptoms: 12 (7.9%) Seizures: 14 (9.2%) Altered gait (non-ataxic): 19 (12.5%) Vertigo (isolated): 9 (5.9%) Brainstem signs: 10 (6.6%) Autonomic dysfunction: 7 (4.6%)

**Onset of Movement Disorders**	Subacute: 82 (53.9%) Acute: 27 (17.8%) Chronic: 21 (13.8%) Acute–subacute: 12 (7.9%) Treatment-triggered: 10 (6.6%)

**CSF Findings** **(Each case assigned to only ONE highest-significance group)**	Normal/near-normal CSF: 46 (30.3%) Lymphocytic pleocytosis ± high protein (Inflammatory CSF without malignant cells): 54 (35.5%) Malignant cells/clonal B-cells/definite CNS lymphoma:11 (7.2%) Oligoclonal bands positive/intrathecal IgG synthesis:18 (11.8%) Infection-related positivity (JCV, EBV, viral PCR, etc.): 6 (3.9%) High protein only (albuminocytologic dissociation/barrier dysfunction): 7 (4.6%) CSF not done/not reported/insufficient information: 10 (6.6%)

**Neuroimaging Findings**	Normal neuroimaging: 41 (27.0%) Cerebellar atrophy/cerebellar structural abnormalities: 22 (14.5%) Basal ganglia/thalamus/brainstem lesions (including caudate, putamen, GP, thalamus, SN, subthalamic nucleus, peduncles): 28 (18.4%) White-matter hyperintensities (T2/FLAIR) leukoencephalopathy: 32 (21.1%) Enhancing mass lesions (tumor, lymphoma, metastasis, hemorrhagic or necrotic): 19 (12.5%) Leptomeningeal disease/meningeal enhancement: 6 (3.9%) Spinal cord involvement (intramedullary, enhancement, extradural tumor): 4 (2.6%) Inferior olive hypertrophy/HOD: 4 (2.6%) Hydrocephalus/mass-effect related ventricular dilation: 3 (2.0%) Subdural hematoma/hygroma: 3 (2.0%) Other rare findings (MRS metabolic change, isolated cortical atrophy, etc.): 3 (2.0%)

**PET Findings**	Systemic FDG-avid lymphadenopathy/systemic lymphoma activity present: 38 (25.0%) No systemic disease on imaging (normal or non-avid): 29 (19.1%) CNS-only PET/SPECT abnormality (cerebellar hyper/hypometabolism; BG uptake): 8 (5.3%) Infection/inflammation/non-malignant extracranial findings (pneumonitis, cardiomyopathy, gastric ulcers, nonspecific rib uptake, chemical toxicity): 6 (3.9%) Organ-specific lesions without systemic lymphoma (lung nodules, gastric lesion, vallecular mass, etc.): 7 (4.6%) Mixed systemic abnormalities (thoracic/abdominal nodes + other lesions): 5 (3.3%) Negative initial imaging → later new systemic disease detected (Delayed PET positivity/new nodal or pulmonary lesions): 5 (3.3%) Imaging performed but inconclusive: 6 (3.9%) No PET-CT/CT/systemic imaging performed or not reported: 48 (31.6%)

**Treatment Given**	Systemic chemotherapy: 83 (54.6%) High-dose methotrexate–based therapy: 41 (27.0%) Rituximab-containing regimens: 57 (37.5%) Radiotherapy: 38 (25.0%) Stem-cell transplant: 29 (19.1%) Immunotherapy/biologics: 17 (11.2%) Steroids/immunosuppression only: 21 (13.8%) No treatment/watch-and-wait: 12 (7.9%) Surgery only: 7 (4.6%) **Targeted therapy** Rituximab: 29 (19%) Brentuximab vedotin: 2 (1%) Pembrolizumab: 1 (1%) Alemtuzumab: 1 (1%) Dasatinib: 2 (1%) None: 117 (77%) **HSCT/BMT** No HSCT: 132 (86.8%) Autologous HSCT: 8 (5.3%) Allogeneic HSCT: 6 (3.9%) Both Auto + Allo: 1 (0.7%) BMT (unspecified): 5 (3.3%) **Other medical treatments** Steroids: 41 (27%) IVIG: 34 (22%) Plasmapheresis: 12 (8%) Levodopa/dopaminergic drugs: 29 (19%) Benzodiazepines: 24 (16%) Antiepileptics (LEV/VAL/CBZ/GABA): 31 (20%) Antipsychotics (HAL/TBZ): 17 (11%) Anticholinergics (trihexyphenidyl/benztropine): 9 (6%) Botulinum toxin: 1 (1%) Neurosurgery: 5 (3%) Supportive/rehab only: 48 (32%) No treatment reported: 39 (26%)

**Response to treatment**	Marked/complete improvement: 63 (41%) Partial improvement: 42 (28%) Stabilization only: 18 (12%) No improvement/worsening: 29 (19%)

**Follow up Period**	<1 month: 20 (13.2%) 1–3 months: 24 (15.8%) 3–6 months: 28 (18.4%) 6–12 months: 30 (19.7%) 1–2 years: 25 (16.4%) 2–5 years: 17 (11.2%) >5 years: 8 (5.3%)

**Proposed Mechanisms of Movement Disorders**	Direct CNS infiltration: 41 (27%) Paraneoplastic/autoimmune: 52 (34%) Drug-induced toxicity: 26 (17%) Infectious (PML/viral/fungal): 9 (6%) Radiation-related toxicity: 7 (5%) Metabolic/nutritional: 4 (2.6%) Vascular/ischemic: 5 (3.3%) Structural/degenerative (HOD etc.): 5 (3.3%) Reversible functional: 3 (2%)


ABMT – Autologous Bone Marrow Transplant; ABVD – Adriamycin (Doxorubicin), Bleomycin, Vinblastine, Dacarbazine; ACTH – Adrenocorticotropic Hormone; ALL – Acute Lymphoblastic Leukemia; allo-HSCT – Allogeneic Hematopoietic Stem-Cell Transplant; Ara-C – Cytarabine; ASCT – Autologous Stem-Cell Transplant; BG – Basal Ganglia; BMT – Bone Marrow Transplant; B-NHL – B-cell Non-Hodgkin Lymphoma; B-ALL/LBL – B-Acute Lymphoblastic Leukemia/Lymphoblastic Lymphoma; CBZ – Carbamazepine; cGVHD – Chronic Graft-Versus-Host Disease; CLL – Chronic Lymphocytic Leukemia; CNS – Central Nervous System; COEP/OCEP – Cyclophosphamide, Vincristine, Etoposide, Prednisolone/Oncovin, Cyclophosphamide, Etoposide, Prednisolone; COP – Cyclophosphamide, Vincristine, Prednisone; CRMP-5 – Collapsin Response Mediator Protein-5; CyA – Cyclosporine A; DLBCL – Diffuse Large B-Cell Lymphoma; ENKL – Extranodal NK/T-Cell Lymphoma; EVAP – Etoposide, Vinblastine, Adriamycin, Prednisone; FAB – French-American-British Classification; FLAIR – Fluid-Attenuated Inversion Recovery; FL – Follicular Lymphoma; FOG – Freezing of Gait; GABA – Gamma-Aminobutyric Acid; GAD – Glutamic Acid Decarboxylase; GP – Globus Pallidus; GZL – Gray Zone Lymphoma; HOD – Hypertrophic Olivary Degeneration; HL – Hodgkin Lymphoma; HRCT – High-Resolution Computed Tomography; HSCT – Hematopoietic Stem-Cell Transplant; IQR – Interquartile Range; IT-MTX – Intrathecal Methotrexate; IVLBCL – Intravascular Large B-Cell Lymphoma; IVIG – Intravenous Immunoglobulin; JCV – John Cunningham Virus; LEV – Levetiracetam; LPC – Lymphoplasmacytic Cells; MALT – Mucosa-Associated Lymphoid Tissue; MF – Mycosis Fungoides; MMF – Mycophenolate Mofetil; MRS – Magnetic Resonance Spectroscopy; MTX – Methotrexate; MOPP – Mustine, Oncovin, Procarbazine, Prednisone; MRI – Magnetic Resonance Imaging; NAD – No Abnormality Detected; NHL – Non-Hodgkin Lymphoma; NK/T – Natural Killer/T-Cell; NOS – Not Otherwise Specified; OMA – Opsoclonus–Myoclonus–Ataxia; PCD – Paraneoplastic Cerebellar Degeneration; PCR – Polymerase Chain Reaction; PCNSL – Primary CNS Lymphoma; PCP – Pneumocystis Pneumonia; PD-1 – Programmed Cell Death Protein 1; PERM – Progressive Encephalomyelitis with Rigidity and Myoclonus; PET-CT – Positron Emission Tomography–Computed Tomography; Ph+ – Philadelphia Chromosome Positive; Ph– – Philadelphia Chromosome Negative; PML – Progressive Multifocal Leukoencephalopathy; PTCL – Peripheral T-Cell Lymphoma; R-CHOP – Rituximab, Cyclophosphamide, Doxorubicin, Vincristine, Prednisone; R-CVP – Rituximab, Cyclophosphamide, Vincristine, Prednisone; R-EPOCH – Rituximab, Etoposide, Prednisone, Vincristine, Cyclophosphamide, Doxorubicin; R-MPV – Rituximab, Methotrexate, Procarbazine, Vincristine; SLL – Small Lymphocytic Lymphoma; SMZL – Splenic Marginal Zone Lymphoma; SN – Substantia Nigra; SPS – Stiff-Person Syndrome; SUV – Standardized Uptake Value; TBZ – Tetrabenazine; TMP-SMX – Trimethoprim–Sulfamethoxazole; T-FH – T-Follicular Helper Cell; VP Shunt – Ventriculoperitoneal Shunt; WM – Waldenström Macroglobulinemia; WBRT – Whole-Brain Radiotherapy; WHO – World Health Organization; WMH – White-Matter Hyperintensity.

The epidemiological profile demonstrated broad demographic variation across the 252 cases. Patient age ranged from 1 to 86 years, with mean ages of 51 years in lymphoid malignancies, 57.1 years in myeloid malignancies, and 60.3 years in plasma-cell neoplasms. A slight male predominance was observed in lymphoid cases (55.3 percent), whereas myeloid (46.1 percent male) and plasma-cell cases (46.2 percent male) showed near-equal sex distribution. (Table 1 and Supplementary Table 1, Table 2 and Supplementary Table 2, and Table 3 and Supplementary Table 3)

Geographically, cases were globally distributed. Europe and Asia each contributed 31.7 percent of lymphoid cases. Myeloid cases were most frequently reported from Asia (40.8 percent), followed by North America (30.3 percent). Plasma-cell neoplasms were most commonly reported from North America (47.8 percent), followed by Asia (30.4 percent) and Europe (21.7 percent). (Table 1 and Supplementary Table 1, Table 2 and Supplementary Table 2, and Table 3 and Supplementary Table 3)

Diagnostic distribution reflected the underlying epidemiology of hematologic cancers. Among lymphoid neoplasms, mature B cell malignancies dominated, accounting for 53.9 percent, while Hodgkin lymphoma comprised 23.7 percent and precursor lymphoid neoplasms contributed 11.2 percent. Myeloid disorders were driven by myeloproliferative neoplasms at 59 percent, with polycythemia vera alone representing 42.3 percent. Acute myeloid leukemia accounted for 28.2 percent of myeloid cases. Plasma-cell neoplasms were overwhelmingly represented by multiple myeloma, which constituted 86.4 percent of the 22 cases. (Table 1 and Supplementary Table 1, Table 2 and Supplementary Table 2, and Table 3 and Supplementary Table 3)

### Lymphoid Malignancy

Among the 152 lymphoid malignancy cases, movement disorders and CNS involvement formed the core neurological manifestations. Cerebellar ataxia was the predominant movement disorder and occurred in 84 cases, which represented 55.3 percent of the cohort. Parkinsonism was reported in 28 cases or 18.4 percent, while chorea and choreoathetosis were present in 18 cases or 11.8 percent. Myoclonus was documented in 14 cases or 9.2 percent, dystonia in 11 cases or 7.2 percent, and tremor or dyskinesias in 7 cases each, accounting for 4.6 percent. Rare movement disorders included stiff-person spectrum disorders in 6 cases or 3.9 percent and opsoclonus-myoclonus-ataxia in 4 cases or 2.6 percent. Many patients had more than one movement disorder phenotype, reflecting mixed cerebellar and extrapyramidal involvement. (Table 1 and Supplementary Table 1)

CNS involvement was also substantial. Direct CNS infiltration by lymphoma was documented in 51 cases or 33.6 percent, while paraneoplastic or autoimmune CNS injury accounted for 46 cases or 30.3 percent. Only 55 cases or 36.1 percent lacked malignancy related CNS involvement. Cognitive impairment occurred in 48 cases or 31.6 percent and cranial nerve abnormalities in 42 cases or 27.6 percent. CSF analysis showed abnormalities in approximately 70 percent of tested patients, including inflammatory pleocytosis in 54 cases or 35.5 percent, malignant cells in 11 cases or 7.2 percent, and intrathecal IgG synthesis in 18 cases or 11.8 percent. Neuroimaging revealed CNS pathology in the majority, with white matter changes seen in 32 cases or 21.1 percent and basal ganglia or brainstem lesions in 28 cases or 18.4 percent. Together, these findings demonstrated that lymphoid malignancies produced extensive cerebellar, extrapyramidal, and immunological involvement within the CNS. (Table 1 and Supplementary Table 1)

Neurological manifestations in lymphoid malignancies were primarily treated with definitive tumor-directed therapy, most commonly systemic chemotherapy (54.6 percent), rituximab-containing regimens (37.5 percent), high-dose methotrexate–based therapy (27.0 percent), and radiotherapy (25.0 percent), while stem-cell transplantation was required in a minority (19.1 percent). Adjunct symptomatic neurological therapy was selectively used and included corticosteroids (27 percent), antiepileptic drugs (20 percent), levodopa or other dopaminergic agents (19 percent), benzodiazepines (16 percent), antipsychotics or dopamine-blocking agents (11 percent), anticholinergics (6 percent), intravenous immunoglobulin (22 percent), and plasmapheresis (8 percent), whereas supportive care alone was sufficient in 32 percent and no neurological treatment was reported in 26 percent. Targeted therapies were uncommon overall, with rituximab used in 19 percent and other biologics each in about 1 percent, and most patients did not undergo transplantation (86.8 percent). Clinical outcomes were mechanism-dependent: marked or complete neurological improvement occurred in 41 percent, partial improvement in 28 percent, stabilization in 12 percent, and no improvement or worsening in 19 percent. Collectively, these findings indicate that neurological prognosis in lymphoid malignancy–associated movement disorders is largely determined by reversibility of the underlying pathophysiology and response to oncologic therapy rather than symptomatic neurologic treatment alone. (Table 1 and Supplementary Table 1)

### Myeloid Malignancy

Across 78 reported cases of myeloid hematological malignancies, hyperkinetic movement disorders formed the dominant clinical spectrum, with generalized chorea documented in 32 patients (41 percent) and hemichorea or hemiballismus in 17.9 percent, establishing choreiform syndromes as the hallmark presentation. Cerebellar ataxia occurred in 19.2 percent, parkinsonism in 9 percent, and myoclonus in 7.7 percent, while rarer phenomena such as neuromyotonia, myorhythmia, and blepharospasm collectively accounted for 3.8 percent. (Table 2 and Supplementary Table 2)

**Table 2:** Summary of 78 Reported Cases of Movement Disorders in Myeloid Malignancies.


**Age**	NA: 1 Mean: 57.1 Median: 62 Range: 8–96 IQR: 31

**Sex**	Male (M): 36 (46.1%) Female (F): 41 (52.6%) Not available (NA): 1 (1.3%)

**Continent-wise Distribution of Reported Cases (n = 74)**	Asia (incl. Middle East & Asian Russia): 31 (40.8%) North America: 23 (30.3%) Europe: 18 (23.7%) South America: 2 (2.6%) Oceania: 1 (1.3%) Africa: 1 (1.3%)

**Specific Myeloid Malignancy Diagnosis as per WHO Classification**	Myeloproliferative Neoplasms (MPN): 46 (59.0%) Polycythemia Vera (PV): 33 (42.3%) Essential Thrombocythemia (ET): 3 (3.8%) Primary Myelofibrosis (PMF): 3 (3.8%) MPN-unclassifiable/variants: 7 (9.0%) Chronic Myeloid Leukemia (CML): 7 (9.0%) Acute Myeloid Leukemia (AML): 22 (28.2%) Myelodysplastic Syndrome (MDS): 2 (2.5%) MDS/MPN Overlap: 1 (1.3%)

**Staging of the disease**	Newly diagnosed: 27 (34.6%) Long-standing/chronic: 20 (25.6%) Post-HSCT: 17 (21.8%) During therapy: 12 (15.4%) Relapse/deterioration: 2 (2.6%)

**Molecular Markers**	JAK2-positive: 38 (48.7%) BCR-ABL–positive: 4 (5.1%) AML-mutation profiles: 6 (7.7%) No molecular data (pre-JAK2/not reported): 20 (25.6%) Other/HSCT-related/mixed: 10 (12.8%)

**CNS Involvement**	No CNS leukemia: 56 (71.8%) Drug-induced neurotoxicity: 14 (17.9%) CNS infection: 3 (3.8%) CNS toxoplasmosis JC-virus PML Infection-related microangiopathy Vascular/Stroke/Hemorrhage: 5 (6.4%) CNS-GVHD: 3 (3.8%) True CNS leukemia: 2 (2.6%)

**Spectrum of Movement Disorders** **(Many Cases Had Multiple Movement Disorders)**	Generalized chorea: 32 (41.0%) Hemichorea/hemiballismus: 14 (17.9%) Cerebellar ataxia/cerebellar syndrome: 15 (19.2%) Parkinsonism: 7 (9.0%) Myoclonus/myoclonic epilepsy/OMS: 6 (7.7%) Dystonia: 4 (5.1%) Other rare hyperkinetic movement disorders: 3 (3.8%) Polyminimyoclonus/neuromyotonia Myorhythmia Blepharospasm Ocular motor disorders: 3 (3.8%) Mixed (complex) presentations: 2 (2.6%)

**Associated Neurological Manifestations**	Cognitive/behavioral: 18 (23.1%) Speech impairment: 16 (20.5%) Sensory/neuropathy: 10 (12.8%) Autonomic dysfunction: 8 (10.3%) Cerebellar/vestibular-associated: 9 (11.5%) Weakness/pyramidal: 7 (9.0%) Miscellaneous systemic neurologic: 10 (12.8%)

**Onset of Movement Disorders**	Acute: 20 (25.6%) Subacute: 25 (32.1%) Chronic/Gradual: 15 (19.2%) Treatment-related: 18 (23.1%)

**CSF Findings** **(Each case assigned to only ONE highest-significance group)**	Normal CSF: 39 (50.0%) CSF not done/not reported: 29 (37.2%) Mild abnormal CSF: 10 (12.8%) Infectious CSF positivity: 3 (3.8%) CNS leukemic meningitis: 1 (1.3%)

**Neuroimaging Findings**	Normal neuroimaging: 27 (34.6%) Chronic ischemia/microangiopathy/atrophy: 17 (21.8%) Acute infarcts/vascular lesions: 9 (11.5%) Basal ganglia/deep gray abnormalities: 9 (11.5%) Cerebellar lesions: 5 (6.4%) Multifocal/diffuse WM lesions: 6 (7.7%) Ring-enhancing/infective–inflammatory lesions: 2 (2.6%) Hemorrhagic lesions: 2 (2.6%) CNS leukemic infiltration: 1 (1.3%)

**PET Findings**	PET/SPECT not done/not available: 52 (66.7%) Normal PET/SPECT/CT-CAP: 12 (15.4%) Abnormal PET/SPECT: 6 (7.7%) Thalamic hyperperfusion (SPECT) Basal ganglia hyperperfusion (SPECT) Diffuse cortical hypoperfusion (SPECT) → improved with treatment FDG-PET: Putamen + precentral hypermetabolism → resolved Reversible PET/SPECT abnormalities PET/SPECT abnormalities reversible Perfusion/venous abnormalities: 3 (3.8%) Structural CT/MRI mentioned in PET section: 5 (6.4%)

**Treatment Given**	Phlebotomy/venesection ± aspirin: 27 (34.6%) Hydroxyurea-based therapy: 20 (25.6%) HSCT/BMT/immunosuppression: 14 (17.9%) TKIs: 6 (7.7%) AML multi-agent chemotherapy: 5 (6.4%) APL therapy (ATRA/ATO): 2 (2.6%) Immunomodulators (thalidomide/lenalidomide): 3 (3.8%) Supportive only: 1 (1.3%) **Targeted Therapy: 2 (2.6%)** Protein kinase inhibitor Azacitidine + azathioprine (stopped) **HSCT** Allogeneic HSCT: 10 (12.8%) Autologous HSCT: 1 (1.3%) HSCT × 2: 1 (1.3%) No HSCT: 66 (84.6%) **Medical Therapy** Supportive/none: 33 (42.3%) Anti-chorea/dopamine-blockers: 10 (12.8%) Immunotherapy (steroids/IVIG/PLEX/RTX): 9 (11.5%) Anti-seizure/VNS: 4 (5.1%) Levodopa therapy: 5 (6.4%) Vestibular rehab: 3 (3.8%) Infection-focused neurotherapy: 2 (2.6%) Botulinum toxin: 2 (2.6%) Vitamin/metabolic therapy: 3 (3.8%) Anticoagulation: 1 (1.3%) Ommaya reservoir therapy: 1 (1.3%) Stopping offending drug: 5 (6.4%)

**Response to treatment**	Complete resolution: 23 (29.5%) Marked improvement: 21 (26.9%) Partial improvement: 17 (21.8%) Poor/minimal/no improvement: 6 (7.7%) Improvement after hematologic correction: 7 (9.0%) Supportive only (non-specific): 4 (5.1%)

**Follow up Period**	<1 month: 7 (9.0%) 1–3 months: 15 (19.2%) 3–6 months: 18 (23.1%) 6–12 months: 14 (17.9%) 1–2 years: 10 (12.8%) 2–5 years: 7 (9.0%) ≥5 years: 3 (3.8%) Not stated: 4 (5.1%)

**Proposed Mechanisms of Movement Disorders**	Hyperviscosity-related: 36 (46.1%) Drug-induced toxicity: 17 (21.8%) Immune-mediated/GVHD: 10 (12.8%) JAK2-specific (non-hyperviscosity): 4 (5.1%) Infection-related: 5 (6.4%) Hemorrhagic/vascular fragility: 2 (2.6%) Degenerative/structural: 2 (2.6%) Metallotoxic: 1 (1.3%) Unclear/not stated: 1 (1.3%)


AML — Acute Myeloid Leukemia; APML — Acute Promyelocytic Leukemia; ATRA — All-trans Retinoic Acid; ATO — Arsenic Trioxide; BCR-ABL — Breakpoint Cluster Region–Abelson Fusion Gene; BMT — Bone Marrow Transplant; CAP — Chest–Abdomen–Pelvis; CML — Chronic Myeloid Leukemia; CNS — Central Nervous System; CSF — Cerebrospinal Fluid; CT — Computed Tomography; ET — Essential Thrombocythemia; FDG-PET — Fluorodeoxyglucose Positron Emission Tomography; F — Female; GVHD — Graft-Versus-Host Disease; HSCT — Hematopoietic Stem Cell Transplantation; HU — Hydroxyurea; IQR — Interquartile Range; IVIG — Intravenous Immunoglobulin; JAK2 — Janus Kinase 2; M — Male; MDS — Myelodysplastic Syndrome; MPN — Myeloproliferative Neoplasm; MRI — Magnetic Resonance Imaging; NA — Not Available; OMS — Opsoclonus–Myoclonus Syndrome; PET — Positron Emission Tomography; PLEX — Plasma Exchange; PMF — Primary Myelofibrosis; PML — Progressive Multifocal Leukoencephalopathy; PV — Polycythemia Vera; RTX — Rituximab; SPECT — Single-Photon Emission Computed Tomography; TKI — Tyrosine Kinase Inhibitor; VNS — Vagus Nerve Stimulation; WM — White Matter.

Structural central nervous system leukemia was distinctly uncommon at 2.6 percent, and most patients, 71.8 percent, showed no demonstrable structural central nervous system involvement. Instead, pathophysiology was largely secondary, with drug induced neurotoxicity responsible for 17.9 percent of cases, vascular complications for 6.4 percent, and infectious etiologies for 3.8 percent. These data indicate that neurological manifestations in myeloid malignancies are typically mediated by systemic metabolic, vascular, immune, or treatment related mechanisms rather than direct leukemic infiltration. (Table 2 and Supplementary Table 2)

Neurological manifestations in myeloid hematological malignancies were most frequently managed by treating the underlying hematologic disorder, most commonly with phlebotomy ± aspirin (34.6 percent) or hydroxyurea-based therapy (25.6 percent). Specific neurological therapy was unnecessary in 42.3 percent of cases, indicating strong reversibility once systemic pathology was corrected. When symptomatic treatment for movement disorders was required, it most often consisted of anti-chorea or dopamine-blocking agents (12.8 percent), immunotherapy (11.5 percent), or targeted symptomatic modalities such as levodopa (6.4 percent) and botulinum toxin (2.6 percent). Overall, 78.2 percent of patients demonstrated neurological improvement, including complete resolution in 29.5 percent, marked improvement in 26.9 percent, and partial improvement in 21.8 percent. HSCT-related syndromes represented a minority (allogeneic 12.8 percent, autologous 1.3 percent), and targeted therapies were rarely required (2.6 percent), underscoring that most neurological complications are secondary phenomena responsive primarily to correction of the hematologic disorder. (Table 2)

### Plasma-Cell Neoplasms

In the 22 reported cases of plasma-cell neoplasms associated with movement disorders. Parkinsonism was the leading movement-disorder phenotype and was documented in 10 cases, representing 45.5 percent of the cohort. Cerebellar syndromes or ataxia appeared in 6 cases or 27.3 percent, while tremor disorders were recorded in 4 cases or 18.2 percent. A further 6 cases, also 27.3 percent, showed other movement-disorder presentations including tardive-dyskinesia–like syndromes, multifocal myoclonus, dystonia, restless legs syndrome, stiff-person syndrome, and non-specific involuntary movements. Mixed movement disorders were common and often coexisted with peripheral or central neurological deficits. (Table 3 and Supplementary Table 3)

**Table 3:** Summary of 22 Cases of Myeloma-Associated Movement Disorders.


**Age**	Mean: 60.6 Median: 65 Mode: 65 Range: 31–78 IQR: 13

**Sex**	Male: 10 (45.45%) Female: 12 (54.55%)

**Continent-wise Distribution of Reported Cases (n = 20)**	North America: 9 (45%) Asia: 6 (30%) Europe: 5 (25%)

**Specific hematological malignancy diagnosis as per WHO classification**	Multiple Myeloma: 19 (86.4%) Smoldering Multiple Myeloma: 1 (4.5%) Plasmacytoma: 1 (4.5%) IgM MGUS → MM: 1 (4.5%)

**Staging of the disease**	Staged (ISS/R-ISS available): 7 (31.8%) Progressive/Relapsed/Refractory: 8 (36.4%) Long remission (post-BMT): 1 (4.5%) Systemic involvement: 1 (4.5%) Not reported/Not stated: 5 (22.7%)

**Molecular Markers**	Cytogenetic/FISH abnormalities: 7 (31.8%) Monoclonal protein abnormalities: 9 (40.9%) Not reported: 6 (27.3%)

**CNS Involvement**	No CNS infiltration: 12 (54.5%) Autoimmune/Paraneoplastic: 5 (22.7%) Infectious: 2 (9.1%) Other CNS diseases (leukoencephalopathy/neurodegenerative): 3 (13.6%)

**Spectrum of Movement Disorders** **(Many Cases Had Multiple Movement Disorders)**	Parkinsonism:10 (45.5%) Cerebellar syndromes/Ataxia: 6 (27.3%) Tremor disorders: 4(18.2%) Other movement disorders : 6 (27.3%) (List of all individual “other” disorders): *Tardive dyskinesia–like extrapyramidal syndrome (EPS)* *Multifocal myoclonus* *Restless legs syndrome (RLS)* *Stiff-person syndrome (SPS)* *Involuntary movements (not otherwise specified)* *Dystonia*

**Associated Neurological Manifestations**	Cognitive/Encephalopathic: 7 (31.8%) Sensory/Peripheral neuropathy: 6 (27.3%) Cranial nerve involvement: 3 (13.6%) Pyramidal signs: 3 (13.6%) Myoclonic/stiffness episodes: 1 (4.5%) Hydrocephalus/mass effect: 1 (4.5%) None: 2 (9.1%)

**Onset of Movement Disorders**	Long latency (>2 years): 10 (47.6%) Intermediate latency (1–2 years): 5 (23.8%) Short latency (<6 months): 4 (19.0%) Not applicable: 1 (4.8%) Symptoms preceded MM diagnosis: 3 cases

**CSF Findings**	Normal: 7 (31.8%) Abnormal (non-infectious): 4 (18.2%) Infectious: 2 (9.1%) Negative panels: 3 (13.6%) Not done/Not reported: 6 (27.3%)

**Neuroimaging Findings**	Normal: 7 (31.8%) Cerebellar pathology: 5 (22.7%) White matter changes: 3 (13.6%) Cortical ribboning/degenerative: 2 (9.1%) Not done/Not reported: 4 (18.2%)

**PET Findings**	Normal/Negative PET-CT: 4 (18.2%) Other abnormality (non-MM): 1 (4.5%) Not done/Not performed: 17 (77.3%)

**Treatment Given**	CAR-T therapy: 5 (22.7%) Bortezomib-based regimens: 5 (22.7%) Lenalidomide-based regimens: 4 (18.2%) Melphalan-based regimens: 4 (18.2%) Radiotherapy/Surgery: 2 (9.1%) Multiple prior regimens: 1 (4.5%) Supportive/Referred: 1 (4.5%) **Targeted Therapy** CAR-T: 2 (9.1%) Ruxolitinib: 2 (9.1%) Bortezomib: 2 (9.1%) IMiDs: 3 (13.6%) Interferon-α: 1 (4.5%) None/Not reported: 11 (50.0%) **HSCT** Autologous HSCT: 8 (36.4%) **Neurological Treatment** Immunotherapy (IVIG/steroids/anakinra/cyclophosphamide): 7 (31.8%) Movement-disorder drugs (levodopa, amantadine, primidone, propranolol, clonazepam, DBS/thalamotomy): 6 (27.3%) Drug withdrawal/dose reduction (stopping LEN/thalidomide/pregabalin, TMP-SMX reduction): 4 (18.2%) Supportive care only: 3 (13.6%) Neurosurgery (decompression/ventriculostomy): 2 (9.1%) Other symptomatic drugs (amitriptyline, piracetam, prednisolone): 2 (9.1%)

**Response to Treatment**	Complete resolution: 6 (27.3%) Marked improvement: 7 (31.8%) Partial improvement: 5 (22.7%) Minimal benefit: 1 (4.5%) No improvement/progression: 3 (13.6%)

**Follow up Period**	<3 months: 5 (22.7%) 3–12 months: 7 (31.8%) >1 year: 8 (36.4%) Not reported/not stated: 2 (9.1%)

**Proposed Mechanisms of Movement Disorders**	Drug-induced: 7 (31.8%) Immune/cytokine-mediated: 6 (27.3%) Paraneoplastic/autoimmune: 5 (22.7%) Mass lesion: 1 (4.5%) Infectious: 1 (4.5%) Degenerative: 1 (4.5%) Metabolic/nutritional: 1 (4.5%)


BMT: Bone Marrow Transplantation; CAR-T: Chimeric Antigen Receptor T-cell therapy; CNS: Central Nervous System; CSF: Cerebrospinal Fluid; DBS: Deep Brain Stimulation; EPS: Extrapyramidal Syndrome; FISH: Fluorescence In Situ Hybridization; HSCT: Hematopoietic Stem Cell Transplantation; IgM MGUS: Immunoglobulin M Monoclonal Gammopathy of Undetermined Significance; IMiDs: Immunomodulatory Drugs; IQR: Interquartile Range; ISS: International Staging System; IVIG: Intravenous Immunoglobulin; LEN: Lenalidomide; MM: Multiple Myeloma; PET-CT: Positron Emission Tomography–Computed Tomography; R-ISS: Revised International Staging System; RLS: Restless Legs Syndrome; SPS: Stiff-Person Syndrome; TMP-SMX: Trimethoprim-Sulfamethoxazole; WHO: World Health Organization.

CNS involvement varied in mechanism and severity. Twelve cases, or 54.5 percent, had no detectable CNS infiltration. Immune or paraneoplastic processes were present in 5 cases or 22.7 percent, and infectious CNS involvement, including opportunistic pathogens, was observed in 2 cases or 9.1 percent. Three cases demonstrated other CNS pathology such as leukoencephalopathy or degenerative processes. CSF abnormalities were documented in 31.8 percent of patients, with infectious positivity in 9.1 percent. Neuroimaging was abnormal in nearly half of the cases, with cerebellar pathology in 22.7 percent, white-matter signal changes in 13.6 percent, and cortical or degenerative patterns in 9.1 percent. Overall, plasma-cell neoplasms produced a distinct movement-disorder profile dominated by parkinsonism, with CNS involvement arising from immune, infectious, or structural mechanisms rather than direct myeloma infiltration. (Table 3 and Supplementary Table 3)

Across 22 cases, systemic therapy included CAR-T and bortezomib regimens (each 22.7 percent), lenalidomide or melphalan regimens (each 18.2 percent), and surgery or radiotherapy (9.1 percent), with autologous transplantation in 36.4 percent. Neurologic treatment comprised immunotherapy in 31.8 percent, symptomatic drugs in 27.3 percent (levodopa, amantadine, propranolol, primidone, clonazepam, DBS/thalamotomy), and drug withdrawal in 18.2 percent (lenalidomide, thalidomide, pregabalin or co-trimoxazole). Outcomes showed complete resolution in 27.3 percent, marked improvement in 31.8 percent, partial improvement in 22.7 percent, and progression in 13.6 percent, with drug-induced (31.8 percent) and immune-mediated (27.3 percent) mechanisms predominating. (Table 3)

### Cohort study

In a cohort of 232 CNS lymphoma patients, 20 (9%) had prodromal neuropsychiatric symptoms, and Parkinsonism appeared in 5 of these 20 cases (25%). All five had deep grey-matter involvement, predominantly the basal ganglia and corpus callosum, explaining the dopaminergic dysfunction. Parkinsonism developed over weeks to months and often mimicked rapidly progressive atypical parkinsonian syndromes. Importantly, four of the five patients (80%) improved after lymphoma-directed therapy, indicating reversibility once the infiltrative lesion was treated [16].

## Discussion

This systematic review identified several distinctive and clinically important patterns that clarify how hematological malignancies lead to movement disorders through diverse biological mechanisms. Each WHO-defined category of hematologic disease demonstrated its own characteristic movement-disorder profile, shaped by inherent differences in tumor behavior, immune interactions, vascular effects, and modes of CNS involvement. Lymphoid malignancies showed a strong tendency toward cerebellar dysfunction, often accompanied by features suggestive of paraneoplastic or immune-mediated injury, reflecting the immunological complexity of B cell and Hodgkin lymphomas. Myeloid neoplasms, in contrast, displayed a predominantly hyperkinetic pattern, particularly with choreiform movement disorders, consistent with the influence of systemic disturbances such as hyperviscosity, microcirculatory compromise, and cytokine-mediated metabolic stress rather than direct CNS invasion. Plasma-cell neoplasms demonstrated yet another pattern, most notably an association with parkinsonian features, suggesting selective vulnerability of basal ganglia pathways possibly related to amyloid deposition, treatment effects, or immune dysregulation. A further key observation was the fundamental difference in how these malignancies involve the CNS: lymphoid cancers frequently produced direct structural involvement, while myeloid and plasma-cell disorders more often generated neurological manifestations through indirect systemic, immune, treatment-related, or infectious mechanisms.

### Underlying Mechanisms

The pathogenetic patterns observed across the three WHO-defined hematologic malignancy groups demonstrate that movement disorders arise through distinct and biologically coherent mechanisms. In lymphoid neoplasms, two pathways predominated: direct central nervous system infiltration and immune-mediated paraneoplastic processes. The prominence of direct infiltration aligns with contemporary evidence showing that diffuse large B-cell lymphoma and primary CNS lymphoma exhibit marked neurotropism, frequently targeting the cerebellum, deep gray nuclei, and brainstem. Recent neuropathological and imaging-based studies confirm that even small-volume lymphomatous deposits in these regions can produce early cerebellar or extrapyramidal syndromes. The substantial proportion of immune-mediated cases is equally notable. Modern paraneoplastic immunology literature describes that autoreactive B-cell clones in lymphoid cancers can generate onconeural antibodies, leading to paraneoplastic cerebellar degeneration, opsoclonus–myoclonus, or basal ganglia dysfunction through antibody-driven synaptic and neuronal injury. Together, these converging findings emphasize that both structural invasion and dysregulated tumor-driven immunity are central to the pathogenesis of movement disorders in lymphoid malignancies [17].

In contrast, the movement disorders seen in myeloid neoplasms predominantly reflected systemic and metabolic mechanisms. Hyperviscosity, leukostasis, and cytokine-mediated injury disrupted basal ganglia microcirculation and produced high rates of chorea. The rarity of direct CNS leukemia further supports the concept that myeloid malignancies exert their neurological effects primarily through distal vascular or metabolic pathways, not direct parenchymal invasion [18].

Plasma-cell neoplasms demonstrated yet another pathogenesis mechanism. Amyloid deposition, immune disturbances, and treatment-induced dopaminergic vulnerability were major contributors. The predominance of parkinsonism suggests preferential injury to basal ganglia circuits, potentially through amyloid infiltration, autoimmune inflammation, or medication-related dopaminergic suppression. Infectious mechanisms, particularly in immunocompromised patients receiving proteasome inhibitors or steroids, also contributed to CNS dysfunction [19].

Drug-induced movement disorders are an important diagnostic consideration in hematological malignancies. They typically show a clear temporal relationship to drug initiation or dose escalation, with acute or subacute onset and improvement after drug withdrawal, and often present as symmetric parkinsonism, dystonia, dyskinesias, or cerebellar toxicity. In contrast, disease-related movement disorders usually have an insidious course, asymmetric or mixed phenotypes, and are associated with active malignancy. Neuroimaging supports this distinction, with characteristic toxic patterns favoring drug effects and structural or infiltrative lesions indicating disease-related mechanisms.

### Diagnostic Implications

The diagnostic implications of these pathophysiologic differences are substantial. The strong association between lymphoid malignancies and cerebellar syndromes highlights the need for early MRI with high-resolution cerebellar sequences, CSF analysis, and comprehensive paraneoplastic antibody testing. Because paraneoplastic cerebellar degeneration may precede the detection of lymphoma, neurologists should maintain a low threshold to investigate occult hematologic disease when encountering unexplained subacute ataxia [20].

In myeloid neoplasms, the predominance of choreiform syndromes indicates that diagnostic evaluation should prioritize vascular imaging, serum viscosity indices, and inflammatory markers, rather than relying solely on CSF or MRI for direct infiltration. Recognition of drug-induced cerebellar or basal ganglia toxicity also necessitates careful review of chemotherapy regimens, particularly cytarabine, methotrexate, and tyrosine kinase inhibitors [21,22].

Plasma-cell neoplasms require an integrated approach, as neuroimaging may reveal white matter changes, cerebellar atrophy, or basal ganglia signal abnormalities. Because amyloid and immune-mediated processes often coexist, CSF biomarkers, serum free light chains, PET-CT, and electrophysiology may all contribute critical information. The review demonstrates that a single diagnostic modality is insufficient, and optimal evaluation requires multimodal correlation tailored to the hematologic subtype [19,23].

### Treatment Implications

Treatment strategies differed significantly across malignancy groups due to their distinct mechanisms. In lymphoid neoplasms, management often required simultaneous tumor-directed therapy and immunotherapy, especially when autoimmune cerebellar degeneration or anti-neuronal antibodies were present. Rituximab-based regimens, IVIG, corticosteroids, and plasmapheresis demonstrated favorable outcomes when initiated early. The marked neurological improvement underscores the importance of prompt recognition [24,25].

For myeloid malignancies, addressing the systemic drivers of movement disorders was critical. Therapies that reduced blood viscosity, controlled leukocytosis, or modified cytokine loads provided meaningful neurological improvement. Drug-induced syndromes often resolved with discontinuation or dose adjustment of causative agents. These findings highlight the need for vigilance when monitoring patients receiving high-dose cytarabine or targeted therapies [26].

In plasma-cell neoplasms, management often required a combination of myeloma-directed therapy, treatment of immune dysregulation, and symptomatic management of parkinsonism or cerebellar dysfunction. Dopaminergic agents were variably effective, depending on the mechanism. Cases with amyloid-associated movement disorders required systemic management of AL amyloidosis in addition to neurological care [19].

Across hematological malignancies, movement disorders showed mechanism-specific clinical patterns, with onset and phenotype reflecting direct central nervous system involvement, immune-mediated processes, systemic effects, or treatment exposure; neurological outcomes were often favorable when the underlying hematologic disease and pathophysiology were promptly addressed. In our review, treatment of movement disorders was primarily directed at the underlying mechanism. Cases due to direct CNS involvement improved mainly with tumor-directed therapy. Paraneoplastic syndromes often had limited neurological recovery despite immunotherapy. In myeloproliferative neoplasms such as polycythemia vera, hyperkinetic movements frequently improved after correction of hyperviscosity with hematologic treatment. Drug-related cases improved after withdrawal of the offending agent. Parkinsonism was uncommon in myeloid neoplasms, and levodopa benefit was not consistently documented, suggesting a limited role compared with treating the primary hematologic cause.

This review has several limitations. Most available evidence comes from isolated case reports and small series, creating inherent publication and reporting biases. Clinical details, investigations, and outcomes were inconsistently documented, limiting uniform comparisons. Pathogenic attribution often relied on author interpretation rather than standardized criteria. The lack of controlled studies prevents estimation of true frequencies or causal relationships. Diagnostic workups, imaging protocols, and treatment approaches varied widely across reports and time periods, reducing generalizability. Follow-up information was frequently incomplete, making long-term outcomes difficult to assess. A further limitation relates to treatment-related movement disorders. Although cases associated with immunotherapies, including immune checkpoint inhibitors, monoclonal antibodies, and chimeric antigen receptor T-cell therapy, were classified under the treatment-related mechanism category, they were not analyzed as a distinct quantitative subgroup across malignancy types.

## Conclusion

In conclusion, movement disorders in blood cancers follow distinct, predictable patterns. Lymphoid tumors often cause cerebellar and immune-mediated syndromes, myeloid neoplasms typically produce systemic-driven hyperkinetic disorders, and plasma-cell neoplasms commonly lead to parkinsonian features. Lymphoid cancers frequently infiltrate the CNS directly, while myeloid and plasma-cell diseases usually act through indirect metabolic, immune, vascular, infectious, or treatment-related pathways. Recognizing these group-specific patterns enables faster diagnosis and more targeted management.

## Use of Artificial Intelligence (AI) Software

ChatGPT 5 was used to correct English and Grammar. During the preparation of this work the authors used ChatGPT 5 in order to correct English and Grammar along with data analysis. After using this tool/service, the authors reviewed and edited the content as needed and take full responsibility for the content of the published article.

## Data Accessibility Statement

All data generated or analysed during the preparation of this review are contained within the article and its accompanying supplementary materials.

## Additional File

The additional file for this article can be found as follows:

10.5334/tohm.1147.s1 Supplementary File. Supplementary Tables 1 to 4.

## Financial Disclosures

RKG has received honoraria and royalties for writing clinical summaries and update articles for MedLink Neurology and UpToDate. Other authors declare that there are no additional disclosures to report.
