# Supplementary material for: Spectrum of Movement Disorders in Hematological Malignancies: A Comprehensive Systematic Review of Clinical Phenotypes, Mechanisms, and Outcomes
Source: Tremor Other Hyperkinet Mov (N Y). 2026 Mar 19;16:18. doi: 10.5334/tohm.1147 (PMC13004057; doi:10.5334/tohm.1147)
Supplement: Supplementary File. — Supplementary Tables 1 to 4. [file tohm-16-1-1147-s1.zip › tohm-1147_garg-s1/Supplementary Table 1.docx]

**Supplementary Table 1: Structured Dataset Summarizing Demographics, Hematological Features, CNS Involvement, Movement Disorder Phenomenology, Ancillary Findings, Mechanisms, and Outcomes in 152 Reported Cases of Lymphoid Malignancies**

| **Author/Year** | **Country** | **Age/Sex** | **Hematological Category** | **Specific Diagnosis** | **Disease Stage** | **Molecular Markers** | **CNS Involvement (Evidence)** | **Movement Disorder Type/ Ataxia** | **Phenomenology** | **Timing of Onset** | **Associated Neurologic Manifestations** | **Paraneoplastic Antibodies** | **Drug-Induced Neurotoxicity** | **CSF Findings** | **Neuroimaging Findings** | **PET-CT Findings** | **Hematology Treatment** | **Targeted Therapy** | **HSCT** | **Neurology Treatment** | **Neurological Outcome** | **Hematological Outcome** | **Proposed Mechanism** | **Follow-up Duration** |
| --- | --- | --- | --- | --- | --- | --- | --- | --- | --- | --- | --- | --- | --- | --- | --- | --- | --- | --- | --- | --- | --- | --- | --- | --- |
| Yawata G et al., 2025 | Japan | 52/F | Non-Hodgkin Lymphoma (T-cell) | ALK-negative Anaplastic Large Cell Lymphoma | Stage III; IPI Low-Intermediate | CD30+, CD3+, ALK– | No malignant cells in CSF; cerebellar atrophy on MRI; anti-Tr/DNER antibody positive | Cerebellar ataxia | Gait ataxia, limb ataxia, dysarthria, saccadic pursuit, mild cognitive impairment | Subacute progression over 3 months | Vertigo, writing difficulty, mild cognitive impairment | Anti-Tr/DNER antibody (2+); others negative | None reported | WBC 2/µL, Protein 66 mg/dL, IgG index 0.44, OCB positive (6 bands), no malignant cells | Mild left cerebellar atrophy | FDG uptake in right inguinal lymph node and sternum | BV-CHP regimen | Brentuximab vedotin | Not performed | No immunotherapy; tumor-directed therapy only | Persistent ataxia; no recovery | Complete remission | Immune-mediated cerebellar degeneration via anti-DNER IgG1 | 2 years |
| Wadhera S et al., 2025 | India | 32/M | Lymphoproliferative Disorder | Lymphomatoid Granulomatosis (EBV-associated), Grade 1 | Multisystem involvement (CNS + Lung) | CD3+ small lymphocytes; CD20+, CD30+ atypical cells; EBER-ISH positive | MRI T2/FLAIR lesions with peripheral enhancement; multiple cranial nerve palsies (III, V, VI, VII, IX) | Cerebellar ataxia | Broad-based gait, scanning speech, action tremor, MMSE 20/30, cranial nerve deficits | Symptom evolution over 8 months | Cranial nerve involvement, cognitive impairment, ataxia, tremor, dysarthria | EBER-ISH positive | Interferon-induced drug rash (CTCAE grade 3) | Not reported | Multiple T2/FLAIR hyperintense lesions in deep white matter, brainstem, cerebellum, MCPs | HRCT chest: multiple bilateral nodules | Interferon alpha-2b → switched to R-CHOP | Rituximab (part of R-CHOP) | Not performed | R-CHOP after interferon intolerance | Neurologic improvement | Radiologic and symptomatic improvement | EBV-driven angiocentric lymphoproliferation involving CNS + lungs | Not reported |
| Venkatesan A et al., 2025 | USA | 69/M | Hematological malignancy (DLBCL) with CNS relapse history | Diffuse Large B-Cell Lymphoma (EBV+, prior CNS relapse) but final diagnosis Powassan virus encephalitis | Not staged; recurrent CNS disease considered then excluded | Prior EBV+ DLBCL; post-bendamustine+rituximab; CAR‑T; ibrutinib | CSF pleocytosis (21 cells/µL), PCR positive for Powassan virus lineage II; cerebellar & brainstem FLAIR hyperintensity | Cerebellar ataxia | Limb & truncal ataxia, nystagmus, hypophonia, diplopia, CN VI palsies, dyscoordination | 2–3 week rapid progression | Cranial nerve palsies, fasciculations, dysphagia, lethargy | None relevant | None reported | 21 WBC/µL (77% lymphocytes), protein 74 mg/dL, glucose 51 mg/dL, atypical activated lymphocytes | Bilateral cerebellar cortical & pontine FLAIR hyperintensity; unchanged calcified lesion in left frontal lobe | No PET-CT performed | Past therapy: bendamustine+rituximab, CAR‑T, ibrutinib | None active currently | Not performed | Acyclovir, ceftriaxone, vancomycin, ampicillin, IVIG, dexamethasone | Partial recovery then progressive decline | DLBCL in remission; no relapse | Tick‑borne Powassan virus–mediated cerebellitis/rhombencephalitis | 6 months (death) |
| Vasey et al., 2025 | USA | 47/F | Lymphoid malignancy | Acute lymphoblastic leukemia (remission) + hypoplastic low-grade MDS | Not staged | None reported | No CNS involvement; MRI/CT/CTA normal | Orofacial dyskinesia, limb dyskinesia, dystonia | Facial grimacing, limb dance-like movements, dystonia, expressive aphasia | Acute onset (~30 min) | Aphasia, ataxia, dystonia | None reported | Yes—methylphenidate-induced neurotoxicity | Not performed | MRI/CT/CTA normal | Not done | Cyclosporine; supportive hematology care | None | No | Lorazepam; benztropine; discontinue methylphenidate | Near-complete resolution | Stable remission | Dopaminergic hypersensitivity | Short-term follow-up |
| Tilley et al., 2025 | Canada | 86/F | Chronic lymphocytic leukemia | Longstanding CLL (15 yrs), no Richter transformation | Not staged; widespread adenopathy + splenomegaly | Flow cytometry: atypical lymphocytes = non-transformed CLL | No CNS infiltration; JCV PML | Cerebellar ataxia | Gait instability, dysarthria, diplopia, dysmetria, nystagmus | 4 weeks (subacute) | Diplopia, inattention, dysarthria, dysdiadochokinesia | All paraneoplastic antibodies negative | None | Inflammatory CSF; JCV PCR positive | MRI: bilateral MCP + pontine hyperintensities; CT normal | PET: non-avid lymph nodes; no CNS lymphoma | No CLL therapy (avoid immunosuppression) | None | No | IV methylprednisolone; IVIG aborted due to reaction | Progressive deterioration → palliative care | Stable CLL without transformation | JC virus–mediated PML | ~7 weeks |
| Kadubandi A et al., 2025 | USA | 49/M | Hodgkin Lymphoma (Classical HL) | Classical Hodgkin Lymphoma (CD30+, CD15+, dim PAX5) | Ann Arbor Stage IIA | Anti-Tr (DNER) positive; GAD positive; CD15+, CD30+, dim PAX5 | CSF pleocytosis; elevated protein; oligoclonal bands; MRI initially normal; PET-positive nodes | Cerebellar ataxia | Ataxia, vertigo, dysarthria, gait imbalance, nystagmus, dysphagia needing G-tube | 4-week subacute onset | Dysarthria, diplopia, dysphagia, vertigo, gait ataxia | Anti-Tr (CSF 1:128); GAD antibody (serum) | None reported | CSF WBC 159→40 (lymphocytic), high protein, OCB+, negative cytology | MRI normal early; persistent symptoms later | PET: periaortic & aortocaval nodes SUV 5.45 → reappeared, biopsy proven HL | ABVD ×2 → AVD + Brentuximab Vedotin ×2 | Brentuximab Vedotin | Not performed | Steroids, IVIG, plasmapheresis, rituximab | Severe persistent cerebellar syndrome | Complete remission | Anti-Tr mediated Purkinje cell damage | 2 years |
| Iguchi T et al., 2025 | Japan | 75/M | Primary CNS Lymphoma (Lymphomatosis cerebri subtype) | Diffuse Large B-Cell Lymphoma (DLBCL) presenting as Lymphomatosis cerebri | Brain-only PCNSL; no systemic staging | CD20+ large atypical lymphoid cells on biopsy | Diffuse T2/FLAIR hyperintensities in deep WM, corpus callosum, basal ganglia, thalami, midbrain; mild CSF pleocytosis | Parkinsonism + Holmes tremor | Cognitive decline, bradykinesia, hypomimia, gaze paresis, severe gait instability, retropulsion, Holmes tremor | 6-month progressive course with rapid decline in last month | Severe cognitive impairment, hypophonia, hypomimia, gaze limitation, tremor, postural instability | None | None reported | Mild pleocytosis, elevated protein, cytology negative; no flow cytometry | Diffuse non-enhancing T2/FLAIR hyperintensities; mild enhancing frontal nodule | No PET-CT reported | Whole-brain radiation therapy | None | Not performed | Supportive therapy + radiation | Persistent disability; transferred to long-term care | Primary CNS lymphoma only | Tumor infiltration of critical motor pathways | Not clearly stated |
| Adibi A et al., 2025 | Iran | 14/M | Hodgkin Lymphoma (survivor) | Autoimmune cerebellar ataxia with anti-Tr/DNER positivity | No active lymphoma; recurrence excluded | Anti-Tr/DNER antibody positive | Cerebellar ataxia with MRI cerebellar atrophy | Cerebellar ataxia | Truncal + limb ataxia, dysarthria, nystagmus, tremor, dysmetria, hyperreflexia | 1-month progressive course | No sensory loss, no diplopia, no vertigo | Anti-Tr/DNER antibody positive | None reported | CSF not reported | MRI: cerebellar atrophy (vermis predominant) | CT: no HL recurrence | Previously treated HL; no current therapy | None | Not performed | IVIG → plasmapheresis → monthly IVIG × 6 months | Marked neurological recovery | No HL recurrence | Anti-Tr/DNER-mediated Purkinje cell injury | 18 months |
| Silva L et al., 2024 | Portugal | 35/F | T-cell lymphoma (intestinal T-cell lymphoma) | Monomorphic epitheliotropic intestinal T-cell lymphoma | Refractory; early progression after multiple regimens + auto/allo HSCT | Not applicable (immune-mediated) | CSF pleocytosis (24 cells), ↑protein, MRI normal, EEG mild slowing | Parkinsonism | Hypomimia, psychomotor slowing, bilateral tremor, rigidity, global hypokinesia | 1 week after fever onset; 2 weeks after pembrolizumab | Fever, mild tremor; no seizures or cognitive decline | None | None reported | CSF: 24 WBC, protein 0.50 g/dL, normal glucose, no OCB, antibodies negative | MRI normal; EEG mild slowing | CT: no infection or malignancy | Prior chemotherapy + autologous + allogeneic HSCT; on pembrolizumab | Pembrolizumab | Autologous + allogeneic HSCT previously done | IV methylprednisolone → oral taper; IVIG | Full resolution; mild tremor on rechallenge | Underlying lymphoma refractory but unchanged | PD‑1 inhibitor–mediated dopaminergic pathway dysfunction | 6 months on pembrolizumab without recurrence |
| Peter et al., 2024 | France | 50/M | Hodgkin lymphoma (NLPHL) | Anti-RGS8 paraneoplastic cerebellar ataxia | Stage I | IgG1 anti‑RGS8 | Purkinje cell involvement | Cerebellar ataxia | Dysarthria, nystagmus, diplopia, severe gait ataxia | 8 weeks | Brisk reflexes, diplopia | Anti‑RGS8 | None | Clear CSF, OCBs | Normal | Axillary hypermetabolic nodes | IVIG, steroids, rituximab, R‑CHOP | None | No | IVIG + steroids | Severely disabled | Complete remission | RGS8-mediated Purkinje injury | 10 months |
|  |  | 50/M | Hodgkin lymphoma (NLPHL) | RGS8-associated ACA | Stage III | IgG1/IgG2/IgG4 anti‑RGS8 (1:256,000) | Pure cerebellar involvement | Mild gait ataxia, intention tremor | Oscillopsia, shimmering lights, nystagmus, dysmetria | 6 months | None | Anti‑RGS8 | None | Inflammatory CSF, OCBs | Normal | Hypermetabolic nodes | R‑CHOP + IT‑MTX | None | No | IVIG | Mild persistent disability | Complete remission | Purkinje autoimmunity (RGS8) | 3 years |
|  |  | 68/M | Possible B‑cell lymphoma (not found) | RGS8-associated ACA | Not applicable | Anti‑RGS8 (PhIP‑seq + CBA) | Severe cerebellar ataxia + ocular signs | Severe gait ataxia | Oscillopsia, diplopia | 4 months | Tremor, ocular issues | Anti‑RGS8 | None | Lymphocytic pleocytosis, high protein, OCBs | Normal | No tumor on multiple PET scans | Steroids, IVIG, rituximab, cyclophosphamide, MMF | None | No | Immunotherapy | Severe disability | No malignancy diagnosed | RGS8-mediated Purkinje autoimmunity | 5 years |
| Handzic A et al., 2024 | Canada | 61/F | Low-grade B-cell lymphoma (extranodal marginal zone) | Low-grade B-cell lymphoma with anti-Ma2 paraneoplastic syndrome | Untreated for 5 years; new pulmonary nodule | CD20+, PAX5+, BCL2+; CD10–, BCL6–, cyclin D1–; MIB-1 ~30% | Longitudinally extensive central cord hyperintensity; high CSF protein; anti-Ma2 strongly positive | Cerebellar degeneration | Downbeat nystagmus → macrosaccadic oscillations; oscillopsia; ataxia; severe allodynia | Subacute (2 months) | Weakness (C5–C7), sensory loss, truncal allodynia | Anti-Ma2 (very high titer) | None reported | CSF protein 790 mg/L; cytology/flow negative; anti-Ma2 positive | Longitudinal medulla→T11 hyperintensity; mild enhancement | PET initially negative; follow-up PET detected lung lymphoma | R-CVP type regimen (rituximab, cyclophosphamide, vincristine, prednisone) | Rituximab | Not performed | IV methylprednisolone x5 days, IVIG, paravertebral block | Persistent deficits; partial response | Lymphoma confirmed; on therapy | Anti-Ma2–mediated neuronal injury | 2 months |
| Donaghy et al., 2024 One out of 4 had movement disorder. | USA | 44/F | AML | Acute myeloid leukemia | Active leukemia | Not reported | Later CNS involvement (unrelated to MTX) | Myoclonus | Encephalopathy, aphasia, myoclonus | 9 days after IT MTX | Aphasia | Not applicable | Yes—MTX toxicity | WBC 3, protein 48, glucose 44; cytology negative | Subcortical DWI+ + T2-FLAIR lesions | Not done | Cytarabine | None | No | Dextromethorphan | Recovered; later CNS leukemia | Progression later | MTX NMDA excitotoxicity | Not specified |
| Arora et al., 2024 | India | 63/F | Chronic lymphocytic leukemia | CLL diagnosed 1 year prior; splenomegaly + lymphadenopathy | Not staged | FISH abnormality associated with NHL-type changes | Suspected CNS involvement; MRI normal; CSF lymphocytic pleocytosis | Ocular flutter + intermittent jaw movements | Horizontal ocular flutter, jaw movements, tremors, altered sensorium | Acute–subacute over days | Altered sensorium, tremors, rigidity | Negative (Hu, Yo, Ri, PNMA2, CV2, amphiphysin) | Unlikely | Protein 64 mg/dL↑, TLC 50 (lymphocytic), glucose normal; all cultures/PCR negative | MRI brain & spine normal | Not performed | Rituximab + bendamustine (prior) | Rituximab | No | Levetiracetam, acyclovir, carbapenem, amphotericin B | Died (cardiorespiratory arrest) | Poor (immunosuppressed) | Possible CLL-related or immune-mediated brainstem dysfunction | Short hospital course |
| Yokota Y et al., 2023 | Japan | 73/F | Primary CNS lymphoma | PCNS-DLBCL | Localized CNS-only, no systemic involvement | CD20+, CD79a+, Ki-67 90%, NR1 negative | MRI/biopsy confirmed CNS lymphoma | Truncal/cervical dystonia | Reduced speech, agitation, dystonia, cognitive decline | Acute onset | Cognitive dysfunction, agitation, abnormal EEG | Anti-NMDAR IgG positive (CSF only) | None | Pleocytosis, low protein, normal glucose, OCB positive | FLAIR lesions frontal/temporal, Gd-enhancing mass, ↑Cho/Cr | Not done | Tumor resection + radiotherapy (30 Gy) | None | None | IV dexamethasone, supportive | Mild persistent cognitive dysfunction | Tumor reduced, no systemic disease | Paraneoplastic NMDARE from tumor-induced antigen exposure | 4 months |
| Varela et al., 2023 | Argentina | 80/F | Chronic lymphocytic leukemia (CLL) | CLL treated with rituximab | Not staged | Not reported | Yes – JC virus positive (PML) | Cerebellar ataxia | Vertigo, gait ataxia, dysarthria, dysmetria, nystagmus | 5 months after rituximab | Nystagmus, dysarthria, limb dysmetria | Not tested | No | CSF: JCV DNA positive | MRI: cerebellar T2 hyperintensity; dentate nucleus spared (shrimp sign) | Not done | Rituximab (prior) | Rituximab | No | Supportive; rituximab discontinued | Stable after 1 year | Stable CLL | JC virus–mediated cerebellar PML | 1 year |
| Samaha & Larner, 2023 | UK | 35/M | Lymphoid malignancy | Hodgkin lymphoma with anti-Tr paraneoplastic cerebellar degeneration | Localised right-groin lymph node involvement | Anti-Tr antibody positive; Anti-Yo/Hu/Ri/Ma negative | Subacute cerebellar syndrome; CSF lymphocytosis; normal initial MRI | Cerebellar ataxia | Gait ataxia, dysarthria, gaze-evoked nystagmus, oscillopsia | Subacute onset (3 weeks) | Dysarthria, nystagmus, dysdiadochokinesia, ankle clonus | Anti-Tr antibody positive | None | CSF: WCC 70→17 lymphocytes; protein 0.58→0.55 g/L; OCB negative | Initial MRI normal; 4-year MRI marked cerebellar atrophy | PET-CT: isolated right-groin Hodgkin lymphoma | ABVD x2 chemotherapy | None | None | Supportive neuro-rehabilitation | Persistent gait ataxia, recurrent falls | Good oncologic response; discharged from oncology after 2 years | Paraneoplastic anti-Tr (DNER) cerebellar degeneration | 4 years |
| Mesbah-Oskui et al., 2023 | Canada | 65/M | Clonal CD8-positive T-cell lymphoproliferative disorder (unclassifiable T-LPD) | CNS-infiltrating CD8-positive T-cell LPD, EBV-negative | Not meeting WHO lymphoma criteria; marrow + CSF + CNS infiltrated | CD8+, TCR-βF1+, TIA-1+, CD57(subset+); CD4–, CD56–; Ki-67~0%; EBER– | Diffuse confluent T2 WM hyperintensities; CSF clonal T-cells; marrow infiltrated | Truncal ataxia | Cognitive decline → neuropsychiatric symptoms → truncal ataxia → falls | 1.5 years cognitive decline + 3 months ataxia | Restricted upgaze, cognitive & behavioral decline | None detected | None | CSF: 36 WBC (lymphocytic), protein 0.85 g/L, TCR clonality+, infectious/PNS panel– | MRI: diffuse progressive T2 WM hyperintensities, no enhancement | PET: no hypermetabolic lesions | CHOP ×1 → HD methotrexate + temozolomide ×3 | None | None | Steroid pulse → transient improvement; maintained on prednisone | Progressive; died of aspiration | Not applicable (no systemic lymphoma) | CD8 clonal infiltration with low proliferative but aggressive behavior | 2.5 years from onset; 1 year from presentation |
| Franzini et al., 2023 | Italy | 60/M | Lymphoid malignancy | Primary CNS B-cell lymphoma (PCNSL) – cerebellar | Localised posterior fossa lesion with mass effect | None reported | Right cerebellar intra-axial mass; biopsy confirmed lymphoma | Cerebellar ataxia | Wide-based gait, dysarthria, dysmetria, instability, mental slowing | Subacute onset | Ataxia, dysmetria, gait instability, raised ICP signs | None | None | Not described | T1 iso/hypointense; T2 hyperintense; strong central enhancement; CT hypodense | None | Fluorescein-guided partial resection of enhancing tumor | None | None | Not specified | Full neurological recovery | Referred for adjuvant therapy | Lymphoma infiltration; BBB disruption | Not stated |
| Fakhari et al., 2023 | Iran | 66/M | Lymphoid malignancy | Gray zone lymphoma (GZL) | Mediastinal lymphadenopathy; systemic symptoms for 1 year | CD30+, PAX5+, CD20+, CD3 background+, CD15– | Paraneoplastic SPS; no direct CNS involvement | Stiff-person syndrome | Painful spasms, stiffness, bradykinesia, encephalopathy | Subacute onset | Encephalopathy, gaze palsy, MMSE 18, anemia | Amphiphysin 3+; GAD65 negative | None | Normal CSF (WBC 0, RBC 0, protein 45 mg/dL, glucose 58) | Brain MRI normal; HRCT mediastinal nodes | Bone scan rib uptake (nonspecific) | No lymphoma treatment initiated (diagnosis delayed) | None | None | Baclofen, Clobazam, IVIG, Levetiracetam | Death before lymphoma therapy | No hematologic treatment | Paraneoplastic amphiphysin‑mediated SPS revealing GZL | ~1 year symptoms; final brief admission |
| Algahtani et al., 2023 | Saudi Arabia | 73/M | Lymphoid malignancy | Follicular lymphoma WHO grade 2, stage II | Stage II; para-aortic and iliac lymphadenopathy | BCL2+, CD10+, follicular architecture; marrow negative | No CNS lymphoma; neurological symptoms due to iron overload | Tremor (action/postural) | Bilateral coarse postural tremor; handwriting difficulty | Chronic (2 yrs); worsened over last 4 months | Basal ganglia/substantia nigra iron deposition | None | None | Ferritin 1411 µg/L; transferrin saturation 57% | SWI MRI: bilateral basal ganglia & SN blooming | CT abdomen/pelvis: para-aortic nodes, hepatomegaly | Watch-and-wait for lymphoma | None | None | Propranolol, gabapentin ineffective; primidone improved tremor | Stable, no chemo started | Watch and wait; marrow negative | Iron overload + incidental lymphoma | 2-year course with recent worsening |
| Kirkedal et al., 2022 | Denmark | 73/F | Lymphoid malignancy | Primary CNS lymphoma (DLBCL) | CNS-only disease; left basal ganglia/corpus callosum | Malignant B-cells in CSF; marrow negative | Direct CNS involvement (basal ganglia) | Parkinsonism | Right-sided bradykinesia, rigidity, hypophonia, oligomimia, micrographia | Subacute (3 weeks) | Gait imbalance, fine motor difficulty, micrographia | None | None | CSF: malignant B-cells; mild hyponatremia | MRI: enhancing left basal ganglia lesion, edema, diffusion restriction | PET/CT: no systemic malignancy | Rituximab + HD-MTX + vincristine + procarbazine | None | None | Rehabilitation | Significant recovery; mild residual bradykinesia | Excellent response; nearly complete regression on 9-month MRI | Basal ganglia invasion disrupting motor circuits | 9 months |
| Fiorelli et al., 2022 | Italy | 38/M | Lymphoid malignancy | Classical Hodgkin lymphoma (nodular sclerosis), stage III | Stage III with supraclavicular lymphadenopathy | RS cells: PAX5-, CD15+, CD30+, LCA-, EBV-, DNER+ | Paraneoplastic cerebellar degeneration; no CNS tumor | Diplopia and nystagmus | Gaze-evoked diplopia, spontaneous diplopia, right-beat nystagmus, CN VII deficit | Subacute | Diplopia, nystagmus, CN VII palsy, frontal pain | Anti-DNER antibodies positive | None | CSF pleocytosis (108 cells/mm3), no OCB, minimal non-clonal B cells | MRI normal initially → later cerebellar atrophy | No pathological findings described | ABVD x 6 cycles | None | None | Supportive care | Marked improvement; mild residual diplopia | Complete remission | Paraneoplastic anti-DNER cerebellar degeneration | 9 months |
| Feng et al., 2022 | China | 67/M | Lymphoid malignancy | Diffuse large B-cell lymphoma (gastric, stage III) | Gastric antrum ulcer + lymphadenopathy (stage III) | CD20+++, CD79α++, CD10+, BCL-6++, P53+, Ki-67 90%, MUM-1+, BCL-2− | No CNS lymphoma; Wernicke encephalopathy | Ataxia | Ataxia, metamorphopsia, dysdiadochokinesia, ocular motility deficit | Subacute (50 days) | Cognitive dysfunction, memory impairment, acalculia | All neuronal antibodies negative | None | CSF normal (WBC 0, RBC 0, normal protein & glucose) | MRI: extensive cortical + callosal + pontine lesions; T2/FLAIR hyperintense | CT abdomen: gastric ulcer + lymph nodes | No chemotherapy (refused) | None | None | IM thiamine + IV dexamethasone | Later deterioration after discharge | No lymphoma treatment | Thiamine deficiency from malignancy-related malabsorption | 50-day onset + months follow-up |
| Ahn et al., 2022 | South Korea | 71/F | Lymphoid malignancy | Primary CNS lymphoma (DLBCL) | Diffuse corticospinal tract infiltration (lymphomatosis cerebri) | CD20+, CD3-, MUM1+, Ki-67 90% | Direct CNS infiltration | Ataxic quadriparesis | Quadriparesis, dysarthria, dysphagia, ataxia | Subacute (2–3 months) | Gait instability, recurrent falls, UMN signs | None | None | CSF cytology normal | MRI: wine-glass sign, bilateral CST hyperintensities, no enhancement | CT chest/abdomen: no systemic malignancy | High-dose steroids + chemotherapy | None | None | Steroid pulse therapy | Death 2 months later | No systemic lymphoma | Diffuse CNS infiltration | Several months |
| Storti et al., 2021 | Italy | 71/M | Lymphoid malignancy | Waldenström’s macroglobulinemia | Bone marrow involvement; IgM κ monoclonal gammopathy | ANA 1:320; IgM κ monoclonal gammopathy | No CNS lesion; WM-related palatal–lingual tremor | Palatal–lingual tremor | 4 Hz rhythmic contractions of palate & tongue; shaky speech | 1-year progressive course | EMG 4 Hz synchronous activity; blink-reflex abnormalities | No classical paraneoplastic antibodies | None | CSF normal; MRI normal | MRI normal | Not reported | Modified R-CHOP x 6 cycles | None | None | Supportive neurologic care | Significant recovery with residual tremor | Hematologic remission | WM-induced immune hyperexcitability | 1 year + 8 months follow-up |
| Okano et al., 2021 | Japan | 75/M | Lymphoid malignancy | Primary CNS lymphoma (DLBCL) | Left thalamus + basal ganglia + periventricular WM | CD20 strongly positive | Direct CNS involvement (BG + thalamus + WM) | Parkinsonism | Bradykinesia, tremor, gait disturbance, micrographia | Subacute (1 month) | Gait disorder, postural instability, masked face | None | None | ↑ IL-2 receptor; normal LDH; CSF nondiagnostic | MRI: hyperintense BG/thalamus/WM lesions with enhancement | CT: thalamic hyperdensity + periventricular hypodensity | Corticosteroids + R-MPV chemo | None | None | Corticosteroid pulse | Full resolution | Complete response at 8 months | Tumor infiltration disrupting dopaminergic circuits | 8-month follow-up |
| Nothrop et al., 2021 | Australia | 81/F | Lymphoid malignancy | SMZL transforming to DLBCL | Bone marrow transformation; PET negative for nodes | CD5 partial, CD10-, CD19+, CD20 partial, CD23-, lambda-restricted clone | No CNS lymphoma; paraneoplastic chorea | Chorea | Left > right chorea, ballism, mild dysarthria, sensory neuropathy | Subacute (2 weeks worsening) | Stocking sensory loss, dysarthria | All paraneoplastic antibodies negative | None | CSF: OCB positive; normal studies | MRI brain & spine normal | PET: splenic uptake; no lymphadenopathy | Palliative chemotherapy | None | None | Clonazepam 0.5 mg BID | Complete resolution | Remission after treatment | Paraneoplastic basal ganglia dysfunction | Months |
| Nanda et al., 2021 | India | 37/F | Lymphoid malignancy | Diffuse large B-cell lymphoma (germinal center type) | Right adnexal mass + appendiceal lesion; supra- & infradiaphragmatic nodes | CD20 diffuse strong positivity | No CNS lymphoma; paraneoplastic cerebellar degeneration | Cerebellar ataxia | Gait instability, diplopia, nystagmus, staccato speech | Subacute (1 month) | Diplopia, vertigo, speech changes | Paraneoplastic antibodies negative | None | CSF normal; no malignant cells | MRI brain normal | PET-CT: widespread nodal disease | IV methylprednisolone + chemotherapy | None | None | Steroids + supportive care | Partial recovery | Active lymphoma; early stage of treatment | Purkinje cell immune attack | 2 weeks |
| Martínez-Burbano et al., 2021 | Ecuador | 72/M | Lymphoid malignancy | Non-Hodgkin lymphoma (T-cell phenotype) | Large anterior mediastinal mass + pulmonary nodules | T-cell immunophenotype on biopsy | No CNS involvement; paraneoplastic SPS | Stiff-person syndrome | Painful spasms, rigidity, hyperextension posture, pseudo-spastic gait | Subacute (2 months) | Falls, pruritus, anxiety, OCD-like behaviors | Anti-GAD65 strongly positive | None | CSF normal except very high anti-GAD65 | MRI not reported; EMG SPS pattern | PET-CT: hypermetabolic mediastinal mass | R-CHOP x 6 + radiotherapy | None | None | Baclofen, clonazepam, valproate, IV steroids, IVIG | Complete neurological remission | Complete metabolic response | Anti-GAD65 GABAergic dysfunction | 6 months |
| Makranz et al., 2021 (One in 5 cases had movement disorder) | Israel | 78/F | Lymphoid malignancy | Diffuse large B-cell lymphoma (DLBCL) | Brain parenchymal involvement | CD20+, Ki-67 high | Direct CNS involvement on biopsy | Parkinsonism | Bradykinesia, gait freezing, masked facies, rigidity | Subacute–chronic | Falls, cognitive decline, gait difficulty | Not assessed | None | CSF nondiagnostic; no malignant cells | MRI: diffuse T2/FLAIR hyperintensities; frontal mass biopsied | PET/CT: frontal hypermetabolic lesion; no systemic disease | High-dose methotrexate–based chemotherapy | None | None | Levodopa ineffective; supportive rehab | Poor neurological outcome | Partial oncologic response | Direct lymphoma infiltration of basal ganglia/cognitive circuits | Months |
| Kitamura et al., 2021 | Japan | 72/M | Lymphoid malignancy | Peripheral T-cell lymphoma (T follicular helper phenotype) | Generalised nodal disease (multiple lymph node groups) | CD3+, CD4+, CD5+, CD7+, CD30+, BCL6+, CXCL13+ | No CNS lymphoma; SPECT left striatal hypoperfusion | Hemichorea | Right-sided chorea + personality change | Subacute onset during treatment | Personality changes; no seizures | All paraneoplastic antibodies negative | None | CSF mild pleocytosis (18/µL), protein 78 mg/dL; cytology negative | MRI normal; SPECT: left striatal hypoperfusion | PET-CT: widespread FDG-avid lymphadenopathy | DA-EPOCH ×6 → CR; Salvage HD-MTX + cytarabine ×4 | None | None | No specific movement therapy | Complete neurological recovery | Hematologic improvement | Striatal hypoperfusion due to lymphoma | Several months |
| Khera et al., 2021 | India | 6/M | Hematologic malignancy | B-lineage ALL | On BFM-2002 protocol; interim maintenance | MRD negative; no markers reported | No CNS invasion; MTX toxic leukoencephalopathy | Choreoathetosis | Generalised choreoathetoid movements | Subacute (10 days post-MTX) | Seizures, irritability, altered sensorium | Not assessed | MTX toxicity | CSF normal; no blasts | MRI: scattered periventricular white-matter hyperintensities | CT normal | High-dose MTX IV + intrathecal MTX | None | None | Trihexyphenidyl, clonazepam, levetiracetam | Good neurological recovery | ALL in remission course | Toxic leukoencephalopathy | Multiple cycles; no recurrence |
| Kajtazi et al., 2021 | Saudi Arabia | 53/F | Lymphoid malignancy | Classical Hodgkin lymphoma (mixed cellularity) | Oropharyngeal mass + bilateral cervical level II lymphadenopathy | CD30+, CD15+, PAX5+, EBER+, CD45−, CD20−, CD2−, CD23−, CD57−, BCL6− | No CNS lymphoma; paraneoplastic cerebellar degeneration | Cerebellar ataxia | Severe gait ataxia, dysarthria, nystagmus, diplopia, head tremor, dysmetria | Subacute–chronic (5 months) | Dysarthria, diplopia, severe head tremor | Antibodies Hu/Yo/Ri/Tr/VGCC negative | None | CSF: WBC 325 (98% lymphocytes), protein ↑, cytology negative | MRI brain & spine normal | PET-CT: FDG-avid cervical nodes (SUV 9.4) | ABVD x6 + 30 Gy radiotherapy | None | None | Supportive rehab; levothyroxine | Mild residual ataxia | Complete remission | Paraneoplastic pancerebellar degeneration | 5 years |
| Joshi et al., 2021 – Patient 2 | Australia | 56/F | Hematologic malignancy | Acute myeloid leukemia → allogeneic HSCT | Chronic GVHD (oral involvement) | No paraneoplastic antibodies reported | No CNS involvement; oromandibular disorder | Oro-buccal dyskinesia | Jaw clenching + repetitive orobuccal movements | Chronic | Oral discomfort, mucosal cGVHD | Not tested | Prior neuroleptic exposure | CSF not assessed | MRI normal | No PET-CT | Immunosuppression for cGVHD | None | None | Botulinum toxin injections | Marked improvement | AML in remission | cGVHD-induced muscle overactivity | Months |
| Jewell et al., 2021 | Australia | 22/F | Hematologic malignancy | High-risk Ph-negative B-ALL | Post-alloHSCT day +60; possible prior grade 1 skin GVHD | Complete donor chimerism; no relapse markers | Acute CNS-GVHD cerebellitis | Cerebellar ataxia | Gait ataxia, nystagmus, intention tremor, dysmetria | Acute onset | Headache, fatigue, withdrawn affect | All paraneoplastic antibodies negative | None | CSF lymphocytosis 81×10⁶/L, protein ↑, culture/PCR negative | MRI: subtle cerebellar T2/FLAIR ↑ | PET: intense cerebellar hypermetabolism | Myeloablative conditioning + cyclosporine | None | None | High-dose prednisolone; cyclosporine stopped | Mild residual tremor only | ALL remission | Acute T-cell CNS-GVHD | 10 months |
| Grillo et al., 2021 – Case 1 | Italy | 61/F | Lymphoid malignancy | Primary CNS lymphoma (Large B‑cell) | Multifocal basal ganglia disease | Large B‑cell lymphoma (biopsy) | Direct CNS involvement (basal ganglia) | Parkinsonism + mild dystonia | Hypomimia, bradykinesia, gait disturbance, rigidity, L‑hand dystonia, apathy | Insidious progression | Depression, falls | Not tested | None | Not reported | MRI: Caudate, putamen, GP lesions + enhancement | FDG‑PET: hypermetabolic BG lesions | IV methylprednisolone → oral prednisone | None | None | Levodopa ineffective; supportive care | Partial improvement; lost to follow‑up | Started chemotherapy | Tumor infiltration | Not available |
|  |  | 57/F | Lymphoid malignancy | Primary CNS lymphoma (Large B‑cell) | Thalamus + caudate + peduncle + midbrain + periventricular lesions | Large B‑cell lymphoma (biopsy) | Direct CNS involvement (deep gray + midbrain) | Dystonia + choreoathetosis | Left-arm dystonia + chorea; sensory loss | Subacute | Clumsiness, sensory deficit | Not tested | None | Not reported | MRI: Thalamus, caudate, peduncle, midbrain lesions + enhancement | No PET‑CT | Started chemotherapy | None | None | Biperiden ineffective | Lost to follow‑up | Outcome unknown | Motor‑circuit infiltration | None |
| Christensen et al., 2021 | Denmark | 76/M | Lymphoid malignancy | Classical Hodgkin Lymphoma (very late relapse) | Stage IA relapse; prior stage IIIB (12 years earlier) | CD30+, CD15+ Reed–Sternberg cells | Paraneoplastic cerebellar degeneration (Anti‑Tr/DNER positive) | Pancerebellar ataxia | Truncal ataxia, wide‑based gait, dysarthria, dizziness | Subacute (3 weeks) | Pure cerebellar syndrome; no weakness/sensory loss | Anti‑Tr/DNER strongly positive (serum + CSF) | None | CSF: mild pleocytosis, high protein 1.12 g/L, oligoclonal band+ | MRI normal | PET‑CT: FDG‑avid left submandibular node | Gemcitabine + liposomal doxorubicin x 4 cycles | None | None | IVIG x 4 cycles (monthly) | Residual mild ataxia; functional improvement | Complete metabolic remission | Anti‑Tr/DNER Purkinje‑cell autoimmunity due to HL relapse | 16 months |
| Aggarwal et al., 2021 | India | 30/F | Lymphoid malignancy | Hodgkin’s lymphoma (nodular sclerosing) | Axillary, supraclavicular, mediastinal lymphadenopathy | Biopsy: Nodular sclerosing HL | Paraneoplastic cerebellar degeneration | Pan-cerebellar ataxia | Truncal ataxia, gait difficulty, dysarthria, intention tremor, titubation, nystagmus | Chronic with subacute worsening | Axillary swelling, no B symptoms | Antibodies negative | None | Not reported | MRI: mild cerebellar atrophy | PET-CT: cerebellar hypometabolism + FDG-avid nodes | ABVD x 6 cycles | None | None | Supportive neurologic care | Marked neurological improvement | Good hematologic response | Immune-mediated cerebellar degeneration | PET follow-up improved |
| Saini et al., 2020 | USA | 66/F | Lymphoid malignancy | Diffuse large B‑cell lymphoma (DLBCL, ABC subtype) | Stage IV with scalp mass, cervical nodes, bone lesions | CD20+, BCL2+, CD10+, BCL6+, MUM1+ | Paraneoplastic cerebellar degeneration | Generalized cerebellar ataxia | Dysdiadochokinesia, intention tremor, saccadic hypermetria, oscillatory head tremor | Subacute progressive | Headache, scalp mass, tremor | All paraneoplastic antibodies negative | None | CSF normal, cytology negative | MRI normal cerebellum; scalp mass + pachymeningeal thickening | PET‑CT: widespread FDG‑avid lesions | R‑EPOCH → R‑CHOP ×5 + HD‑MTX ×4 | None | None | No specific neurologic therapy | Residual limb ataxia; walked independently | Complete remission | Antibody‑negative PCD | 4 years |
| Petkov et al., 2020 | Bulgaria | 64/M | Lymphoid malignancy | Classical Hodgkin lymphoma (nodular sclerosis) | Single left axillary lymph node (SUVmax 19.9) | CD30+, CD15+, CD3–, CD20–, CD45–, ALK1– | Paraneoplastic limbic encephalitis + OMS | Opsoclonus-myoclonus with ataxia | Opsoclonus, myoclonus, severe ataxia, confusion, hallucinations | Subacute | Visual blurring, memory loss, hallucinations | Anti-Ma2 strongly positive | None | CSF mild pleocytosis, protein 0.43 g/L | MRI limbic T2 hyperintensities | PET-CT hypermetabolic axillary node | ABVD chemotherapy | None | None | IV methylprednisolone + ABVD | Continued recovery | HL early stage | Anti-Ma2 autoimmune encephalitis | Not stated |
| Nagarajan et al., 2020 | USA | 76/F | Lymphoid malignancy | Primary CNS lymphoma (DLBCL) | Bilateral putamen + caudate involvement | Diffuse B-cell type lymphoma (biopsy) | Direct CNS infiltration (basal ganglia) | Parkinsonism | Bradykinesia, rigidity, hypophonia, dysarthria, gait difficulty | Subacute–progressive | Leg weakness, speech difficulty, gait disturbance | Not assessed | None | CSF: elevated 14-3-3 protein, cytology normal | MRI: bilateral caudate/putamen lesions with enhancement | PET-CT: no systemic lymphoma | High-dose methotrexate | None | None | No levodopa response | Recurrence → remission after WBRT | Remission after radiation | Basal ganglia infiltration mechanism | Initial remission 4 months → recurrence |
| Merrill et al., 2020 | USA | 70/M | Lymphoid malignancy | Primary CNS lymphoma (DLBCL) | Recurrent lymphomatosis cerebri; no mass lesion | Autopsy: CD20+ diffuse large B‑cell lymphoma infiltrating BG, thalami, SN, pons | Direct CNS involvement (diffuse infiltration) | Parkinsonism | Shuffling gait, rigidity, dysarthria, impaired balance, anosmia | Subacute | Balance difficulty, speech impairment, left facial weakness | Not assessed | None | CSF: protein 70 mg/dL ↑; cytology & flow negative | MRI: BG enhancement → resolution → new periventricular & cord lesions | PET‑CT: focal decreased uptake near internal capsule | Rituximab ×2; prior HD‑CT + ASCT | None | None | Steroids + carbidopa–levodopa | Progressive decline → death | Relapsed PCNSL at autopsy | Lymphomatosis cerebri involving BG, thalami, SN, pons | 7 months |
| Cao et al., 2020 | China | 29/F | Lymphoid malignancy | Extranodal marginal zone B‑cell lymphoma (MALT) | Anterior mediastinal mass; prior parotid MALT lymphoma | CD20+, BCL2+, CD3−, CD5−, CD10−, CD23−, Cyclin D1−, SOX11−, BCL6−, CD117− | Paraneoplastic cerebellar degeneration | Cerebellar ataxia | Dizziness, gait disturbance, nystagmus, ataxia | Subacute | Sicca symptoms, dysarthria, dyskinesia | All paraneoplastic antibodies negative | None | CSF: oligoclonal bands positive | MRI normal → later cerebellar atrophy | PET‑CT negative post‑treatment | R‑CHOP after tumor removal | None | None | Hydroxychloroquine, prednisone, MMF, rehab | Slow independent walking at 2 years | Lymphoma stable | Immune‑mediated cerebellar degeneration with pSS | 2 years |
| Arratibel et al., 2020 | Spain | 44/M | Lymphoid malignancy | Classical Hodgkin’s lymphoma (mixed cellularity) | Stage IIA | Anti‑Tr positive; classical HL histology | Paraneoplastic cerebellar degeneration | Pancerebellar ataxia | Dysarthria, dizziness, nystagmus, truncal/gait ataxia | Subacute–progressive | Irritability, severe dysarthria, dysdiadochokinesia | Anti‑Tr (DNER) positive | None | CSF non‑diagnostic | MRI: later cerebellar atrophy | PET‑CT: cerebellar hypoactivity + FDG‑avid iliac/inguinal nodes | ABVD x4 cycles | None | None | IVIG x5 days (no improvement) | Persistent ataxia; wheelchair dependence | Complete lymphoma remission | Purkinje‑cell degeneration (anti‑Tr) | 2 years |
| Tee et al., 2019 | Malaysia | 61/F | Lymphoid malignancy | Primary CNS lymphoma (DLBCL, non-GC type) | Multiple CNS lesions: caudate, globus pallidus, peduncle (bilateral progression) | DLBCL nongerminal center subtype (biopsy) | Direct CNS involvement (BG + midbrain) | Parkinsonism | Resting tremor, cogwheel rigidity, bradykinesia, gait unsteadiness | Subacute | Aphasia, choking, left hemiplegia | Not assessed | None | CSF not reported | MRI: multiple enhancing basal ganglia + peduncle lesions | PET-CT: FDG-avid right caudate lesion; no systemic disease | Rituximab + Procarbazine + Methotrexate + Vincristine | None | None | Trihexyphenidyl | Later deterioration | Death due to neutropenic sepsis | Basal ganglia infiltration | Short follow-up |
| Sato et al., 2019 | Japan | 35/F | Lymphoid malignancy | Primary brainstem DLBCL | Right dorsal pons → midbrain | DLBCL (biopsy confirmed) | Direct CNS involvement + HOD | Oculopalatal tremor | Oscillopsia, palatal tremor, dysphagia, dysarthria | Subacute delayed onset | Swallowing impairment, speech difficulty | Not tested | None | CSF not reported | MRI: pontine mass → bilateral IO T2 hyperintensity | PET: uptake at tumor site only | HD-MTX x3 + WBRT 30 Gy + focal RT 10 Gy | None | None | Clonazepam, gabapentin, trihexyphenidyl | Progressive worsening | Ventricular dissemination → death (POD 535) | HOD from tract degeneration | 17 months |
|  |  | 69/M | Lymphoid malignancy | Primary brainstem DLBCL | Left dorsal pons → midbrain | DLBCL (biopsy confirmed) | Direct CNS involvement + HOD | Palatal tremor | Mild palatal tremor, mild dysphagia | Delayed onset | Mild dysphagia | Not tested | None | CSF not reported | MRI: IO T2 hyperintensity (unilateral → bilateral) | PET not reported | HD-MTX x3 + WBRT 30 Gy + focal RT 10 Gy | None | None | No specific therapy | Mild non-progressive tremor | Complete remission POD 432 | HOD from tract degeneration | 14 months |
| Nevison & Rizek, 2019 | Canada | 71/M | Lymphoid malignancy | Diffuse large B‑cell lymphoma (DLBCL) | Left anterolateral pharyngeal/vallecular mass | Monoclonal IgG‑kappa B‑cell population | Paraneoplastic hemichorea (anti‑CV2) | Hemichorea | Left‑sided chorea (leg → face → arm), dysarthria | Acute–subacute | No other neurologic deficits | Anti‑CV2 (CRMP‑5) positive | None | CSF not reported | MRI normal | CT neck: vallecular lesion | R‑CHOP ×3 cycles | None | None | Haloperidol + tetrabenazine | Full neurological recovery | Lymphoma in remission | Anti‑CV2–mediated paraneoplastic chorea | 6 months |
| Herrscher et al., 2019 | France | 52/F | Lymphoid malignancy | Diffuse large B‑cell lymphoma (DLBCL) | Meningeal involvement (cranial nerves V & VII–VIII) | Atypical B‑cell DLBCL (pathology) | Direct CNS meningeal lymphoma | Ataxia | Diplopia, facial palsy, oculomotor palsy, cerebellar ataxia, dysphagia | Subacute–progressive | Fever, tinnitus, worsening cranial nerve palsies | Not tested | None | CSF: lymphocytic meningitis, low glucose, high protein | MRI: meningeal enhancement + cranial nerve involvement | PET‑CT not reported | Rituximab + methotrexate + cytarabine | None | None | Antibiotics → ATT → steroids + cyclophosphamide | Partial neurological recovery | Treatment ongoing | Meningeal DLBCL | Not clearly stated |
| Conway et al., 2019 | USA | 77/F | Lymphoid malignancy history (MCL in remission) | Mantle cell lymphoma (treated); current lesion = PML | Right cerebellar lesion (T2 hyperintense core, rim enhancement) | CD3+ T‑cells, CD20− B‑cells, Cyclin‑D1−, CD68+ macrophages | Not CNS lymphoma → JC virus–positive PML | Cerebellar ataxia | Dizziness, tinnitus, wide‑based gait, incoordination | Subacute‑progressive | Unsteady gait, bilateral tinnitus | Not tested | None | CSF not reported | MRI: right cerebellar lesion with rim enhancement | PET‑CT not reported | No new chemo; prior BR + rituximab maintenance | None | None | Supportive PML therapy | Persistent ataxia | MCL remains in remission | JC virus PML | Not stated |
| Chong et al., 2019 | Singapore | 46/F | Lymphoid malignancy | DLBCL with CNS relapse | Relapsed CNS lymphoma (leptomeningeal) | CD20+, CD79a+, Ki-67 60–70% | CSF malignant B-cells + MRI CNS involvement | Choreoathetosis | Seizure → confusion → involuntary truncal/limb movements | Acute | Drooling, extensor posturing, confusion | Not tested | Methotrexate neurotoxicity | CSF: malignant CD20+ cells | MRI: basal ganglia + periventricular hyperintensity, diffusion restriction | PET‑CT: no systemic recurrence | HD‑MTX + rituximab + dexamethasone | None | None | Levetiracetam + tetrabenazine | Returned to baseline | Lymphoma in remission | MTX toxic leukoencephalopathy | MRI near‑normal by 2 months |
| Reddy SY et al., 2019 | USA | 76/F | Lymphoid malignancy | Primary CNS lymphoma (Diffuse large B-cell type) | CNS-restricted; no systemic disease | Not reported | Bilateral putamen and caudate lesions; biopsy-proven PCNSL | Parkinsonism | Bradykinesia, rigidity, reduced arm swing, dysarthria | Progressively worsening over 6 months | Dysarthria; gait difficulty | Negative/Not reported | None | 14-3-3 elevated (3.7 ng/mL); normal cytology & flow cytometry | Bilateral putamen & caudate T2/FLAIR hyperintensities; heterogeneous enhancement | No extracranial lymphoma | High-dose methotrexate; whole-brain radiation | None | No | Symptomatic treatment | Improved/stable | Remission after treatment | Basal ganglia infiltration; false-positive 14-3-3 | Approx. 6–8 months pre-diagnosis + post-therapy |
| Zurko & Mehta, 2018 | USA | 20/M | Lymphoid malignancy | Primary refractory classical Hodgkin lymphoma | Stage IVB, progressed after ABVD → ICE → brentuximab | No specific molecular markers reported | Immune‑mediated cerebellitis (no CNS lymphoma) | Cerebellar ataxia | Headache, diplopia, nausea, vomiting, dysmetria | Acute | Confusion, dizziness, risk of tonsillar herniation | Not tested | ICI neurotoxicity | CSF: lymphocytic pleocytosis, protein 161 mg/dL, normal glucose | MRI: diffuse cerebellar edema, patchy enhancement | PET‑CT: partial lymphoma response | Nivolumab | None | None | High‑dose dexamethasone | Near‑complete recovery | Partial response | Immune‑mediated cerebellitis | 6 weeks |
| Voeller et al., 2018 | USA | 4/M | Lymphoid malignancy | B‑precursor acute lymphoblastic leukemia | During maintenance therapy; CNS2a | No lymphoma markers; prior vincristine neuropathy; MTX leukoencephalopathy | Autoimmune cerebellitis/encephalitis | Ataxia | Progressive ataxia, weakness, dysarthria, regression | Subacute–progressive | Fevers, diarrhea (CMV colitis), behavioral changes | Autoantibodies negative | None | CSF: normal; OCBs positive | MRI: WM hyperintensities + basal ganglia lesion | PET‑CT not done | Maintenance ALL therapy; ganciclovir | None | None | IVIG → rituximab → dexamethasone pulses | Returned to baseline | ALL treatment ongoing | Autoimmune encephalitis (intrathecal IgG) | Several months |
| Khan et al., 2018 | UK | 31/M | Lymphoid malignancy | ALK‑negative anaplastic large‑cell lymphoma | Systemic lymphadenopathy; new diagnosis | CD30+, ALK‑negative, DUSP22‑negative | No CNS lymphoma; paraneoplastic cerebellar degeneration | Cerebellar ataxia | Slurred speech, vertigo, gait ataxia, dysdiadochokinesis | Subacute (3 weeks) | Recurrent worsening; later cerebellar atrophy | Tr (DNER) antibody positive | None | CSF: normal; OCB positive | MRI: early WM FLAIR lesions → later cerebellar atrophy | PET‑CT: FDG‑avid nodes | CHOP‑like chemotherapy | None | None | IV steroids + IVIG | Persistent ataxia | Lymphoma improved | Paraneoplastic cerebellar degeneration | Not clearly stated |
| Ghannam et al., 2018 | USA | 71/M | Lymphoid malignancy | Primary CNS DLBCL (non‑germinal center) | Multiple cerebellar masses | CD20+, Ki‑67 ~100% | Direct CNS involvement | Cerebellar Ataxia | Blurry vision, dizziness, vomiting, gait imbalance | Acute | Hydrocephalus, vasogenic edema | Not tested | None | CSF not reported | CT: 3 enhancing cerebellar masses; MRI contraindicated | PET‑CT: hypermetabolic cerebellar lesions only | MATRix regimen | None | None | Steroids for edema | Ambulates with walker | No residual disease | Primary cerebellar DLBCL | Not stated |
| Tran et al., 2017 | USA | 46/M | Lymphoid malignancy | Ph+ B‑cell ALL | During Hyper‑CVAD cycle 2B | BCR‑ABL p210 positive | No CNS leukemia | Acute cerebellar syndrome | Confusion, dysarthria, ataxia, encephalopathy | Acute | Encephalopathy, dysarthria | Not tested | No | CSF normal | MRI normal | Not reported | Hyper‑CVAD | Dasatinib | None | High‑dose dexamethasone + methylprednisolone | Persistent mild deficits | Hematologic remission | Drug neurotoxicity | Not stated |
|  |  | 56/M | Lymphoid malignancy | Ph+ B‑cell ALL | During Hyper‑CVAD cycle 2B | Not reported | No CNS leukemia (subdural hygroma) | Acute cerebellar syndrome | Somnolence, dysarthria, ataxia | Acute | Encephalopathy | Not tested | No | CSF normal | MRI: subdural hygroma | Not reported | Hyper‑CVAD | Dasatinib | None | Dexamethasone + prednisone | Residual gait imbalance | Hematologic remission | Drug neurotoxicity | Not stated |
| Toyota et al., 2017 | Japan | 58/M | Lymphoid malignancy (T/NK‑cell) | Primary CNS extranodal NK/T‑cell lymphoma, nasal type | Diffuse infiltrative lymphomatosis cerebri pattern | CD45+, CD3+, CD8+, TIA‑1+, p53+, Ki‑67+, EBV‑encoded RNA+, CD20– | Direct CNS lymphoma involvement (biopsy confirmed) | Myoclonia continua | Continuous left arm myoclonus, dystonic posture, EMG 6–9 Hz | Subacute | Elevated sIL‑2 receptor, persistent limb jerks | Not tested | None | CSF: mildly elevated protein, no malignant cells | MRI: diffuse FLAIR hyperintensity, no enhancement | PET/SPECT: no hypermetabolism | HD‑MTX + procarbazine + vincristine + WBRT + focal RT | None | None | Not specified | Partial neurological improvement | Partial lymphoma response | Subcortical myoclonus from ENKL | Not stated |
| Güngör et al., 2017 | Turkey | 11/M | Lymphoid malignancy | Hodgkin’s lymphoma, nodular sclerosing type | Stage IVB | Anti‑Tr (DNER) positive | No CNS lymphoma (paraneoplastic cerebellar degeneration) | Cerebellar ataxia | Truncal/gait ataxia, dysarthria, diplopia, dysmetria | Subacute | Diplopia, tremors, truncal ataxia | Anti‑Tr positive | None | CSF normal | MRI: normal initially → later cerebellar/cerebral atrophy | PET‑CT: reduced cerebellar uptake | COEP x4 + OCEP x2 | None | None | Plasmapheresis x6 + IVIG | Able to walk unaided with mild ataxia | Lymphoma remission | Anti‑Tr mediated PCD | 6 months |
| Esplin et al., 2017 | USA | 46/M | Lymphoid malignancy (history of Hodgkin lymphoma, remission) | No active lymphoma; focal stiff‑limb syndrome (anti‑GAD‑65) | Chronic | Anti‑GAD‑65 strongly positive; EMG continuous motor unit activity | No CNS lymphoma; autoimmune SLS | Focal stiff‑limb syndrome | Painful rigidity LUE, contractures, reduced ROM | Chronic | DVT, severe spasms, pain | Anti‑GAD‑65 positive | None | No CSF (LP refused) | No MRI/CT | No PET‑CT | Rituximab previously | None | None | Diazepam, baclofen, tizanidine, hydrocodone, divalproex | Persistent symptoms | Lymphoma remission | Autoimmune stiff‑limb syndrome | 30‑day follow‑up |
| Borellini et al., 2017 | Italy | 60/M | Lymphoid malignancy | Classic Hodgkin’s lymphoma | Localized (axillary lymph node) | Anti‑GlyR positive; GAD/VGKC/DPPX/onconeural negative | No CNS lymphoma; PERM (autoimmune) | PERM (rigidity & myoclonus) | Axial rigidity, spasms, dysphonia, gait impairment | Subacute‑progressive | Autonomic dysfunction, urinary retention, dysphagia | Anti‑GlyR positive | None | CSF normal; no OCB | MRI normal (brain & spine); muscle edema on trunk MRI | PET‑CT: active axillary node | ABVD | None | None | Plasmapheresis + clonazepam + gabapentin + steroids | Near‑complete recovery | Lymphoma remission | Anti‑GlyR PERM | 12 months |
| Abruzzese E, 2017 | Italy | 44/M | Classical Hodgkin’s lymphoma, nodular-sclerosis type | Newly diagnosed HL, Stage IIB | Newly diagnosed HL, Stage IIB | Ataxia with lower limb weakness | Difficulty walking, gait ataxia; no CNS infiltration; CSF negative | Neurological symptoms preceded HL diagnosis | ≈1 year | Paraneoplastic etiology suspected | Paraneoplastic neuropathy/cerebellar involvement | Normal; no malignant cells | No CNS lesions described | CT/PET: cervical lymphadenopathy, no mediastinal/splenic uptake | No bone marrow involvement reported | Severe dilated cardiomyopathy (LVEF 27–43%) | High | Modified ABVD with non-pegylated liposomal doxorubicin; bleomycin stopped | 4 cycles | Yes | Stable NYHA I, preserved LVEF | Complete remission | Independent life, returned to work | Alive |
| Zahra K, 2016 | Tunisia | 4/F | Leukemia | B-precursor Acute Lymphoblastic Leukemia (pre-B ALL) | Newly diagnosed; CNS negative | Normal metaphases; no specific markers reported | No CNS leukemia; CSF negative | Cerebellar syndrome | Hypotonia, flaccid quadriplegia, dysarthria; conscious | After 3rd cycle of HD MTX + intrathecal MTX | None (no seizures, no altered sensorium) | Not applicable | Yes – MTX neurotoxicity | No leukemic cells; nondiagnostic | MRI: cerebellar + occipital T2 hyperintensity; resolved | Not performed | Saint Jude protocol; HD MTX + triple intrathecal therapy | None | None | Supportive only; MTX withheld | Complete recovery | In remission | Adenosine-mediated MTX neurotoxicity | 6 months |
| Shimatani Y, 2016 | Japan | 54/M | NK/T-cell lymphoma | Extranodal NK/T-cell lymphoma, nasal type (CNS-restricted) | CNS-restricted; no systemic disease | CD3+, CD56+, granzyme B+, perforin+, TIA-1+; CD4−, CD5−, CD8−, CD20−; EBER+; monoclonal TCR-γ | Diffuse infiltration of brain, spinal cord, meninges, cauda equina (autopsy-proven) | Myoclonus + mild ataxia | RPD, myoclonus, ataxia, cognitive decline, irritability | Symptoms evolving over 2 months before first evaluation | Aphasia, memory loss, inability to walk, sensory loss | None detected | No drug-induced toxicity | Initially normal; later ↑cells, ↑protein, β2‑microglobulin↑, IL‑2 receptor↑, EBV DNA up to 14,000 copies/mL | Brain MRI: progressive atrophy, mild FLAIR lesions, no enhancement; lumbar MRI: Gd‑enhancing swollen cauda equina | No abnormal uptake; whole-body imaging negative | None (diagnosis made only at autopsy) | None | None | IV methylprednisolone + oral prednisolone (no response) | Progressive decline → death at 12 months | No systemic lymphoma; CNS-only disease | EBV‑driven NK/T‑cell malignant infiltration of CNS | 12 months |
| Hatcher-Martin JM, 2016 | USA | 47/F | Leukemia (history) | Childhood Acute Lymphoblastic Leukemia (ALL), long-term survivor | Long-term remission | Not reported | Radiation-induced chronic WM, basal ganglia, cerebellar injury | Freezing of gait (FOG) + dystonia + parkinsonism | Severe FOG, dystonic arm posture, retropulsion, dysarthria, pallilalia | Gait impairment from age 25; FOG by age 33 | Epilepsy, cognitive impairment, dystonia, paratonia, dexterity issues | None | None | Not reported | MRI: diffuse WM injury, putaminal & cerebellar necrosis; GRE mineralization; focal dural enhancement | Not performed | Childhood chemo + whole-brain (24 Gy) + craniospinal radiation (2400 cGy) | None | None | Levodopa/ropinirole/entacapone (no benefit) | Chronic disabling FOG; stable over years | Complete remission from ALL | Delayed radiation neurotoxicity affecting FOG pathways | Long-term follow-up (decades) |
| Grubbs Jr J, 2016 | USA | 53/F | Non-Hodgkin Lymphoma | Primary CNS Diffuse Large B-Cell Lymphoma (DLBCL) | CNS-only lymphoma | CD20+ B-cells; CD68+ reactive T-cells; biopsy proven | Multiple enhancing lesions in cerebrum, thalamus, basal ganglia; biopsy confirmed | Opsoclonus + Myoclonus + Ataxia | Chaotic eye movements, myoclonus of limbs, imbalance, mutism, cognitive decline | Opsoclonus first → myoclonus after 2 months → progression | Mutism, cognitive impairment, functional decline | Negative paraneoplastic antibody panel | None | Acellular CSF; protein 115 mg/dL; cytology x2 negative | MRI: enhancing pons & temporal lesions; FLAIR hyperintensity in thalamus & basal ganglia | No systemic disease on CT chest/abdomen/pelvis | Dexamethasone → MTX 8 g/m² → Vincristine 2 mg → Rituximab 700 mg | Rituximab | None | IVIG x5 days | Progressive decline → hospice | No systemic lymphoma; CNS-only | Paraneoplastic OMS or direct CNS lymphoma effect | 39 days after treatment started |
| Gray DA, 2016 | USA | 66/M | Lymphoma (history) | Large B-cell lymphoma (treated), T9 AIS-C spinal cord injury | Lymphoma in remission | Not reported | No CNS infiltration; drug-induced movement disorder | Myoclonus + Asterixis + Postural/Intentional Tremor | Painful UE myoclonus at rest, asterixis, high-frequency low-amplitude tremor | 3 days after initiation of high-dose TMP-SMX | Functional decline, unable to eat or participate in therapy | Not applicable | Yes – TMP-SMX neurotoxicity | Not performed | No CNS imaging changes; PET/CT used for infection/recurrence workup | PET negative for lymphoma; pneumonitis/chemical toxicity pattern | Past R-CHOP + radiotherapy (not related to this episode) | None | None | Levetiracetam (symptomatic treatment) | Complete resolution after TMP-SMX dose reduced | Lymphoma remains in remission | TMP-SMX–induced reversible neurotoxicity | Several weeks during rehab |
| Chepovetsky J, 2016 | USA | 68/M | Hodgkin lymphoma | Classical Hodgkin lymphoma (CD30+, CD15 partial+, CD20−, CD3−, ALK1−, EBER−) | Retroperitoneal & pelvic lymphadenopathy; pulmonary nodules; marrow negative | CD30+, CD15 partial+, CD20−, CD3−, ALK-1−, EBER− | No CNS lymphoma; paraneoplastic cerebellar degeneration (anti-Tr–mediated) | Cerebellar ataxia | Dysarthria, nystagmus, limb dysmetria, dizziness, falls, inability to ambulate | Symptoms began 2 years before lymphoma diagnosis | Dysarthria, disequilibrium, nystagmus, LOC episode | Anti-Tr (PCA-Tr) strongly positive in serum & CSF | None | Anti-Tr positive; other details not provided | Repeated MRIs normal; no cerebellar atrophy or lesions | Initial PET/CT normal; later PET showed pulmonary nodules & retroperitoneal nodes | CHOP x4 cycles | None | None | IVIG trial (no benefit) | Severe permanent cerebellar dysfunction | Good radiologic response; lymphadenopathy decreased | Anti-Tr–mediated Purkinje-cell degeneration | >2 years before diagnosis + months after |
| Yamamoto J, 2015 | Japan | 70/F | Lymphoma | Primary CNS diffuse large B-cell lymphoma (DLBCL) | CNS-only disease; no systemic or ocular involvement | CD20+, CD3−, Ki-67 92%, large atypical cells | Biopsy-confirmed cerebellar DLBCL; large enhancing mass | Cerebellar ataxia | Progressive gait disturbance, dysmetria, wide-based gait | Several weeks before initial hospital visit | Dysmetria, gait instability, nausea/vomiting | None | None | Not described | CT: mixed-density lesion; MRI: enhancing cerebellar mass, edema; DWI mild restriction; perfusion ↑blood flow | Body CT normal; PET not reported | HD-MTX x1; radiotherapy (renal dysfunction limited MTX) | None | None | 5-ALA–guided partial tumor resection | No neurological deficit at 21 months | No recurrence at 21 months | High cellular density + proliferation explaining 5-ALA fluorescence | 21 months |
| Sáenz-Farret M, 2015 | Mexico/Argentina | 24/M | Non-Hodgkin Lymphoma | Primary CNS Lymphoma (CD20+ B-cell lymphoma) | CNS-only; no systemic disease | CD20+, CD45+ | Basal ganglia & thalamic involvement; MRI + biopsy confirmed | Hemichorea | Involuntary distal > proximal choreiform movements (right side) | 2 weeks before admission | Inattentiveness; later encephalopathy | Not evaluated | None | CSF PCR negative for HSV, CMV, EBV, Toxoplasma, Enterovirus, Parvovirus, LCM | Basal ganglia mass with enhancement + diffusion restriction | PET: one hypermetabolic lesion; no systemic disease | Empirical toxoplasmosis therapy (no benefit) | None | None | No specific chorea therapy given | Progressive decline → death | No systemic lymphoma; confirmed PCNSL at necropsy | Direct basal ganglia infiltration by PCNSL | Few weeks |
| Manir KS, 2015 | India | 21/M | Hodgkin lymphoma | Classical Hodgkin lymphoma (Nodular sclerosis type) | Stage IIIA (inguinal + mediastinal nodes) | CD15+, CD30+, PAX5+, CD45−, CD20− | No CNS lymphoma; MRI brain normal | Cerebellar ataxia | Gaze-evoked nystagmus, intention tremor (LUE), truncal ataxia, dysdiadochokinesia | 3 weeks after onset of lymph node swelling | Vibratory oscillopsia on gaze, clumsiness, dysarthria | Not tested | None | Normal CSF; cytospin negative; TB-PCR negative | Normal MRI brain | FDG-PET: uptake in inguinal & mediastinal lymph nodes | ABVD x 6 cycles | None | None | No specific immunotherapy; improvement followed lymphoma treatment | Complete neurological recovery | Complete metabolic remission | Paraneoplastic anti-Tr–like Purkinje cell dysfunction | 6–10 weeks (full recovery by post-radiotherapy visit) |
| Briongos-Figuero LS, 2015 | Spain | 50/F | T-cell non-Hodgkin lymphoma | Primary diffuse leptomeningeal T-cell lymphoma | CNS-only disease; diffuse leptomeningeal infiltration (autopsy) | ACL+, CD43+, UCHL1+; B-cell markers negative | Diffuse leptomeningeal infiltration of brainstem, cerebellum, meninges (autopsy) | Cerebellar ataxia + Opsoclonus–Myoclonus | Severe truncal ataxia, opsoclonus, limb/trunk myoclonus, diplopia | Symptoms for 4 months; acute worsening 1 week before admission | Cognitive impairment, diplopia, vomiting, disequilibrium | Anti-Hu, Anti-Ri, Anti-Yo negative | None | Protein 79 mg/dL; lymphocytic pleocytosis; cytology negative | MRI normal (T1/T2/FLAIR/DWI); CT normal | CT chest/abdomen/pelvis normal | None (diagnosis only post-mortem) | None | None | High-dose dexamethasone + IVIG | Rapid decline → death on day 15 | Autopsy: diffuse leptomeningeal T-cell lymphoma; no systemic disease | Direct leptomeningeal infiltration of cerebellum/brainstem | 4 months pre-admission + 15 days hospital course |
| Reddy A, 2014 | India | 50/F | Non-Hodgkin Lymphoma | Primary breast diffuse large B-cell lymphoma | Breast + axillary nodes initially; remission after CHOP | High-grade B-cell lymphoma (details not given) | FDG-PET: isolated intense cerebellar hypermetabolism; MRI not done | Cerebellar ataxia | Gait imbalance, dysarthria, headache, vomiting | 3 months after remission | Acute cerebellar dysfunction symptoms | Not performed | None | Not available | MRI not performed | PET/CT: intense isolated cerebellar hypermetabolism | CHOP × 6 cycles → remission | None | None | No neurological treatment (patient died before evaluation) | Rapid deterioration → death | Lymphoma remained in remission | Paraneoplastic cerebellar degeneration most likely | Very short (death soon after PET) |
| Noda et al., 2014 | Japan | 49/M | Lymphoid malignancy | Primary CNS lymphoma (Diffuse large B‑cell type) | Not reported | Not reported | Brain biopsy confirmed PCNSL; MRI lesions in cerebral peduncle, subthalamic nucleus, thalamus, PLIC | Choreoathetosis | Right‑sided choreoathetosis; worsened with action/posture | At presentation | Right hemiparesis, mild dysarthria, UMN facial palsy | Not reported | None | Not reported | FLAIR hyperintensities in left peduncle, subthalamic nucleus, thalamus, PLIC; contrast‑enhancing PLIC lesion | Not reported | High‑dose methotrexate ×3 + whole‑brain radiotherapy (56 Gy + boost) | None | No | None specific; improved with oncologic therapy | Complete recovery | Radiological response | Basal ganglia–thalamocortical pathway disruption | Not reported |
| George et al., 2014 | India | 70/M | Lymphoid malignancy | Primary CNS lymphoma (Diffuse large B‑cell type) | CNS-limited (not staged) | CD20+, LCA+; CK−, GFAP−, CD3−, CD10−, CD5−, Cyclin‑D−, BCL‑2− | Cranial MRI + CSF cytology + biopsy confirming DLBCL; lesions in medulla, 4th ventricle, mesodiencephalon, fornix | Parkinsonism | Rest tremor, cogwheel rigidity, bradykinesia, hypophonia, hypomimia, festinant gait | During illness progression | Intractable vomiting, ataxia, memory loss, ptosis, gaze palsy, dysarthria | Not reported | None | Atypical large round cells on cytospin | Multiple homogeneous enhancing lesions; iso–hypointense T1/T2; diffusion restriction; moderate edema | Not reported | Not reported | Not reported | Not done | Supportive only | Not reported | Not reported | High‑cellularity lymphoma causing brainstem/diencephalic pathway disruption | Not reported |
| Rigual D, 2013 | USA | 53/M | Waldenström’s macroglobulinemia | Tumoral Bing‑Neel Syndrome (direct CNS infiltration by WM) | CNS mass + later diffuse brainstem infiltration; systemic WM active | CD20+, PAX‑5+, rare CD138+; monoclonal IGK peaks (147/201 bp, 234/236/282 bp) | Biopsy‑proven cerebellar mass; CSF positive for monoclonal B cells | Cerebellar ataxia | Dizziness, vomiting, ataxia, dysarthria, dysphagia, facial palsy, paresthesias | Gradual at presentation; worsened over months after surgery | Headache, visual problems, dysphagia, facial palsy | Not applicable | None | CSF: CD45+, CD19+, lambda light chain restriction; monoclonal IGK peaks | Initial MRI: cerebellar mass with edema; later spread to brachium pontis & pons | No PET reported | Multiple prior WM regimens: rituximab+fludarabine → lenalidomide+dex → CHOP → rituximab+bendamustine | Rituximab (systemic) | None | Posterior fossa craniectomy; planned radiation | Progressive decline → death | WM persistent; marrow involved | Direct infiltration of cerebellum, pons, brachium pontis by lymphoplasmacytic cells | >2 years WM course; months of CNS progression |
| Rakocevic et al., 2013 | USA | 57/F | Lymphoid malignancy | Primary cutaneous T-cell lymphoma | Skin-limited disease | Not reported | Autoimmune CNS involvement: SPS with anti-GAD65 | Stiff person syndrome | Painful spasms, truncal stiffness | During chronic lymphoma | Startle response, gait impairment | Anti-GAD65 positive | None | Not reported | Not reported | Not reported | Steroids, hydroxychloroquine, cyclophosphamide, tacrolimus, rituximab, alemtuzumab | Rituximab, alemtuzumab | No | Baclofen, diazepam, PLEX, steroids | Near-complete remission | Marked improvement | Autoimmune/paraneoplastic SPS | ~13 years |
| Pandit et al., 2013 | India | 41/M | Lymphoid malignancy | Peripheral T‑cell lymphoma unspecified (PTCLUS) | Nodal + extranodal with brain metastasis | CD3+, CD4-/CD8-, Ki‑67 variable | Brain parenchymal metastasis + biopsy-proven T‑cell infiltrates | Parkinsonism | Slowness, rigidity, festinant gait, hypomimia | 3 years before diagnosis | Dysarthria, cognitive decline, drooling | Not reported | None | 15 cells/mm3, protein 156 mg/dL, cytology normal | Multiple nodular enhancing + hemorrhagic lesions basal ganglia, midbrain, pons | Not reported | IV methylprednisolone + taper | None | No | Levodopa (poor response), steroids | Decline to wheelchair at 3 months | No definitive treatment | Direct neoplastic infiltration | 3 months |
| Lakshmaiah KC et al., 2013 | India | 68/M | Lymphoid malignancy | Gastric diffuse large B-cell lymphoma | Stage IV (gastric + pulmonary nodules) | CD20+, CD3-, CK-; PCD antibodies negative | Paraneoplastic cerebellar degeneration + limbic encephalopathy | Pan‑cerebellar syndrome | Ataxia, titubation, nystagmus, myoclonus, gait instability | 2 months before diagnosis | Encephalopathy, hallucinations, behavior change | Anti-Yo, Hu, Ri, Ma, Tr negative | None | 10 cells, normal protein/glucose, cytology negative | MRI normal | Gastric FDG-avid lesion + pulmonary nodules | R-CHOP + intrathecal methotrexate/cytarabine/steroid | Rituximab | No | IV methylprednisolone | Marked improvement at 8 months | Complete remission | Paraneoplastic immune-mediated degeneration | 8 months |
| Cachia et al., 2013 | USA | 71/M | Lymphoid malignancy | Primary CNS lymphoma (high‑grade B‑cell) | Localized CNS disease | CD20+ | Biopsy-proven cerebellar lymphoma + HOD on MRI | Cerebellar ataxia, palatal myoclonus | Dysarthria, palatal myoclonus, pendular nystagmus, truncal/appendicular ataxia | 2 weeks before diagnosis | Diplopia, spasticity, nausea/vomiting | Not reported | None | Not reported | Enhancing cerebellar lesion; later hypertrophic left inferior olive | Not reported | Chemotherapy (3 cycles only; poorly tolerated) | None reported | No | Supportive only | Died at 5 months | Poor (incomplete therapy) | Transsynaptic degeneration from dentate nucleus lesion | 5 months |
| Tsai & McGrath, 2012 | Australia | 66/M | History of lymphoma (remote NHL remission) + thymoma history | Stiff-person syndrome (GAD-positive) after thymoma excision & remote NHL | Not applicable | Anti-GAD >2000 U/mL; AChR/MuSK negative | Autoimmune CNS hyperexcitability; no MRI lesion | Stiff-person syndrome | Progressive stiffness, spasms, noise-triggered myoclonus, jaw trismus | 2 weeks worsening; earlier subtle symptoms 4 months prior | Diplopia, vertigo, myoclonus, falls | Anti-GAD strongly positive | None | Elevated protein 0.82 g/L; OCB negative | Normal MRI brain/spine | Normal contrast CT (no malignancy) | NHL previously treated; none currently | None | No | Baclofen, clonazepam, steroids, IVIG, azathioprine | Ambulatory with mild stiffness | NHL in remission | Autoimmune GAD-mediated SPS | Several months |
| Suri et al., 2012 | India | 54/M | Lymphoid malignancy | Nodular lymphocyte-predominant Hodgkin’s lymphoma | Not specified (axillary lymph node) | Anti-Tr antibody positive | PCD with cerebellar/vermian MRI hyperintensities | Pancerebellar syndrome | Truncal ataxia, dysmetria, dysarthria, slow saccades, intentional tremor | Rapid onset over 2 weeks | Sensorineural hearing loss | Anti-Tr positive | None | Normal CSF; no OCB; cytospin negative | T2/FLAIR bilateral cerebellar/vermian hyperintensities | PET normal; CT shows 3 cm axillary lymph node | ABVD x12 cycles | None | No | Supportive | Moderate improvement; truncal ataxia residual | Good chemotherapy response | Autoimmune Purkinje cell degeneration via anti-Tr | During and after 6 cycles (exact duration not stated) |
| Shimazu et al., 2012 | Japan | 55/M | Lymphoid malignancy | Follicular lymphoma Grade 2 (IgH/BCL2 rearranged; marrow 19%) | Stage IV (systemic nodes + lung + marrow) | CD19+, CD20+, Sm-IgM+, Sm-κ+; all PCD antibodies negative | Paraneoplastic cerebellar degeneration; mild cerebellar atrophy | Pancerebellar syndrome | Gait ataxia, nystagmus, dysarthria, limb ataxia; SARA 30 | Progressive 3–4 months | Nystagmus, dysarthria, wide gait | All onconeural antibodies negative | None | WBC 7/mm3; protein 20.5 mg/dL; glucose normal; cytology negative | Mild cerebellar atrophy; no acute lesion | FDG-PET uptake in multiple nodal sites; lung nodular shadows | R-CHOP–like chemo (vincristine omitted) | Rituximab | No | Supportive; recovery with lymphoma therapy | Near-complete recovery; slight dysarthria | Complete remission (two times) | Immune-mediated paraneoplastic Purkinje cell injury | ~2 years |
| Kim et al., 2012 | South Korea | 55/M | Lymphoid malignancy | Burkitt lymphoma of rectum | Not formally staged (systemic chemo given) | Not reported | Chemotherapy‑induced cerebellar dysfunction (PET hypometabolism) | Severe cerebellar ataxia | Gait/limb ataxia, dysarthria, tremor, dysphagia, dysphonia | 7 days after HD-MTX/Ara-C; worsened 4 days after next Ara-C | Tremor, dysphagia, dysphonia; PET cortical/cerebellar hypometabolism | None | High‑dose cytarabine neurotoxicity | Not reported | MRI brain/spine normal | Diffuse cortical/cerebellar hypometabolism | COP → R‑hyper‑CVAD → R‑HD‑MTX/Ara‑C → CALGB IIA → intrathecal therapy | Rituximab | No | Rehabilitation (PT/OT, speech therapy) | Improved but incomplete recovery | Complete remission | Cytarabine-related Purkinje cell toxicity | 10 months |
| Benz et al., 2012 | Switzerland | 65/F | Lymphoid malignancy | Low‑grade B‑cell NHL with medullary spinal cord infiltration | Not staged (spinal infiltration + spleen involvement) | CD20+ lymphocytes in CSF; CD5– B‑cells in spleen | Extensive intramedullary spinal enhancement (C1 downward) | Paroxysmal non‑kinesigenic dyskinesia | Episodic thumb/finger flexion, bilateral/unilateral, 30–40s, spontaneous | Developed 3 weeks after initial symptoms | Tetraparesis, nausea, vomiting, singultus | Not reported | None | CD20+ lymphoma cells; cleared after therapy | Spinal MRI: extensive enhancement → normalized | Not reported | Steroids, intrathecal liposomal cytarabine, rituximab + HD‑MTX; splenectomy | Rituximab | No | Carbamazepine (mild), others ineffective | Full recovery; walking independently | Complete remission | Cervical spinal infiltration causing PNKD | 18 months |
| Ishihara et al., 2011 | Japan | 68/M | Lymphoid malignancy | Extranodal NK/T-cell lymphoma with CNS metastasis | Stage IVB | CD56+ in adrenal biopsy & CSF | Brainstem/cerebellum/periventricular lesions + CSF CD56+ cells | Pure akinesia | Freezing gait, start hesitation, festination, hypomimia, micrographia | After 2 cycles of DeVIC | Confusion, generalized seizure, widespread MRI lesions | Negative | None | 28 cells/mm3; protein 138 mg/dL; CD56+ cells | Extensive T2/DWI/ADC abnormalities; improvement after MTX | Not reported | DeVIC ×2 → MTX pulse + intrathecal Ara-C/MTX/PSL | None | No | L-dopa ineffective; supportive | Full recovery of PA | CNS disease cleared | NKTL infiltration of ponto-mesencephalic circuits | 2 months |
| Hemmaway et al., 2011 | United Kingdom | 60/M | Lymphoid malignancy | Marginal zone lymphoma | Not reported | CD20+; pigmented fungal hyphae consistent with *C. bantiana* | Cystic cerebellar mass; fungal abscess | Cerebellar syndrome | Gait ataxia, horizontal nystagmus | After 6 cycles of R‑CVP | None specified | None | None | Not reported | T1 MRI: multiloculated cystic mass in vermis with edema | Not reported | R‑CVP ×6 (rituximab, cyclophosphamide, vincristine, prednisolone) | Rituximab | No | Neurosurgical excision/drainage | Poor; later death | Not specified | Rituximab-associated immunosuppression enabling fungal CNS infection | Until death |
| Sanz et al., 2010 | Spain | 70/F | Lymphoid malignancy | Classical Hodgkin’s lymphoma (cerebellar relapse) | Stage IV-A initially; isolated CNS relapse | CD30+, EBV-LMP1+ | Biopsy-proven cerebellar mass with necrosis/edema | Cerebellar syndrome | Nausea, gait instability, ataxia, dissymmetry, adiadochokinesia | Sudden onset in Jan 2009 | Hydrocephalus due to mass effect | None | None | Not reported | Enhancing cerebellar mass with necrosis + hydrocephalus | Not reported | ABVD x6 (initial); none at relapse initially | None | No | VP shunt + craniotomy + WBRT | Stable; no recurrence | No systemic recurrence | Hematogenous CNS metastasis | Not stated |
| Pless et al., 2010 | United States | 37/F | Lymphoid malignancy | Intravascular Large B-Cell Lymphoma (IVLBCL) | CNS-restricted disease | CD20+, CD5+, cytoplasmic kappa+, CD10– | Multifocal CNS lesions + CD20+ malignant intravascular cells | Cerebellar ataxia | Dizziness, imbalance, tremor, dysmetria, dysarthria | Gradual onset over 7 months | Paresthesias, aphasia, cognitive slowing, hallucinations, hemiparesis | None | None | Mild pleocytosis, high protein, no malignant cells | Multifocal T2/FLAIR lesions, enhancement, leptomeningeal disease | PET: no systemic lymphoma | HD-MTX → R-CHOP-M | Rituximab | Yes (autologous) | Supportive only | Complete resolution | PET-negative remission | Intravascular occlusion causing CNS ischemia | 19 months |
| Milia et al., 2010 | Italy | 54/F | Lymphoid malignancy | Primary CNS lymphoma (PCNSL) – right frontal lobe | Localized CNS lymphoma | Not reported | Diffuse leukoencephalopathy and cortical–subcortical atrophy | Lower body parkinsonism | Gait difficulty, shuffling, freezing, wide-based gait, instability | 3 years of progression | Freezing worsened by stress; kinesia paradoxa; mild executive dysfunction | None | MTX + WBRT neurotoxicity | Not reported | Diffuse WM hyperintensity; cortical atrophy; ventriculomegaly | Not reported | HD-MTX → WBRT → HD-MTX + ASCT | None | Yes (autologous) | Levodopa (no response); gait therapy | Persistent severe gait impairment | PCNSL in remission | Radiation + MTX-induced WM injury | ≥3 years |
| Lin & Hong, 2010 | Taiwan | 81/M | Lymphoid malignancy | Primary infratentorial large B‑cell lymphoma | CNS‑localized disease | CD20+; CD3–; CD30–; GFAP–; EMA–; ALK– | Enhancing para‑midbrain/dorsal pons mass + hydrocephalus | Parkinsonism | Freezing, gait initiation failure, broad‑based gait, tremor, rigidity, hypomimia | Acute–subacute over days | Hemiparesis, disorientation, raised ICP symptoms | Not reported | None | Normal CSF | CT: midbrain mass + ventriculomegaly; MRI: enhancing para‑midbrain mass; MRS: ↑Cho/NAA + lactate | Not performed | No lymphoma treatment (family declined) | None | No | Levodopa (minimal effect) + VP shunt | Deteriorated; hospice care | No systemic data | Midbrain compression + hydrocephalus impairing nigrostriatal pathways | Short (post‑operative only) |
| Rodis DG et al., 2009 | Italy | 54/F | Primary CNS Lymphoma | Recurrent PCNSL treated with HD-MTX, WBRT, HD-MTX+ASCT | Multiple relapses; post-treatment remission | Not reported | No active lymphoma; treatment-induced leukoencephalopathy | Lower-body parkinsonism | Freezing gait, shuffling, postural instability, symmetric lower-limb rigidity | Chronic | Mild executive dysfunction only | None | None (RT/MTX toxicity) | Normal | MRI: diffuse leukoencephalopathy + atrophy | Not reported | HD-MTX x6, WBRT 40 Gy, HD-MTX + ASCT | None | Yes | Levodopa + physiotherapy (poor response) | Progressive deterioration | Remission | Treatment-induced diffuse leukoencephalopathy | 7–10 years |
| Necioğlu Örken et al., 2009 | Turkey | 6/F | Lymphoid malignancy | B-cell ALL (FAB L1, t(12;21)+) | Standard-risk; CR at day 28 | t(12;21)+ | No CNS leukemia; drug-induced chorea | Generalized chorea | Generalized choreic movements, hypotonia, motor impersistence | 4 days after IT-MTX | Hypotonia, motor impersistence | Not applicable | Intrathecal MTX neurotoxicity | Normal CSF | Normal MRI | Not performed | Induction + consolidation with weekly IT-MTX | None | No | Haloperidol 2 mg/day | Full resolution; no recurrence | In remission | Striatal GABAergic dysfunction from MTX | ≥4 months |
| Kolbaske et al., 2009 | Germany | 60/M | Lymphoid malignancy | Bing–Neel syndrome (Waldenström macroglobulinemia) | Long-standing WM; 5 relapses; no systemic activity | CSF CD19+, CD22+ IgM-producing B-cells; identical IgM-κ in serum & CSF | Diffuse pontine infiltration on MRI; malignant B-cells in CSF | Gait ataxia + intention tremor | Severe gait ataxia, intention tremor, seizure-like events | Progressive over 4 months | Seizure-like events; no cranial nerve deficits | Not reported | None | 50–75 WBC/μL, high protein, intrathecal IgM, malignant B-cells | Diffuse pontine T2/FLAIR hyperintensity; no enhancement | Not reported | Fludarabine + cyclophosphamide; intrathecal rituximab | Rituximab (intrathecal) | No | None specified | Improved but residual ataxia | CSF & MRI normalized | Pontine lymphoplasmacytic infiltration | Not explicitly stated |
| Karmon et al., 2009 | Israel | 29/M | Lymphoid malignancy | Hodgkin’s lymphoma (mixed cellularity) with PCD | Not stated (cervical node only) | CD30+ Reed–Sternberg; anti-Hu/Yo/Ri negative; anti-Tr/mGluR1 negative | Cerebellar leptomeningeal enhancement → cortical atrophy | Cerebellar ataxia + myoclonus | Truncal/appendicular ataxia, nystagmus, dysarthria, axial/limb myoclonus | Acute onset over 5 days | Nystagmus, hypotonia, dysarthria, sluggish gag reflex | Negative | None | Initial: 270 WBC, protein 153 mg/dL; Later: 1 WBC, protein 81 mg/dL | Initial: leptomeningeal enhancement, swelling; Later: cerebellar atrophy | PET-CT negative | ABVD chemotherapy | None | No | Plasmapheresis + clonazepam | Persistent ataxia | Complete remission | Immune-mediated Purkinje cell loss | ≥3 years |
| de Lima et al., 2009 | Brazil | 51/M | Lymphoid malignancy | Acute lymphoblastic leukemia (post-allo HSCT) | Post-transplant; remission | Not reported | None (drug-induced) | Secondary parkinsonism | Rest tremor, bradykinesia, rigidity, hypomimia, reduced arm swing | 2 months after cyclosporine A | None significant | Not applicable | Cyclosporine A neurotoxicity | Not reported | Normal MRI except chronic small subdural hematomas | Not performed | Allo-SCT → CyA → switched to MMF | None | Yes (allo-HSCT) | CyA withdrawal + levodopa/carbidopa | Full recovery | Relapsed leukemia later | Basal ganglia neurotransmission impairment from CyA | 22 months |
| Bota & Dafer, 2009 | USA | 17/M | Lymphoid malignancy | Precursor B-cell ALL | Induction → consolidation; CR; CSF negative | Precursor B-cell phenotype | No CNS leukemia; MTX toxicity | Choreiform movements + ataxia | Confusion, unilateral chorea, ataxia, facial droop, hemiparesis | After 5th intrathecal MTX | Right-sided weakness, encephalopathy | Not applicable | Intrathecal MTX toxicity | Normal CSF | DW-MRI restriction; T2/FLAIR normal; later gliosis | Not performed | Prednisone, vincristine, daunomycin, asparaginase; IT-MTX, Ara-C, 6-MP | None | No | Dexamethasone + leucovorin next MTX dose | Near-complete recovery | Continued ALL therapy | Transient MTX WM metabolic toxicity | ≥2 months |
| Rizzo et al., 2008 | Italy | 8/M | Lymphoid malignancy | Acute lymphoblastic leukemia (ALL) | On chemotherapy; remission | Not reported | None (Tourette syndrome; no CNS leukemia) | Motor + phonic tics | Multiple motor tics, phonic tics, coprolalia, echolalia, rage attacks | Improved during ondansetron therapy | ADHD, OCD, impulsivity | Not applicable | None | Normal CSF | Remote MRI hyperdensity; no active lesion | Not performed | Multidrug ALL chemotherapy | None | No | Ondansetron; later pimozide | Sustained improvement | ALL in remission | 5‑HT3 antagonism reducing dopaminergic overactivity | Until Dec 2006 |
| Gofton et al., 2008 | Canada | Early 60s / F | Lymphoid malignancy | Chronic lymphocytic leukemia | Longstanding treated CLL | Not reported | PML confirmed by biopsy showing SV40-positive oligodendroglial inclusions and EM viral capsids | Limb and gait ataxia | High-stepping gait to right, frequent falls, right arm dysmetria, right-sided dysdiadochokinesia | 4-month progressive course | Diplopia, dysarthria, dysphagia, slowed speech, confusion, bilateral VI palsies, impaired upgaze, right hemiparesis | Negative | None evident; long-term chlorambucil but no toxicity | Clear CSF; mild lymphocytes; protein 278 mg/L; glucose 3.1 mmol/L; CSF flow cytometry normal | Multifocal T2/FLAIR hyperintensities without enhancement, progressive | Not performed | Chlorambucil 2 mg bid | None | None | Supportive | Death due to pneumonia and respiratory failure | CLL stable | JC virus reactivation causing PML | ~1 month after admission |
| Gallagher et al., 2008 | United Kingdom | 64/M | Lymphoid malignancy | T-cell rich diffuse large B-cell lymphoma | Disseminated disease with para-aortic & iliac lymphadenopathy, splenic lesions, vertebral lesion | Not reported | Lymphomatous meningitis suspected; CSF lymphocytic pleocytosis with atypical mononuclear cells; diffuse white-matter lesions on MRI | Extrapyramidal syndrome with bradykinesia; 'Applause sign' | Hypomimia, bradykinesia, paucity of spontaneous movements, slow gait; perseverative clapping | Subacute course over months | Confusion, headaches, right facial pain, urinary incontinence, frontal dysexecutive syndrome, dyspraxia | Not reported | No | Protein 1.58 g/L; 43 WBC (90% lymphocytes); atypical mononuclear cells; PCR negative | MRI FLAIR: diffuse white-matter lesions with partial improvement after chemotherapy | CT: lymphadenopathy + splenic + vertebral lesions (PET not reported) | Idarubicin-based chemotherapy + intrathecal methotrexate + cytarabine | None | None | Supportive + treatment of underlying lymphoma | Partial recovery | Under treatment; partial response | CNS lymphomatous meningitis causing frontal–subcortical dysfunction | 10 months |
| Williams-Gray et al., 2007 | United Kingdom | 39/M | Lymphoid malignancy | Chronic lymphocytic leukemia (CLL) | Remission; post two allogeneic BMTs | Brain biopsy: JC virus DNA+, CD20+, CD3+, CD4+, CD8+ infiltrate | Biopsy-proven PML with JC-virus inclusions | Unilateral parkinsonism | Rigidity, bradykinesia, hypomimia, micrographia, dyspraxia | Progressive over 6 months | Cognitive decline, dysphasia, seizures post-biopsy | Not reported | None | Protein 0.81 g/L; normal glucose; no cells; JC PCR negative | Diffuse hemispheric WM hyperintensity; no basal ganglia lesions; resolution at 1 year | Not performed | Prior chlorambucil, fludarabine, CHOP, alemtuzumab; none during PML | None | Yes (two allogeneic BMTs earlier) | Cabergoline; carbamazepine for seizures | Marked improvement; minimal residual parkinsonism | CLL remission | Cortical–subcortical disconnection from JC-virus demyelination | 12 months |
| Tie et al., 2007 | New Zealand | 30/M | Lymphoid malignancy | Mixed-cellularity classical Hodgkin’s lymphoma | Occult nodal disease | Weak anti-Tr positivity | Paraneoplastic; Purkinje cell loss on biopsy | Cerebellar ataxia | Vertigo, severe gait and limb ataxia, dysarthria, downbeat nystagmus | Progressive over 2 months | Nausea, vomiting, severe imbalance | Anti-Tr (weak positive) | None | Lymphocytosis, elevated protein | Normal MRI repeatedly | FDG‑avid inguinal/iliac nodes; cerebellar hypometabolism | ABVD x3 + radiotherapy | None | No | IV steroids, IVIG, plasmapheresis | Severely ataxic; wheelchair-bound | Excellent hematologic response | Anti-Tr Purkinje cell autoimmunity | 16 months |
| Razzak et al., 2007 | United Kingdom | 47/M | Lymphoid malignancy | Composite lymphoma (Hodgkin’s lymphoma + CD5- BLPD) | Abdominal lymphadenopathy; BM involvement by BLPD | HL: CD30+, CD15+, BOB1+, OCT2+, MUM1+, Bcl2+; BLPD: CD5-, CD20+, CD79b+, IgM+, κ+ | Paraneoplastic; no CNS lymphoma | Pancerebellar ataxia | Nystagmus, dysarthria, limb/truncal ataxia, falls | 3-week progression | Dysarthria, gait disturbance, GI symptoms, weight loss | Anti-Tr positive | None | 57 cells/mm3 (lymphocytes/mononuclear), cultures negative | Normal MRI brain | Abdominal nodes on CT | ABVD x6 | Rituximab monthly x6 | No | IVIG + acyclovir initially | Residual but improved ataxia | Complete remission | Anti-Tr Purkinje cell autoimmunity | 18 months |
| Foy et al., 2007 | United Kingdom | 85/F | Lymphoid malignancy | Primary cerebral DLBCL | CNS-limited; postmortem diagnosis | Not reported | Autopsy-proven CNS infiltration (basal ganglia, SN, thalamus) | Parkinsonism | Tremor, cogwheeling, bradykinesia, shuffling gait, hypomimia | Progressive over 6 months | Apathy, confusion, muffled speech, pyrexia | Not reported | None | Normal CSF; no malignant cells | MRI: temporal atrophy; old cerebellar infarct; no basal ganglia lesion | Not done | None | None | No | Levodopa, pergolide, apomorphine | Progressive decline; death at 9 months | Not applicable | Lymphoma infiltration of SN/striatum | 9 months |
| Ypma et al., 2006 | Netherlands | 34/M | Lymphoid malignancy | Hodgkin’s lymphoma (nodular sclerosis, Stage IIB) | Axillary/cervical nodes; B symptoms | Not reported | Paraneoplastic; no CNS lymphoma | Pancerebellar ataxia | Dysarthria, diplopia, severe limb ataxia, nystagmus, wide gait | Acute onset | Headache, nausea, vertigo | Anti-Tr positive | None | 462 WBC; high protein; monoclonal IgG | Normal early; cerebellar atrophy later | FDG-avid axillary/cervical nodes | EBVP x6 + radiotherapy | None | No | Plasmapheresis | Severe persistent ataxia | Complete remission | Anti-Tr Purkinje cell autoimmunity | ≥6 months |
| Geromin et al., 2006 | Italy | 17/F | Lymphoid malignancy | Hodgkin’s disease (nodular sclerosis) | Initial remission; relapse after PCD onset | Anti-Tr serum 1:6400; CSF 1:1600 | Paraneoplastic; no CNS lymphoma | Pancerebellar ataxia | Vertigo, dysarthria, nystagmus, dysmetria, truncal/appendicular ataxia | Acute onset, worsened in 1 week | Nausea, vomiting, hyperreflexia | Anti-Tr strongly positive | None | Protein 745 mg/L; normal glucose; no malignant cells | Normal CT/MRI | Not performed | Radiation (initial); ABVD x6 (relapse) | None | No | Steroids + monthly IVIG x6 | Near-complete recovery | Complete remission; long-term disease-free | Anti-Tr–mediated Purkinje cell autoimmunity | ≈10 years |
| Denison & Alghzaly, 2006 | Oman | 16/F | Lymphoid malignancy | Precursor B‑cell acute lymphoblastic leukemia (Ph+) | First remission; preparing for allo‑BMT | Philadelphia chromosome positive | No CNS leukemia; Busulfan-induced neurotoxicity | Myoclonus (generalized brief myoclonic seizures) | Brief generalized myoclonus, recurrent episodes lasting seconds; no loss of consciousness | Immediately after last high‑dose IV busulfan dose | None; normal neurological examination | Not applicable | High‑dose IV busulfan neurotoxicity | Normal electrolytes, glucose, vitals; no CSF done | No neuroimaging performed | Not performed | Fludarabine + high‑dose IV Busulfan (conditioning regimen) | None | Yes (planned/received allo‑PBSC transplant) | Prophylactic phenytoin | Recovered fully | Engrafted successfully | Direct busulfan CNS toxicity causing transient myoclonus | Short (peritransplant) |
| Tan JH et al., 2005 | Singapore | 75/M | B-cell lymphoma | Large B-cell lymphoma (occult; diagnosed on repeat marrow biopsy) | Not reported | Not reported | No | PSP-like akinetic-rigid syndrome | Bradykinesia, truncal rigidity, vertical gaze palsy, delayed saccades, dystonic hand posture, dysarthria, apathy, bed-bound | Gradual onset | Peripheral demyelinating neuropathy; encephalopathy (EEG); cognitive dysfunction; hypersomnolence | Anti-Hu, Anti-Ri, Anti-Yo negative | None | 10 WBC/mm³ (lymphocytic), protein 1.09 g/L; normalized at 8 months | MRI mild WM ischemic changes; no midbrain or BG abnormalities | Not done | None | None | None | Levodopa | Died 11 months after onset | Untreated; progressive | Paraneoplastic | 11 months |
| Kumar et al., 2005 | USA | 69/M | Lymphoid malignancy | Large B‑cell non‑Hodgkin lymphoma | Initial presentation with OMS | Not reported | Paraneoplastic (normal MRI, no CNS infiltration) | Opsoclonus‑myoclonus‑ataxia syndrome | Multidirectional saccades, lower limb myoclonus, severe truncal ataxia, somnolence | Acute (1 week) | Nausea, vomiting, somnolence | Not tested | None | Protein 52 mg/dL; RBC 258; single oligoclonal band | MRI normal | Chest CT: mediastinal nodes; lung mass biopsy → lymphoma | Cyclophosphamide + mitoxantrone + corticosteroids | None | No | Clonazepam | Neurological deterioration | Death (2 weeks later) | Paraneoplastic immune‑mediated OMS | Very short |
| Hengstman et al., 2005 | Netherlands | 68/F | T‑cell lymphoma (cutaneous T‑cell) | Mycosis fungoides with CNS metastasis | No visceral disease initially; later CNS spread | Histopathology: atypical lymphocytes with cerebriform nuclei | Direct CNS metastasis to caudate nucleus | Chorea | Left arm chorea, facial asymmetry, mild cerebellar ataxia, ataxic gait | Acute onset; episodes lasting minutes | Backward falls; mild bilateral arm ataxia | None reported | None | No CSF performed | Right caudate mass with enhancement + mass effect | Not done | Planned cranial radiotherapy (refused) | None | No | Supportive care | Died at 7 weeks | Not applicable | Direct basal ganglia infiltration causing chorea | 7 weeks |
| Batchelor et al., 2005 | USA | 35/F | Lymphoid malignancy | Primary CNS diffuse large B‑cell lymphoma | Initial diagnosis (Stage IE) | Bcl‑6 positive; CD20+, CD45+ | Direct CNS lymphoma infiltration (biopsy confirmed) | Ataxia | Gait ataxia, left arm ataxia, truncal instability, left neglect | Subacute (2 weeks) | Headache, nausea, neck pain, left pronator drift, clonus | None | None | No CSF performed | Two enhancing masses—splenium corpus callosum + right parietal/temporal; edema | Not done | High‑dose methotrexate (induction, consolidation, maintenance) | None | No | Supportive care + MTX | Complete remission → relapse at 53 months → remission again | Responded to retreatment | Direct mass-effect + white matter infiltration | ~5 years |
| Jundt et al., 2004 | Germany | 58/M | Lymphoid malignancy | Diffuse large B‑cell non‑Hodgkin lymphoma (relapsed) | Post–autologous SCT | Not reported | No CNS lymphoma; post‑hypoxic myoclonus (Lance–Adams syndrome) | Action myoclonus | Severe resting + action myoclonus; large‑amplitude jerks; stimulus‑sensitive | Dramatic worsening on day 2 of TMP‑SMX | Confusion, disorientation; inability to walk or eat | Not done | High‑dose TMP‑SMX neurotoxicity | No CSF performed | MRI normal | Not done | Autologous SCT earlier; now treated for presumed PCP | None | Yes (autologous SCT already done) | Piracetam + valproate (ineffective) | Marked improvement after dose reduction | Hematologic status stable (post‑SCT) | TMP‑SMX toxicity worsening Lance–Adams syndrome | Short (hospital course) |
| Wiener et al., 2003 | Belgium | 66/M | Lymphoid malignancy | Hodgkin’s lymphoma | Not reported | Not reported | Paraneoplastic; anti-Tr antibodies; no CNS lymphoma | Extremity myorhythmia; limb/truncal ataxia | Rhythmic 180 cycles/min tremor; rest/action/posture; nystagmus; dysarthria | 17 days before admission | Diplopia, nystagmus, saccadic pursuit, skew deviation, dysarthria, ataxia, gait inability, brisk reflexes | Anti-Tr positive initially | None | 40 RBC, 7 WBC (94% lymphocytes), protein 83 mg/dL, OCB-, IgG index normal, viral PCRs negative | CT/MRI normal | PET: abdominal lymph node uptake | ABVD | None | None | Levodopa/pergolide (vomiting) | Persistent myorhythmia; cannot walk unaided | Lymphoma diagnosed; tremor unchanged after 5 cycles | Paraneoplastic cerebellar/brainstem autoimmunity | 14 months |
| Tan et al., 2003 | Singapore | 62/F | Lymphoid malignancy | Primary CNS high‑grade B‑cell lymphoma | Initial presentation | Not reported | Direct CNS lymphoma infiltration (biopsy-confirmed) | Choreoathetosis | Left‑sided choreoathetosis: facial grimacing, shoulder abduction, hip abduction, knee flexion, finger writhing | Acute onset | Mild dysarthria; left UMN facial paresis | None | None | Normal CSF | Bilateral enhancing lesions involving posterior limb of internal capsule, cerebral peduncles, globus pallidus, substantia nigra, subthalamus | Not done | Methotrexate + procarbazine + dexamethasone + intrathecal methotrexate | None | No | Symptomatic + lymphoma therapy | Full remission | Not reported | Direct infiltration of motor pathways | Short |
| Samii et al., 2003 | USA | 49/F | Lymphoid malignancy | Non-Hodgkin’s B‑cell lymphoma (initial intermediate grade; later low‑grade breast B‑cell lymphoma) | Stage IIB | Monoclonal IgM κ | Paraneoplastic CRMP‑5 autoimmune; no CNS lymphoma | Generalized choreodystonia; cervical dystonia; chorea; blepharospasm; myoclonus | Right lateral head tilt, SCM co‑contraction, dysphonic dystonic laryngeal spasm, fragmented motor tasks | Within 3 weeks of rituximab | Dysphonia, dysarthria, oropharyngeal dysmotility, laryngeal spasms, breathing dyscoordination | CRMP‑5 IgG positive | None | Normal CSF; paraneoplastic antibodies negative except CRMP‑5 | MRI brain normal | Initial PET negative; later breast mass found; para‑aortic nodes | Combination chemotherapy (6 cycles) | Rituximab | None | Cyclophosphamide, prednisone, plasma exchange | Persistent chronic paraneoplastic movement disorder | Initial remission; later breast B‑cell lymphoma | CRMP‑5 T‑cell–mediated paraneoplastic striatal dysfunction | >3 years |
| Rollnik et al., 2003 | Germany | 37/M | Lymphoid malignancy | Primary CNS lymphoma | Not reported | Not reported | Right thalamic and basal ganglia infiltration | Paroxysmal kinesigenic dyskinesia (PKD) | Dystonic-athetoid attacks; left arm/hand dystonia; facial dystonia; left foot dystonia; ≥100/day; 20s duration | 1 week before neurology consultation | Mild left facial weakness; sensory prodrome (tingling left forearm) | Not reported | None | Not reported | MRI: right thalamus/basal ganglia infiltration | Not reported | High-dose methotrexate chemotherapy | None | None | Carbamazepine | Complete remission of PKD | Undergoing chemotherapy; outcome not detailed | Thalamic structural lesion causing secondary PKD | Not reported |
| Gottesman & Höke, 2003 | USA | 44/M | Lymphoid malignancy | Primary CNS lymphoma (high-grade B‑cell) | CNS-limited | Monoclonal B-cells in CSF/vitreous | Multifocal CNS lesions; ocular lymphoma | Severe ataxia | Confusion, dysarthria, ataxia, dysmetria, retropulsion | 2 months | Cognitive decline, truncal ataxia, retro-orbital pain | None | None | Lymphocytic pleocytosis; protein ↑; glucose ↓; cytology positive | Midbrain, basal ganglia, periventricular enhancing lesions | Body PET negative | High‑dose IV methotrexate | None | None | Supportive; prior steroids worsened | Improving neurologically | Responding to MTX; no systemic disease | Direct lymphoma infiltration | Short early follow‑up |
| Ferhanoğlu et al., 2003 | Turkey | 26/F | Lymphoid malignancy | Acute lymphoblastic leukemia (ALL), FAB L2, CALLA+, CD13+, pre-B, bcr/abl– | In remission at event | CALLA+, CD13+, bcr/abl– | No CNS leukemia before IT MTX | Acute cerebellar syndrome | Dysarthria, hypotonia, areflexia, quadriparesis, dysmetria, somnolence | Immediately after IT methotrexate | Areflexia, somnolence, inability to sit, quadriplegia | Not applicable | Intrathecal MTX-induced neurotoxicity | Normal; no leukemic cells; non-diagnostic biochemistry | MRI brain/cervical/lumbar normal | Not reported | BFM-ALL-86 protocol; induction → remission; systemic chemo | None | None | IV methylprednisolone 40 mg/day x5 | No deficits; full neurological recovery | ALL in remission | Direct MTX cerebellar toxicity affecting dentate/superior peduncles | Short follow-up |
| Chuang et al., 2003 | USA | 75/F | CLL (stage 0) + solid tumor | CLL and Metastatic SCC (unknown primary) | Metastatic | Not reported | None | Severe parkinsonism | Hypophonia, rigidity, tremor, myoclonus, dysphagia | 2 weeks after chemo | Severe dysphagia | Anti-Hu, anti-Tg, AMA negative | Carboplatin + paclitaxel–induced | Not reported | MRI: mild atrophy/WM disease | None | Carboplatin + paclitaxel; later docetaxel | None | None | Levodopa 1000 mg/day | Independent walking/feeding by 2 wks | On alternate chemo | Reversible dopaminergic dysfunction | 1 year |
|  |  | 60/F | CLL | CML → ALL transformation | Post-BMT | Not reported | None | Parkinsonism (tremor-dominant) | Rest tremor, mild bradykinesia, mild rigidity, shuffling gait | 4 months after chemo | Speech and gait difficulty | Not reported | Busulfan + cyclophosphamide–induced | Not reported | Not reported | Not done | Cyclophosphamide, busulfan, BMT | None | BMT | Levodopa 600 mg/day | Complete resolution | Post-transplant remission | Reversible presynaptic dysfunction | ≥2 months |
| Sheen et al., 2002 | USA | 85/F | Lymphoid malignancy | Recurrent NHL (B-CLL/SLL) | Recurrent systemic disease | Clonal B-cell population | No CNS mass; right basal ganglia perfusion defect (SPECT) | Left hemichorea | Hemichorea L>leg; truncal movements; occasional ballism | Onset after phlebitis/angioedema; worsened over weeks | Gait imbalance; truncal instability | Anti-Ri/Yo/Hu negative | None | Traumatic tap: RBC 74k, WBC 18, protein 65, glucose 69, clonal B-cells | MRI normal except left cerebellar infarct | SPECT: decreased right basal ganglia uptake | Prior chlorambucil; pending oncology | None | None | Prednisone; tetrabenazine | Improved but persistent | Recurrent NHL | Paraneoplastic basal ganglia dysfunction | ~1 year |
| Alderson & Delalle, 2002 | USA | 52/F | Lymphoid malignancy | Primary CNS lymphoma (Diffuse Large B‑cell) | CNS-only disease | CD19+, CD20+, CD22+, CD79a+ | Multiple enhancing brain lesions | Cerebellar ataxia | Gait deviation, dysarthria, saccadic pursuit breakdown, tremor | 4 months before initial admission | Hypersomnia, fatigue, mild sensory symptoms, mild weakness | Anti-Hu, Anti‑Yo negative | None | 2 RBC, 4 WBC, protein 23, glucose 60, no OCB | Initial MRI normal; later multiple enhancing lesions | Not done | High‑dose methotrexate | None | None | Supportive | Progressive decline | Progressive lymphoma | Sentinel lesion phenomenon in PCNSL | >3 years |
| Yeshurun & Dupuch, 2001 | France | 64/M | Lymphoid malignancy | Diffuse large B-cell NHL, Stage IIB | Stage IIB → remission → recurrence | Not reported | No CNS lymphoma; MRI cerebellar cytotoxicity | Acute cerebellar syndrome | Gait ataxia, nystagmus, dysarthria, incoordination | 3 days after second DHAP cycle | Dysarthria, nystagmus, persistent deficits | Not reported | Cytarabine-induced neurotoxicity | Normal CSF repeatedly | MRI: bilateral cerebellar T2 hyperintensity, T1 hypointensity, no enhancement | Not reported | DHAP (cisplatin, cytarabine 2 g/m2, dexamethasone) | None | None | Supportive only | Persistent cerebellar syndrome | Complete lymph node regression | Direct cerebellar cytotoxicity from cytarabine | 6 months |
| Sánchez-Guerra et al., 2001 | Spain | 67/F | Lymphoid malignancy | Primary CNS B-cell lymphoma | CNS-only | High-grade B-cell lymphoma (biopsy) | Multifocal lesions: basal ganglia, periventricular WM, corpus callosum, pons, MCP, cerebellum | Parkinsonism + cerebellar ataxia | Bradykinesia, masked facies, hyperreflexia, shuffling gait, postural instability | Gradual onset | Facial paraesthesia, nausea/vomiting, gait disturbance | Not reported | None | Normal first LP; second LP: protein 85 mg/dL, 15 lymphocytes, no malignant cells | CT/MRI: basal ganglia & periventricular lesions, callosal involvement, pontine/cerebellar mass | Not performed | Diagnostic case (no treatment reported) | None | None | Levodopa trial | Progressive worsening until diagnosis | PCNSL confirmed | Lymphoma infiltration of BG/brainstem | 20 months |
| Rauch et al., 2001 | Germany | 14-month-old girl | Lymphoid malignancy | Common acute lymphoblastic leukemia (c-ALL) | Newly diagnosed; epidural tumour; 96% marrow blasts | Common ALL immunophenotype | C4–C7 epidural tumour causing cord compression | Torticollis (dystonic posture) | Torticollis, painful gait, irritability, staggering, weakness | After car accident; progressive | Weakness, irritability, refusal to lie down | None | None | Protein 190 mg/dL, glucose 51 mg/dL, 1 WBC/mm3 | MRI: extradural tumour C4–C7 | Not reported | ALL-BFM 95 protocol | None | None | Surgical decompression + physiotherapy | Full neurological recovery | Complete remission; disease-free | Cord compression from epidural leukemic infiltration | >4 years |
| Nuti et al., 2000 | Italy | 73/F | Lymphoid malignancy | Non-Hodgkin’s lymphoma (low-grade B-cell) | Recurrent systemic disease | Not reported | No CNS lymphoma (normal CSF & MRI) | Generalized chorea | Chorea of face, tongue, limbs, trunk; dysarthria; gait instability | Acute/subacute over days–weeks | Dysarthria, gait disturbance | Anti-Hu/Ri/Yo negative | None | Normal CSF | MRI normal | Not reported | Cyclophosphamide, vincristine, prednisone | None | None | Haloperidol (ineffective) | Full recovery | Lymphoma remission | Paraneoplastic basal ganglia dysfunction | 1 year |
| Emir et al., 2000 | Turkey | 11/M | Lymphoid malignancy | Hodgkin disease (mixed cellularity) | Stage IV-SB | Not reported | No CNS tumor; MRI: severe cerebellar atrophy | Severe cerebellar ataxia | Ataxic gait, intention tremor, dysarthria, inability to stand/sit unsupported | Progressive over 3 months | Right Horner syndrome (ptosis, miosis, enophthalmos, anhidrosis) | Anti-Tr not tested | None | Not described; no CNS involvement | MRI: marked cerebellar atrophy; no mass lesion | Not reported | ABVD chemotherapy | None | None | Supportive | Improvement after chemotherapy | Partial remission | Paraneoplastic cerebellar degeneration | Early follow-up |
| Pramstaller et al., 1999 | Italy/UK | 75/M | Lymphoid malignancy | Primary CNS lymphoma (DLBCL) | CNS-only (testicular micro-metastasis at autopsy) | CD20+, CD45+, CD10– | Bilateral globus pallidus infiltration (MRI/autopsy) | Pure akinesia (parkinsonian syndrome) | Freezing, initiation failure, hypophonia, micrographia, festination, upright posture | Gradual onset | Urinary incontinence, saccadic initiation difficulty, swallowing difficulty, mild hemiparesis | Not reported | None | Protein 85 mg/dL; normal cytology | CT/MRI: bilateral basal ganglia lesions with edema, hemorrhagic areas | Not performed | IT methotrexate + methylprednisolone; whole-brain RT 30 Gy | None | None | Levodopa trial (no benefit) | Progressive disease | None | Bilateral pallidal infiltration | 7.5 months |
| Benzing et al., 1998 | Germany | 30/M | Lymphoid malignancy | Hodgkin disease (Stage IIA) | Stage IIA | Not reported | No CNS tumor; cerebellar degeneration only | Severe cerebellar ataxia | Diplopia, unsteady gait, dysarthria, limb/truncal ataxia, intention tremor, nystagmus | Progressive over 4 weeks | Tremor, dysarthria, diplopia | Anti-Yo negative; ANA/ANCA/ENA negative | None | Normal CSF; no OCB; no malignant cells | MRI: marked cerebellar atrophy | Not performed | Chemotherapy + radiotherapy for Hodgkin disease | None | None | IVIG + corticosteroids | Persistent severe cerebellar syndrome | Good tumor response | Immune-mediated paraneoplastic Purkinje cell degeneration | >13 months |
| Batchelor et al., 1998 | USA | 67/F | Lymphoid malignancy | Hodgkin’s disease (Nodular sclerosis) | Localized splenic hilar lymph node disease | CD15+, CD30+ RS cells | Basal ganglia abnormalities; no CNS lymphoma | Generalized chorea | Perioral/periorbital chorea, limb chorea, dysarthria, truncal ataxia, dysphagia | 15 months before tumor diagnosis | Dysarthria, dysphagia, vertigo, ataxia, hyperreflexia | Anti-Hu/Ri/Yo negative; non-classical antibody present | None | Pleocytosis initially (28 WBC); later normal CSF | MRI: BG T2 hyperintensity (putamen/caudate) | Not done | Splenectomy + lymph node excision | None | None | IVIG; immunoadsorption; haloperidol intolerant | Progressive decline → death | Diagnosis confirmed via node | Paraneoplastic autoimmune BG/cerebellar dysfunction | 15 months |
| Wyllie et al., 1997 | Canada | 81/M | Lymphoid malignancy | Stage IV-B small lymphocytic lymphoma | Stage IV-B | Not reported | No CNS involvement | Myoclonus | Jerking movements, stiffness, nocturnal worsening | Day 3 of therapy | Stiffness, appetite loss | Not applicable | Yes—chlorambucil-induced myoclonus | Not done | Not done | None | Chlorambucil; then switched to cyclophosphamide | None | None | None | Full recovery | Successful treatment with cyclophosphamide | Drug-induced CNS toxicity | Several cycles |
|  |  | 75/F | Lymphoid malignancy | Stage IV low-grade lymphoma | Stage IV | Not reported | No CNS involvement | Myoclonus | Limb jerks (arms, right hip), headache, visual symptoms | Day 5 of therapy | Headache, visual disturbance, weakness | Not applicable | Yes—chlorambucil-induced myoclonus | Not done | CT head normal | None | Chlorambucil; then switched to cyclophosphamide | None | None | None | Full recovery | Good tumor response | Reversible subcortical CNS toxicity | Multiple cycles |
| Gambardella et al., 1997 | Italy | 59/M | Lymphoid malignancy | Primary intestinal T-cell lymphoma | Jejunum & ileum involvement; perforated tumor | T-cell markers positive | No CNS tumor; MRI shows inferior olive hypertrophy | Action palatal tremor | Facial twitching, orbicularis oculi jerk | During recurrence of abdominal disease | Mild cerebellar ataxia | All paraneoplastic antibodies negative | None | Protein 12.3 mg/dL; no OCB; autoimmune tests negative | MRI: bilateral inferior olive hyperintensity/enlargement | Not reported | Surgical resection + chemotherapy | None | None | Alcohol, clonazepam, valproate (clonazepam partly beneficial) | Progressive worsening | Initial response then deterioration | Olivary degeneration (Guillain–Mollaret triangle) | ~6 months |
| Eder & Nguyen, 1997 | USA | 67/F | Lymphoid malignancy | Hodgkin lymphoma (nodular sclerosis) | Splenic hilar lymph node disease | CD15+, EBV-LMP– | Paraneoplastic cerebellar degeneration + chorea; no CNS lymphoma | Chorea + severe ataxia | Facial/oral dyskinesia, truncal chorea, limb chorea, dysarthria, dysphagia, gait instability | 13 months before admission | Cognitive slowing, dysphagia, pupillary dysfunction | Anti-Hu/Ri/Yo/Purkinje negative | None | 5 WBC, protein 40 mg/dL, glucose 47; no malignant cells | MRI: bright proton-density signal in basal ganglia | Not done | Splenectomy + distal pancreatectomy (diagnostic) | None | None | Protein‑A immunoadsorption, lorazepam, quinine | Progressive decline; lost to follow‑up | Hodgkin lymphoma confirmed; no therapy given | Autoimmune paraneoplastic BG + cerebellar dysfunction | 15 weeks inpatient |
| Symonds et al., 1994 | UK | 72/F | Lymphoid malignancy | Undifferentiated thyroid NHL | Localized neck disease | Not reported | Paraneoplastic cerebellar degeneration | Gait ataxia | Severe gait instability, truncal imbalance, falls | Worsened during chemotherapy | Cerebellar tremor later | Not tested | Vincristine contribution possible | Not reported | CT/MRI normal | None | Radiotherapy 45 Gy + COP x6 | None | None | Supportive | Persistent disabling ataxia | Complete remission 7 years | Immune Purkinje cell loss | 7 years |
| Krishnan & Bockenstedt, 1994 | USA | 22/F | Lymphoid malignancy | Hodgkin lymphoma (nodular sclerosis) | Bulky Stage IIA | Not reported | Paraneoplastic cerebellar degeneration; no CNS lymphoma | Acute cerebellar syndrome | Vertigo, nystagmus (down-beating + horizontal/upward), ataxia, dysarthria, confusion | 1 week before admission | Babinski signs, cognitive dysfunction, dysphasia | Not tested | None | 140 WBC (91% lymphocytes), protein 90 g/L, glucose normal | MRI brain/spine normal; CT chest mediastinal mass | Not done | ABVD x6 cycles | None | None | Steroids + benzodiazepines | Severe long-term disability (wheelchair-bound) | Complete remission | Autoimmune Purkinje cell degeneration | Months |
| Howell & Sagar, 1994 | UK | 22/F | Lymphoid malignancy | High-grade non-Hodgkin lymphoma | Large mediastinal mass | Not reported | No CNS lymphoma; delayed chemo/radiation toxicity | Parkinsonism | Rest/action tremor, bradykinesia, cogwheel rigidity, stooped posture, hypomimia | 4 months post-therapy | No sensory loss; normal reflexes; autonomic tests normal | Not tested | Vincristine + IT methotrexate + cranial irradiation toxicity | Normal | MRI/CT/SPECT all normal | Not done | CHOP x6 + intrathecal methotrexate + cranial irradiation | None | No | Levodopa/carbidopa | Stable parkinsonism >1 year | No relapse reported | Delayed basal ganglia toxicity | >1 year |
| Kay et al., 1993 | UK | 22/F | Lymphoid malignancy | Hodgkin’s disease (lymphocyte-depleted, BNLI II) | Stage IV-B | Not reported | Paraneoplastic opsoclonus–myoclonus; no CNS lymphoma | Opsoclonus–myoclonus | Chaotic saccades, limb/head myoclonus, tremor, unsteady gait | 7 weeks after BEAM + ABMT | Headache, tremors; no cerebellar signs | Not tested | None | Normal CSF | CT/MRI normal | Not done | LOPP + EVAP x8; later BEAM + ABMT | None | Yes (ABMT) | IV methylprednisolone x3 days | Full recovery | Disease decreased, no relapse reported | Paraneoplastic immune mechanism | ~12 months |
| Topcu et al., 1992 | Turkey | 8/M | Lymphoid malignancy | Hodgkin disease (mixed cellularity) | Mediastinal + cervical nodes | Not reported | Paraneoplastic cerebellar degeneration; no CNS lymphoma | Cerebellar ataxia | Nystagmus, wide-based gait, dysmetria, dysarthria, dystonia | Acute onset at age 8 | Dysarthria, dystonia; no pyramidal signs | Not tested | None | Normal | CT brain normal | Not done | Chemotherapy + radiotherapy | None | No | Supportive | Residual mild deficits | Near-complete remission | Autoimmune Purkinje-cell degeneration | Years |
| Schiff & Ortega, 1992 | USA | 5/F | Lymphoid malignancy | Acute lymphoblastic leukemia (FAB L1) | Bone marrow: 48% blasts, 21% eosinophils | Mixed B-lymphoid/myeloid phenotype | No CNS leukemia (normal MRI/CSF) | Chorea | Limb jerks, facial twitching, dysarthria, chewing difficulty, gait disturbance | ~2 weeks after febrile illness | Dysarthria, balance difficulty | Lupus anticoagulant positive | None | 0 WBC, protein 23 mg/dL, glucose 61 mg/dL, cultures negative | MRI brain normal; EEG normal | Not done | Prednisone, vincristine, L-asparaginase, daunomycin | None | No | Haloperidol | Complete resolution | Remission marrow at day 28 | Antiphospholipid-related basal ganglia dysfunction | 5 months |
| Oliveras et al., 1988 | Spain | 67/F | T‑cell lymphoproliferative disorder | Lymphomatoid granulomatosis (angiocentric T‑cell lymphoma) | Systemic (lung + spleen involvement) | T‑cell phenotype (LCA+, UHCL‑1+, MT‑1 weak+, MB‑2−) | Clinical parkinsonism; MRI periventricular lesions; no CNS mass | Parkinsonism | Festinating gait, rigidity (R>L), rest tremor, confusion, inability to walk | Onset ~1985; progressed over 1–2 years | Confusion; gait failure | None | None | 3 lymphocytes, protein 32 mg/dL, glucose 100 mg/dL | CT normal; MRI symmetric periventricular hyperintensity | Not done | Prednisone + cyclophosphamide; CHOP x3 | None | No | Steroids | Normal neurological exam at discharge | Disease inactive at 10 months | Basal ganglia ischemia from angiocentric T‑cell lymphoma | 10 months |
| Poewe et al., 1988 | Austria | 66/F | Primary CNS lymphoma | Primary CNS lymphoma (basal ganglia/thalamus lesions) | CNS-limited multifocal disease | Not reported | Direct CNS infiltration (thalamus, caudate, corpus callosum) | Chorea → segmental dystonia | Left-arm chorea; later right hemifacial dystonia, torticollis, gait unsteadiness | 1 year after uveitis/papillitis | Mental slowing, hyperreflexia, falls, extensor plantars | Not tested | None | Protein 101 mg/dL, no pleocytosis | CT/MRI: enhancing thalamic + caudate lesions | Not done | Whole-brain radiotherapy | None | No | Haloperidol (ineffective) | Chorea subsided; dystonia improved | Tumor regression | Basal ganglia infiltration | Not specified |
| Ascher & Delaney, 1988 | Philippines | 11/M | Lymphoid malignancy | Acute lymphoblastic leukemia (ALL) | Relapsed bilateral testicular leukemia; marrow & CNS in remission | Not reported | No CNS leukemia; drug‑induced dystonia | Acute dystonia | Severe orbicularis muscle contortions; facial dystonia during etoposide infusion | During etoposide infusion (weeks 54 & 56) | None | Not applicable | Yes — etoposide‑induced acute dystonia | Not done | Not done | Not done | Vincristine–prednisone induction; cyclophosphamide–asparaginase consolidation; relapse protocol incl. etoposide | None | No | IV diphenhydramine 25 mg; prophylactic 50 mg | No recurrence with prophylaxis | Continued chemotherapy | Acute extrapyramidal reaction to etoposide | Weeks 54–58 |
| Gherardi et al., 1985 | France | 59/M | Primary CNS lymphoma | Diffuse large-cell B‑cell lymphoma | CNS-limited multifocal disease | B‑cell phenotype (Ig‑positive cells) | Direct infiltration of substantia nigra, thalamus, cortex | Parkinsonism | Bradykinesia, tremor, rigidity, hypomimia; later asterixis, hemiplegia, dysarthria | At presentation (1977) | Memory impairment, hemiplegia, sensory loss, asterixis | Not tested | None | Not studied | CT: atrophy + ventricular enlargement; no mass | Not done | None (ACTH only) | None | No | L‑dopa 500 mg/day | Progressive deterioration → death | Diagnosis only at autopsy | Direct substantia nigra infiltration | ~5 months |
| Bejar, 1984 | USA | 1/M | Lymphoid malignancy | Acute lymphoblastic leukemia (ALL) | High WBC 75,600; 72% blasts | Not reported | No CNS leukemia; exam normal before dyskinesia | Acute dyskinesia / choreoathetosis | Orofacial dyskinesias, blinking, chewing, tongue protrusion, neck movements, limb dyskinesias | 2 days after 14th dose of Compazine | No fever; alert; movements worsened with touch | Not applicable | Yes — Compazine-induced dyskinesia | Not done | Not done | Not done | Chemotherapy for ALL | None | No | None (haloperidol recommended but not given) | Died 3 days after onset | Leukemia active | Neuroleptic-induced tardive-type dyskinesia in infant | 3 days |
| Sinniah & Lin, 1979 | Malaysia | 11/M | Lymphoid malignancy | Lymphocyte-depleted Hodgkin’s disease (Stage IV-B) | Stage IV-B; lymphadenopathy, hepatosplenomegaly | Not reported | No CNS involvement; normal CSF, brain scan | Extrapyramidal tract syndrome (dystonia) | Dystonia, rigidity, tongue protrusion, grimacing, pill-rolling tremor | 4 months after stopping MOPP | Brisk reflexes, weight loss, difficulty swallowing | Not tested | Suspected MOPP-induced neurotoxicity | Normal | Normal X-rays; normal brain scan | Not done | MOPP (mustine, vincristine, procarbazine, prednisolone) | None | No | Valium, haloperidol, benzhexol, pyridoxine, orphenadrine, thalamotomy | Progressive worsening | Unclear remission | Procarbazine-related neurotoxicity | ~2 years |
| Bean & Ladisch, 1977 | USA | 12/M | Lymphoid malignancy | Acute lymphocytic leukemia (ALL) | Recurrent marrow + meningeal relapses | Not reported | No CNS leukemia; subdural membranes without leukemic cells | Severe chorea/choreoathetosis | Choreiform movements face/trunk/limbs; severe dysarthria; dazed; interfered with actions | Developed over 2 weeks after LP + intrathecal MTX | Lethargy, hemiparesis, papilledema, spasticity | Not tested | No | Opening pressure 220; 0 cells; protein 40 mg/dL; glucose 50 mg/dL | CT: large left subdural hematoma with midline shift | Not done | Systemic chemo + intrathecal MTX/Ara-C | None | No | Surgical evacuation; phenytoin postop | No recurrence; hemiparesis improved | Later leukemia relapse controlled | Basal ganglia pathway compression from subdural hematoma | ≥1 year |
| Horwich et al., 1966 | UK | 19/M | Lymphoid malignancy | Hodgkin’s disease | Widespread lymphadenopathy; liver and spleen involved | Not available (1966 era) | No CNS tumor; autopsy: cerebellar degeneration | Cerebellar ataxia | Gait, limb, and truncal ataxia; nystagmus; dysarthria; palatal weakness | 4 months before Hodgkin’s diagnosis | Diplopia, vomiting, weak gag reflex | Not done | No | 0 cells; protein 60 mg/dL | Normal angiography; no intracranial mass | Not done | ACTH; cyclophosphamide | None | No | ACTH trial | Progressive decline → death | Progressive Hodgkin’s disease | Paraneoplastic cerebellar degeneration | 10 months |

ACA = Anti-cerebellar antibody / Anti-cerebellar ataxia; ALL = Acute Lymphoblastic Leukemia; AML = Acute Myeloid Leukemia; ASCT = Autologous Stem-Cell Transplantation; BV-CHP = Brentuximab Vedotin + Cyclophosphamide + Doxorubicin + Prednisone; CALLA = Common Acute Lymphoblastic Leukemia Antigen (CD10); CBA = Cell-Based Assay; CLL = Chronic Lymphocytic Leukemia; CNS = Central Nervous System; COP = Cyclophosphamide + Vincristine + Prednisone; CR = Complete Remission; CRMP-5 = Collapsin Response Mediator Protein-5 (also known as CV2); CSF = Cerebrospinal Fluid; CST = Corticospinal Tract; CTCAE = Common Terminology Criteria for Adverse Events; cGVHD = chronic Graft-versus-Host Disease; DA-EPOCH = Dose-Adjusted Etoposide + Prednisone + Vincristine + Cyclophosphamide + Doxorubicin; DHAP = Dexamethasone + High-dose Cytarabine + Cisplatin; DLBCL = Diffuse Large B-Cell Lymphoma; DNER = Delta/Notch-like EGF-related Receptor (target antigen of anti-Tr antibody); DWI = Diffusion-Weighted Imaging; EBV = Epstein–Barr Virus; EBER-ISH = EBV-Encoded RNA In Situ Hybridization; EMG = Electromyography; FDG = Fluorodeoxyglucose; FISH = Fluorescence In Situ Hybridization; FLAIR = Fluid-Attenuated Inversion Recovery; FOG = Freezing of Gait; GAD = Glutamic Acid Decarboxylase; GlyR = Glycine Receptor; GP = Globus Pallidus; GVHD = Graft-versus-Host Disease; GZL = Gray Zone Lymphoma; HD-MTX = High-Dose Methotrexate; HL = Hodgkin Lymphoma; HOD = Hypertrophic Olivary Degeneration; HRCT = High-Resolution Computed Tomography; HSCT = Hematopoietic Stem-Cell Transplantation; ICI = Immune Checkpoint Inhibitor; IPI = International Prognostic Index; IO = Inferior Olive / Inferior Olivary nucleus; IT = Intrathecal; JCV = JC Virus; LPD = Lymphoproliferative Disorder; MATRix = Methotrexate + Cytarabine + Thiotepa + Rituximab; MCP = Middle Cerebellar Peduncle; MDS = Myelodysplastic Syndrome; MMF = Mycophenolate Mofetil; MMSE = Mini-Mental State Examination; MOPP = Mustine (Mechlorethamine) + Vincristine (Oncovin) + Procarbazine + Prednisone; MRD = Minimal Residual Disease; NHL = Non-Hodgkin Lymphoma; NK/T = Natural Killer / T-cell; NLPHL = Nodular Lymphocyte-Predominant Hodgkin Lymphoma; NMDARE = N-Methyl-D-Aspartate Receptor Encephalitis; NMDAR = N-Methyl-D-Aspartate Receptor; OCB = Oligoclonal Bands; OMS = Opsoclonus–Myoclonus Syndrome; PCD = Paraneoplastic Cerebellar Degeneration; PCNSL = Primary Central Nervous System Lymphoma; PD-1 = Programmed Death-1; PERM = Progressive Encephalomyelitis with Rigidity and Myoclonus; PET-CT = Positron Emission Tomography–Computed Tomography; PhIP-seq = Phage Immunoprecipitation Sequencing; PKD = Paroxysmal Kinesigenic Dyskinesia; PML = Progressive Multifocal Leukoencephalopathy; PNKD = Paroxysmal Non-Kinesigenic Dyskinesia; PNS = Paraneoplastic Neurological Syndrome; R-CVP = Rituximab + Cyclophosphamide + Vincristine + Prednisone; R-EPOCH = Rituximab + Etoposide + Prednisone + Vincristine + Cyclophosphamide + Doxorubicin; RGS8 = Regulator of G-Protein Signaling 8; RS = Reed–Sternberg (cells); SARA = Scale for the Assessment and Rating of Ataxia; SCC = Squamous Cell Carcinoma; SLS = Stiff-Limb Syndrome; SMZL = Splenic Marginal Zone Lymphoma; SN = Substantia Nigra; SPECT = Single-Photon Emission Computed Tomography; SPS = Stiff-Person Syndrome; STN = Subthalamic Nucleus; SUV = Standardized Uptake Value; SWI = Susceptibility-Weighted Imaging; TBZ = Tetrabenazine; TMP-SMX = Trimethoprim–Sulfamethoxazole; Tr = Tracer (former name of anti-DNER antibody); UMN = Upper Motor Neuron; VGCC = Voltage-Gated Calcium Channel; WBRT = Whole-Brain Radiotherapy; WM = White Matter

References

1. Yawata G, Kimura M, Tanaka T, Saito Y, Chihara N. Anti-Tr/DNER antibody-associated rapidly progressive cerebellar degeneration in anaplastic large cell lymphoma: A case report with literature review. J Neuroimmunol. 2025;408:578732. doi:10.1016/j.jneuroim.2025.578732.
2. Wadhera S, Swain RN, Saini S, Singh C, Jain A, Bal A, et al. Advanced lymphomatoid granulomatosis involving the central nervous system and lung parenchyma presenting with speech and gait disturbances. Indian J Hematol Blood Transfus. 2025;41(3):736–8. doi:10.1007/s12288-024-01860-5.
3. Venkatesan A, Romero JM, Harrold GK, Klontz EH. Case 19-2025: A 69-Year-Old Man with Headache and Ataxia. N Engl J Med. 2025;393(2):176-184. doi:10.1056/NEJMcpc2412528.
4. Vasey O, Francisco T, Espiridion ED. Methylphenidate-induced dyskinesia in a 47-year-old female with acute lymphoblastic leukemia. Cureus. 2025;17(2):e78995. doi:10.7759/cureus.78995.
5. Tilley B, Kim VS, Lass E, Masellis M, Silverstein WK. Rhombencephalitis in an 86-year-old woman with chronic lymphocytic leukemia. CMAJ. 2025;197(1):E9–E12. doi:10.1503/cmaj.240592.
6. Kadubandi A, Midathada M, Arcot Jayagopal L. Hodgkin lymphoma presenting as paraneoplastic cerebellar degeneration: A case report. SAGE Open Med Case Rep. 2025;13:2050313X251328391. doi:10.1177/2050313X251328391.
7. Iguchi T, Furuya T, Koinuma T, Nakajima A, Fuse A, Eguchi H, et al. Lymphomatosis cerebri presenting with rapidly progressive parkinsonism and Holmes tremor: a case report. BMC Neurol. 2025;25(1):392. doi:10.1186/s12883-025-04426-8.
8. Adibi A, Adibi I, Danaei H. Anti-Tr/DNER antibody-associated ataxia in a pediatric Hodgkin lymphoma survivor: successful treatment with plasmapheresis and IVIG. Pediatr Blood Cancer. 2025;72(4):e31594. doi:10.1002/pbc.31594.
9. Silva L, Laranjinha I, Casais C, Samões R. Subacute parkinsonism induced by immune checkpoint inhibitors. Ann Mov Disord. 2024;7(3):224–226. doi:10.4103/aomd.aomd_43_24.
10. Peter E, Ciano-Petersen NL, Do LD, Perrot J, Ngo T, Pluvinage J, et al. Anti-RGS8 paraneoplastic cerebellar ataxia is preferentially associated with a particular subtype of Hodgkin’s lymphoma. J Neurol. 2024;271(10):6839–6846. doi:10.1007/s00415-024-12618-4.
11. Handzic A, Brossard-Barbosa N, Mandell D, Lou SK, Margolin E. Anti-Ma2 antibody-mediated paraneoplastic cerebellar degeneration and myeloneuropathy secondary to lymphoma. J Neuroophthalmol. 2024;44(1):129–132. doi:10.1097/WNO.0000000000002048.
12. Donaghy R, Singer L, Dixit K. Intrathecal methotrexate, central nervous system toxicity, and response to N-methyl-D-aspartate antagonism: An adult case series. Neurooncol Pract. 2024;11(5):665–669. doi:10.1093/nop/npae051.
13. Arora K, Singh AS, Bedi R, Dang K, Kaur A. Navigating the uncharted: a rare case of ocular flutter and intermittent jaw movements in a case of chronic lymphoid leukemia. Ann Indian Acad Neurol. 2024;27(3):313–315. doi:10.4103/aian.aian_772_23.
14. Yokota Y, Hara M, Oshita N, Mizoguchi T, Nishimaki H, Hao H, et al. Anti-N-methyl-D-aspartate receptor antibody-associated autoimmunity triggered by primary central nervous system B-cell lymphoma: a case report. Front Neurol. 2023;13:1048953. doi:10.3389/fneur.2022.1048953.
15. Varela FJ, Chaves H, Rossi M. Teaching NeuroImage: Shrimp Sign in Ataxic Cerebellar Progressive Multifocal Leukoencephalopathy. Neurology. 2023;101(20):918–919. doi:10.1212/WNL.0000000000207767.
16. Samaha S, Larner AJ. Cerebellar syndrome: cause cured, but symptoms persist. Prog Neurol Psychiatry. 2023;27(4):27–29. doi:10.1002/pnp.823.
17. Mesbah-Oskui L, Alabkal J, Alduaij W, Dhawan PS. A unique case of a fulminant clonal CD8-positive T-cell lymphoproliferative disorder with CNS involvement. BMC Neurol. 2023;23(1):329. doi:10.1186/s12883-023-03446-z.
18. Franzini A, Zekaj E, Bona A, Ciuffi A, Porta M, Servello D. Fluorescein sodium-guided resection of a cerebellar lymphoma: case report and literature review. Br J Neurosurg. 2023;37(4):671–674. doi:10.1080/02688697.2018.1556779.
19. Fakhari MS, Poorsaadat L, Talebi HM, Kosari F. Stiff-person syndrome revealing an occult gray zone lymphoma: A diagnostic challenge. Clin Case Rep. 2023;11:e8328. doi:10.1002/ccr3.8328.
20. Algahtani H, Absi A, Shirah B, Al-Maghraby H, Algarni H. Hyperferritinemia with iron deposition in the basal ganglia and tremor as the initial manifestation of follicular lymphoma. Int J Neurosci. 2023;133(8):896–900. doi:10.1080/00207454.2022.2127507.
21. Kirkedal CH, Høyer T, Christensen PB. Subacute parkinsonism as first symptom of primary CNS lymphoma. Ugeskr Laeger. 2022;184(18):V09210723.
22. Fiorelli N, Fraticelli S, Bonometti A, Diamanti L, Paoletti M, Franciotta D, et al. Hodgkin lymphoma with diplopia and nystagmus: a paraneoplastic cerebellar degeneration with ectopic expression of DNER antigen on Reed–Sternberg cells. Clin Lymphoma Myeloma Leuk. 2022;22(2):e124–e127. doi:10.1016/j.clml.2021.10.011
23. Feng Y, Long X, Li X. Wernicke encephalopathy with extensive cortical lesions combined with diffuse large B-cell lymphoma. Neuro Endocrinol Lett. 2022;43(7-8):361–365.
24. Ahn BJ, Go H, Kwon KY. An unusual case of central nervous system lymphoma presenting with ataxic quadriparesis showing ‘wine-glass’–like appearance. J Clin Neurol. 2022;18(3):367–369. doi:10.3988/jcn.2022.18.3.367.
25. Storti B, Gallone A, Paris L, Foresti C. A rare case of atypical palatal-lingual tremor associated with Waldenström's macroglobulinemia. Mov Disord Clin Pract. 2021;8(5):785–787. doi:10.1002/mdc3.13233.
26. Okano R, Suzuki K, Nakano Y, Yamamoto J. Primary central nervous system lymphoma presenting with parkinsonism as an initial manifestation: A case report and literature review. Mol Clin Oncol. 2021;14(5):95. doi:10.3892/mco.2021.2253.
27. Nothrop R, Lee W, Lee D, Gilligan AK. Chorea as a paraneoplastic syndrome heralding the transformation of non-Hodgkin lymphoma. Med J Aust. 2021;214(3):127–128.e1. doi:10.5694/mja2.50878.
28. Nanda S, Handa R, Prasad A, Anand R, Zutshi D, Dass SK, et al. Paraneoplastic cerebellar degeneration as a presenting manifestation of non-Hodgkin’s lymphoma. Neurol Sci. 2021;42(6):2523–2525. doi:10.1007/s10072-020-04965-3.
29. Martínez-Burbano B, Castro-Uquillas E, Martínez-Núñez A. Stiff person syndrome of paraneoplastic cause: Diagnostic keys in a rare entity. Rev Ecuat Neurol. 2021;30(1):200–205.
30. Makranz C, Arkadir D, Nachmias B, Gatt ME, Eliahou R, Atlan K, et al. Neurological misdiagnoses of lymphoma. Neurol Sci. 2021;42(5):1933–1940. doi:10.1007/s10072-020-04807-2.
31. Kitamura W, Ennishi D, Yukawa R, Sasaki R, Yoshida C, Takasuka H, et al. Nodal Peripheral T-cell Lymphoma with T Follicular Helper Phenotype Presenting as Chorea during Treatment: A Case Report and Literature Review. Internal Medicine. 2021;60(19):3155–60.
32. Khera S, Shijith KP, Goswami JN. Methotrexate encephalopathy presenting as choreoathetosis. BMJ Case Rep. 2021;14(2):e241509. doi:10.1136/bcr-2020-241509.
33. Kajtazi NI, Khalid E, Ghamdi JA, Abulaban A, AlHameed MH. Severe ataxia uncovered Hodgkin’s lymphoma: do not forget CT neck when looking for covert malignancy. BMJ Case Rep. 2021;14(11):e245225. doi:10.1136/bcr-2021-245225.
34. Joshi P, Kiers L, Evans A, Ritchie D, Szer J, Cruse B. Oromandibular parafunction in chronic graft-versus-host disease: novel association and treatment approach. Intern Med J. 2021;51(11):1950–1953. doi:10.1111/imj.15567.
35. Jewell KE, Kuzich JA, Lee ST, Trethowan R, MacDonell R, Schwarer AP. Acute graft-versus-host disease associated cerebellitis as the cause of pyrexia of unknown origin detected with 18F-FDG-PET/CT. Cancer Treat Res Commun. 2021;27:100356. doi:10.1016/j.ctarc.2021.100356
36. Grillo P, Di Giuliano F, Massa R, Mercuri NB, Schirinzi T. Movement disorders in primary central nervous system lymphoma: two unreported cases and a review of literature. Neurol Sci. 2021;42(3):905–910. doi:10.1007/s10072-020-04622-9.
37. Christensen PB, Gregersen H, Almasi C. Anti-Tr/DNER antibody paraneoplastic cerebellar degeneration preceding a very late relapse of Hodgkin lymphoma after 12 years. Cerebellum Ataxias. 2021;8(1):14. doi:10.1186/s40673-021-00138-y.
38. Aggarwal P, Kavanal AJ, Singh H, Kumar R, Ranjan R, Baishya J, et al. Versatile Role of 18F-FDG PET/CT in a Patient with Pan-cerebellar Paraneoplastic Manifestation Due to Underlying Hodgkin’s Lymphoma. Nuclear Medicine and Molecular Imaging. 2021;55(6):311–4.
39. Saini V, Dhir A, Rudnick AW, Lukas J, Lizarraga KJ, Margolesky J, et al. Paraneoplastic cerebellar degeneration in diffuse large B-cell lymphoma and review of associated onconeural antibodies. Clin Lymphoma Myeloma Leuk. 2020;20(6):e336–e340. doi:10.1016/j.clml.2020.01.016.
40. Petkov R, Valkova M, Alaikov T, Guergueltcheva V, Shivarov V. Unique combination of anti-paraneoplastic antigen Ma2 antibody-positive Ophelia syndrome and opsoclonus–myoclonus syndrome in an adult Hodgkin’s lymphoma patient. Clin Exp Neuroimmunol. 2020;11(1):73–77. doi:10.1111/cen3.12540.
41. Nagarajan E, Yerram SY, Digala LP, Bollu PC. Primary central nervous system lymphoma presenting as parkinsonism with atypical MRI findings and elevated 14-3-3 protein. J Neurosci Rural Pract. 2020;11(3):492–494. doi:10.1055/s-0040-1710404.
42. Merrill S, Mauler DJ, Richter KR, Raghunathan A, Leis JF, Mrugala MM. Parkinsonism as a late presentation of lymphomatosis cerebri following high-dose chemotherapy with autologous stem cell transplantation for primary central nervous system lymphoma. J Neurol. 2020;267(8):2239–2244. doi:10.1007/s00415-020-09823-w.
43. Cao X, Xu CG, Wang NN. Paraneoplastic cerebellar degeneration: Initial presentation of mucosa-associated lymphoid tissue lymphoma in a patient with primary Sjögren’s syndrome. Chin Med J (Engl). 2020;133(8):1005–1007. doi:10.1097/CM9.0000000000000720.
44. Arratibel N, Sobejano E, Morán JC, Diaz LG, Blázquez A, Baile M, et al. A case of paraneoplastic cerebellar degeneration that preceded the diagnosis of classical Hodgkin’s lymphoma by 16 months. Am J Case Rep. 2020;21:e922342. doi:10.12659/AJCR.922342.
45. Tee TY, Khoo CS, Mohamed Ibrahim NM, Osman SS. A rare extrapyramidal manifestation in a patient with primary central nervous system lymphoma. Neurol India. 2019;67(1):297–299. doi:10.4103/0028-3886.253613.
46. Sato S, Shibahara I, Inoue Y, Hide T, Kumabe T. New radiologic findings of hypertrophic olivary degeneration in 2 patients with brainstem lymphoma. World Neurosurg. 2019;123:464–468.e1. doi:10.1016/j.wneu.2018.12.208.
47. Nevison S, Rizek P. Anti-CV2–associated paraneoplastic hemichorea secondary to diffuse large B-cell lymphoma. Can J Neurol Sci. 2019;46(4):480–481. doi:10.1017/cjn.2019.20.
48. Herrscher H, Artzner T, Coca HA, Schneider F, Proust F, Guillot M. Cerebral lymphoma presenting as a rhombencephalitis: Case report and review of the literature. Rev Neurol (Paris). 2019;175(6):412–414. doi:10.1016/j.neurol.2018.10.002.
49. Conway R, Kaluza V, Schwartz K, Chang HT. A 77-year-old woman with a right cerebellar lesion. Brain Pathol. 2019;29(4):579–580. doi:10.1111/bpa.12709.
50. Chong LL, Wong EYT, Santos-Banta SLL, Cheng CL, Tan L, Poon EYL, et al. Successful therapeutic rechallenge after a severe episode of high-dose methotrexate-induced choreoathetosis: A case report. Mol Clin Oncol. 2019;11(4):354–358. doi:10.3892/mco.2019.1922.
51. Reddy SY, Shenker JI, Bollu PC. Primary central nervous system lymphoma presenting as parkinsonism with atypical MRI findings and elevated 14-3-3 protein. Int J Adv Surg. 2019;3(1):5–7. doi:10.23937/2643-4474/1710009.
52. Zurko J, Mehta A. Association of immune-mediated cerebellitis with immune checkpoint inhibitor therapy. Mayo Clin Proc Innov Qual Outcomes. 2018;2(1):74–77. doi:10.1016/j.mayocpiqo.2017.12.002.
53. Voeller J, Ikonomidou C, Brucker J, Diamond C, Frierdich S, Patel N. Autoimmune ataxia during maintenance therapy for acute lymphoblastic leukemia. Child Neurol Open. 2018;5:2329048X18780361. doi:10.1177/2329048X18780361.
54. Khan N. Paraneoplastic cerebellar degeneration in a patient with anaplastic non-Hodgkin’s lymphoma. BMJ Case Rep. 2018;2018:bcr-2017-223613. doi:10.1136/bcr-2017-223613.
55. Ghannam M, Mansour S, Jumah F, Berry B, Beard A. Cerebellar large B-cell lymphoma: A case report. J Med Case Rep. 2018;12(1):6. doi:10.1186/s13256-017-1538-0.
56. Tran PN, Kong XT. Cytarabine-Induced Acute Cerebellar Syndrome during Hyper-CVAD Treatment for B-Cell Acute Lymphoblastic Leukemia. Case Reports in Neurology. 2017;9(1):114-120. doi:10.1159/000468921.
57. Toyota T, Sennari Y, Okada K, Kawahara A, Tsukada J, Shimajiri S, et al. Myoclonia continua in primary CNS natural killer/T-cell lymphoma, nasal type. Neurology. 2017;88(3):329–330. doi:10.1212/WNL.0000000000003539.
58. Güngör S, Kiliç B, Arslan M, Özgen U, Dalmau J. Hodgkin’s lymphoma associated with paraneoplastic cerebellar degeneration in children: a case report and review of the literature. Child Nerv Syst. 2017;33(3):509–512. doi:10.1007/s00381-016-3273-4.
59. Esplin NE, Stelzer JW, Legare TB, Ali SK. Difficult to treat focal, stiff person syndrome of the left upper extremity. Case Rep Neurol Med. 2017;2017:2580620. doi:10.1155/2017/2580620.
60. Borellini L, Lanfranconi S, Bonato S, Trezzi I, Franco G, Torretta L, et al. Progressive encephalomyelitis with rigidity and myoclonus associated with anti-GlyR antibodies and Hodgkin’s lymphoma: A case report. Front Neurol. 2017;8:401. doi:10.3389/fneur.2017.00401.
61. Abruzzese E, Trawinska MM, Gaspardone A, Morocutti A, De Fabritiis P. Hodgkin's lymphoma in a man with dilated cardiomyopathy and paraneoplastic ataxia: A therapeutical challenge. Hematology Reports. 2017;9(2):50–52. doi:10.4081/hr.2017.7053.
62. Zahra K, Bouneb R, Mahjoub M, Zaghouani H, Zaier M, Ben Youssef Y, et al. Acute methotrexate neurotoxicity revealed by a cerebellar syndrome. Rev Neurol (Paris). 2016;172(12):791–793. doi:10.1016/j.neurol.2016.08.005.
63. Shimatani Y, Nakano Y, Tsuyama N, Murayama S, Oki R, Miyamoto R, et al. Extranodal NK/T-cell lymphoma, nasal type, manifesting as rapidly progressive dementia without any mass or enhancing brain lesion. Neuropathology. 2016;36(5):456–463. doi:10.1111/neup.12285.
64. Hatcher-Martin JM, Factor SA. Freezing of gait: A rare delayed complication of whole brain radiation. Parkinsonism Relat Disord. 2016;29:129–130. doi:10.1016/j.parkreldis.2016.06.018.
65. Grubbs J Jr, Trobe JD, Fisher-Hubbard A. Opsoclonus-myoclonus syndrome in primary central nervous system lymphoma. J Neuroophthalmol. 2016;36(4):408–11. doi:10.1097/WNO.0000000000000414.
66. Gray DA, Foo D. Reversible myoclonus, asterixis, and tremor associated with high-dose trimethoprim-sulfamethoxazole: a case report. J Spinal Cord Med. 2016;39(1):115–117. doi:10.1179/2045772315Y.0000000045.
67. Chepovetsky J, Duffield AS, Pu JJ. Paraneoplastic cerebellar degeneration as an early sign of classical Hodgkin lymphoma. Ann Hematol. 2016;95(3):511–513. doi:10.1007/s00277-015-2573-8.
68. Yamamoto J, Kitagawa T, Akiba D, Nishizawa S. 5-aminolevulinic acid-induced fluorescence in cerebellar primary central nervous system lymphoma: a case report and literature review. Turk Neurosurg. 2015;25(5):796–800. doi:10.5137/1019-5149.JTN.10496-14.1.
69. Sáenz-Farret M, González-Aguilar A, Estefanía Sánchez-Correa TE, Camji J, Mícheli F. Primary central nervous system lymphoma as a cause of hemichorea in a patient with acquired immunodeficiency syndrome: A case report and a review of the literature. Basal Ganglia. 2015;5(2–3):59–62. doi:10.1016/j.baga.2015.05.002.
70. Manir KS, Basu S, Bhowmik R, Banerjee D. Paraneoplastic cerebellar dysfunction in Hodgkin's lymphoma. Clin Cancer Investig J. 2015;4(6):766–768. doi:10.4103/2278-0513.168097.
71. Briongos-Figuero LS, Gómez-Traveso T, Pérez-Castrillón JL. T-cell primary leptomeningeal lymphoma in cerebellopontine angle. BMJ Case Rep. 2015;2015:bcr2014208602. doi:10.1136/bcr-2014-208602.
72. Reddy A, Santhosh S, Mittal B, Bhattacharya A, Sharma S. Isolated cerebellar hypermetabolism on FDG PET in a case of remitted primary breast lymphoma. Indian J Nucl Med. 2014;29(1):55–56. doi:10.4103/0972-3919.125783.
73. Noda K, Hattori N, Okuma Y. Primary central nervous system lymphoma presenting as choreoathetosis. BMJ Case Rep. 2014;2014:bcr2014204814. doi:10.1136/bcr-2014-204814.
74. George J, Gourisankar PT, Radhakrishnan S, Kumar AA, Kannan RR, Bindhu MR. A seventy-year-old man with intractable vomiting, Parkinsonism, memory loss and ptosis. Ann Indian Acad Neurol. 2014;17(2):155–160. doi:10.4103/0972-2327.132645.
75. Rigual D, Qiu J, Fenstermaker RA, Fabiano AJ. Tumoral Bing–Neel syndrome presenting as a cerebellar mass. Clin Neurol Neurosurg. 2013;115(6):823–826. doi:10.1016/j.clineuro.2012.09.027.
76. Rakocevic G, Hussain A. Stiff person syndrome improvement with chemotherapy in a patient with cutaneous T-cell lymphoma. Muscle Nerve. 2013;47(6):938–939. doi:10.1002/mus.23778.
77. Pandit L, Raghotham A, Chickabasaviah Y, Khandige G, Shetty R. Neoplastic parkinsonism: An illustrative case report. Ann Indian Acad Neurol. 2013;16(3):437–439. doi:10.4103/0972-2327.116933.
78. Lakshmaiah KC, Viveka BK, Anil Kumar N, Saini ML, Sinha S, Saini KS. Gastric diffuse large B cell lymphoma presenting as paraneoplastic cerebellar degeneration: Case report and review of literature. J Egypt Natl Canc Inst. 2013;25(4):231–235. doi:10.1016/j.jnci.2013.05.006.
79. Cachia D, Izzy S, Smith T, Ionete C. A rare presentation of hypertrophic olivary degeneration secondary to primary central nervous system lymphoma. JAMA Neurol. 2013;70(9):1192–1193. doi:10.1001/jamaneurol.2013.364.
80. Tsai T, McGrath R. Lymphoma, thymoma and the wooden man: stiff-person syndrome post-thymoma excision and non-Hodgkin lymphoma remission. Intern Med J. 2012;42(2):205–207. doi:10.1111/j.1445-5994.2011.02557.x.
81. Suri V, Khan NI, Jadhao N, Gupta R. Paraneoplastic cerebellar degeneration in Hodgkin’s lymphoma. Ann Indian Acad Neurol. 2012;15(3):205–207. doi:10.4103/0972-2327.99700.
82. Shimazu Y, Minakawa EN, Nishikori M, Ihara M, Hashi Y, Matsuyama H, et al. A case of follicular lymphoma associated with paraneoplastic cerebellar degeneration. Intern Med. 2012;51(11):1387–1392. doi:10.2169/internalmedicine.51.6901.
83. Kim HS, Jung CO, Jeon HR, Sung LH. Rehabilitation for ataxia following chemotherapy for Burkitt lymphoma involving the rectum. Ann Rehabil Med. 2012;36(4):578–583. doi:10.5535/arm.2012.36.4.578.
84. Benz R, Viecelli A, Taverna C, Schelosky L. Paroxysmal non-kinesigenic dyskinesia due to spinal cord infiltration of low-grade B-cell non-Hodgkin's lymphoma. Ann Hematol. 2012;91(3):463–465. doi:10.1007/s00277-011-1327-3.
85. Ishihara S, Kano O, Ikeda K, Shimokawa R, Kawabe K, Iwasaki Y. Clinicoradiological changes of brain NK/T cell lymphoma manifesting pure akinesia: a case report. BMC Neurol. 2011;11:137. doi:10.1186/1471-2377-11-137.
86. Hemmaway C, Laverse E, Nicholas M, Nagy Z. Cerebellar Cladophialophora bantiana infection in a patient with marginal zone lymphoma treated with immunochemotherapy including rituximab. Br J Haematol. 2011;154(4):423. doi:10.1111/j.1365-2141.2011.08736.x.
87. Sanz A, Montero Á, Salas C, Amaya E, Rodríguez G, Magallón R, et al. Cerebellar involvement in Hodgkin’s lymphoma: an atypical site of relapse. Clin Transl Oncol. 2010;12(6):453–5. doi:10.1007/s12094-010-0534-4.
88. Pless ML, Chen YB, Copen WA, Frosch MP. Case records of the Massachusetts General Hospital. Case 9-2010. A 37-year-old woman with paresthesias and ataxia. N Engl J Med. 2010;362(12):1129–1138. doi:10.1056/NEJMcpc1001043.
89. Milia A, Pilia G, Mascia MG, Manconi FM. Treatment-induced leukoencephalopathy in primary CNS lymphoma presenting as lower body parkinsonism. J Neurooncol. 2010;100(2):281–283. doi:10.1007/s11060-010-0159-9.
90. Lin CM, Hong K. Cerebral infratentorial large B-cell lymphoma presenting as parkinsonism. Tohoku J Exp Med. 2010;220(3):187–190. doi:10.1620/tjem.220.187
91. Rodis DG, Liatsos GD, Moulakakis A, Pirounaki M, Tasidou A. Paraneoplastic cerebellar degeneration: Initial presentation in a patient with anaplastic T-cell lymphoma, associated with ichthyosiform cutaneous lesions. Leuk Lymphoma. 2009;50(8):1369–71.
92. Necioğlu Örken D, Yıldırmak Y, Kenangil G, Kandıralıoğlu N, Forta H, Çelik M. Intrathecal methotrexate-induced acute chorea. J Pediatr Hematol Oncol. 2009;31(1):57–58. doi:10.1097/MPH.0b013e3181876052.
93. Kolbaske S, Grossmann A, Benecke R, Wittstock M. Progressive gait ataxia and intention tremor in a case of Bing–Neel syndrome. Journal of Neurology. 2009;256(8):1366–1368. doi:10.1007/s00415-009-5107-5.
94. Karmon Y, Inbar E, Cordoba M, Gadoth N. Paraneoplastic cerebellar degeneration mimicking acute post-infectious cerebellitis. Cerebellum. 2009;8(4):441–444. doi:10.1007/s12311-009-0108-2.
95. de Lima MA, Maradei S, Maranhão-Filho P. Cyclosporine-induced parkinsonism. Journal of Neurology. 2009;256(4):674–675. doi:10.1007/s00415-009-0137-6.
96. Bota DA, Dafer RM. Acute methotrexate neurotoxicity with choreiform movements and focal neurological deficits: A case report. Southern Medical Journal. 2009;102(10):1071–1074.
97. Rizzo R, Marino S, Gulisano M, Robertson MM. The successful use of ondansetron in a boy with both leukemia and Tourette syndrome. Journal of Child Neurology. 2008;23(1):108–111. doi:10.1177/0883073807307085.
98. Gofton TE, Macdonald DR, Tartaglia MC, Lee DH, Megyesi JF, Hammond RR. Ataxia and diplopia in a patient with chronic lymphocytic leukemia. Canadian Journal of Neurological Sciences. 2008;35(2):243–246. doi:10.1017/S0317167100008714.
99. Gallagher DA, Schott JM, Childerhouse A, Wilhelm T, Gale AN, Schrag A. Reversible “applause sign” secondary to diffuse large B-cell lymphoma. Mov Disord. 2008;23(16):2426–2428. doi:10.1002/mds.22240.
100. Williams-Gray CH, Aliyu SH, Lever AM, Dean AF, Lennox GG. Reversible parkinsonism in a patient with progressive multifocal leucoencephalopathy. J Neurol Neurosurg Psychiatry. 2007;78(4):408–410. doi:10.1136/jnnp.2006.103259.
101. Tie J, Forgeson G, McNabb A, Roebert J. Seek and ye shall find: Hodgkin's lymphoma presenting as paraneoplastic cerebellar degeneration. N Z Med J. 2007;120(1252):U2495.
102. Razzak A, Shields M, Allsup D. Autoimmune paraneoplastic cerebellar degeneration secondary to a composite lymphoma. Leuk Lymphoma. 2007;48(7):1456–1458. doi:10.1080/10428190701387047.
103. Foy J, Primrose W, Mackenzie J. Primary cerebral lymphoma presenting with Parkinsonism. Scott Med J. 2007;52(1):55. doi:10.1258/rsmsmj.52.1.55.
104. Ypma PF, Wijermans PW, Koppen H, Sillevis Smitt PA. Paraneoplastic cerebellar degeneration preceding the diagnosis of Hodgkin’s lymphoma. Neth J Med. 2006;64(7):243–247.
105. Geromin A, Candoni A, Marcon G, Ferrari S, Sperotto A, de Luca S, et al. Paraneoplastic cerebellar degeneration associated with anti-neuronal anti-Tr antibodies in a patient with Hodgkin’s disease. Leuk Lymphoma. 2006;47(9):1960–1963. doi:10.1080/10428190600678082.
106. Denison DJ, Alghzaly AA. Busulfan induced myoclonus. Saudi Medical Journal. 2006;27(4):557–558.
107. Tan J, Goh B, Tambyah P, Wilder-Smith E. Paraneoplastic progressive supranuclear palsy syndrome in a patient with B-cell lymphoma. Parkinsonism Relat Disord. 2005;11(3):187–91. DOI: 0.1016/j.parkreldis.2004.09.003.
108. Kumar A, Lajara-Nanson WA, Neilson RW Jr. Paraneoplastic opsoclonus-myoclonus syndrome: Initial presentation of non-Hodgkin’s lymphoma. J Neurooncol. 2005;73(1):43–5. doi:10.1007/s11060-004-5198-5.
109. Hengstman GJD, van Rossum MM, van der Kerkhof PCM, Bloem BR. Chorea due to mycosis fungoides metastasis. J Neurooncol. 2005;73(1):87–88. doi:10.1007/s11060-004-5199-4.
110. Batchelor TT, Buchbinder BR, Harris NL. Case records of the Massachusetts General Hospital. Case 1-2005: A 35-year-old woman with difficulty walking, headache, and nausea. N Engl J Med. 2005;352(2):185–94. doi:10.1056/NEJMcpc049024.
111. Jundt F, Lempert T, Dörken B, Pezzutto A. Trimethoprim-sulfamethoxazole exacerbates posthypoxic action myoclonus in a patient with suspicion of Pneumocystis jiroveci infection. Infection. 2004;32(3):176-178. doi:10.1007/s15010-004-3011-6.
112. Wiener V, Honnorat J, Pandolfo M, Kentos A, Manto MU. Myorhythmia associated with Hodgkin’s lymphoma. J Neurol. 2003;250(11):1382–1384. doi:10.1007/s00415-003-0203-4.
113. Tan EK, Chan LL, Auchus AP, Wong MC. Reversible choreoathetosis in primary cerebral lymphoma: Clinicoradiologic correlation. Eur Neurol. 2003;50(1):53–54. doi:10.1159/000070859.
114. Samii A, Dahlen DD, Spence AM, Maronian NC, Kraus EE, Lennon VA. Paraneoplastic movement disorder in a patient with non-Hodgkin’s lymphoma and CRMP-5 autoantibody. Movement Disorders. 2003;18(12):1556–1558. doi:10.1002/mds.10616
115. Rollnik JD, Winkler T, Ganser A. A case of symptomatic paroxysmal kinesigenic dyskinesia with primary central nervous system lymphoma. Der Nervenarzt. 2003;74(4):362–365. doi: 10.1007/s00115-002-1448-3
116. Gottesman R, Höke A. Clinical cases in neurology from Johns Hopkins – Case 3: 44-year-old man with fever, headache, confusion and ataxia. MedGenMed. 2003;5(3). Available from: https://www.medscape.com/viewarticle/460254.
117. Ferhanoğlu B, Öngören S, Ar CM, Uzel B, Forta H, Necioglu D. Intrathecal methotrexate-induced acute cerebellar syndrome. Annals of Hematology. 2003;82(4):241–243. doi:10.1007/s00277-003-0618-0.
118. Chuang C, Constantino A, Balmaceda C, Eidelberg D, Frucht SJ. Chemotherapy-induced parkinsonism responsive to levodopa: An underrecognized entity. Movement Disorders. 2003;18(3):328-331. doi:10.1002/mds.10344
119. Sheen VL, Asimakopoulos F, Heyman E, Henderson G, Feske SK. Hemichorea as a presentation of recurrent non-Hodgkin’s lymphoma. J Neurol. 2002;249(12):1746–8. doi:10.1007/s00415-002-0912-0.
120. Alderson LM, Delalle I. Case records of the Massachusetts General Hospital. Weekly clinicopathological exercises. Case 10-2002. A 52-year-old woman with recurrent unsteadiness, slurred speech, and fatigue. New England Journal of Medicine.2002;346(13):1009–1015.doi:10.1056/NEJMcpc020010.
121. Yeshurun M, Dupuch KM. Acute cerebellar syndrome following intermediate-dose cytarabine. Br J Haematol. 2001;113(4):846. doi:10.1046/j.1365-2141.2001.02771.x.
122. Sánchez-Guerra M, Cerezal L, Leno C, Díez C, Figols J, Berciano J. Primary brain lymphoma presenting as Parkinson's disease. Neuroradiology. 2001;43(1):36-40. doi:10.1007/s002340000480.
123. Rauch R, Jüngert J, Rupprecht T, Greil J. Torticollis revealing as a symptom of acute lymphoblastic leukaemia in a fourteen-month-old girl. Acta Paediatr. 2001;90(5):587–588. doi:10.1111/j.1651-2227.2001.tb00805.x.
124. Nuti A, Ceravolo R, Salvetti S, Gambaccini G, Bonuccelli U, Capochiani E. Paraneoplastic choreic syndrome during non-Hodgkin’s lymphoma. Mov Disord. 2000;15(2):350–352. doi:10.1002/1531-8257(200003)15:2<350::AID-MDS1026>3.0.CO;2-A.
125. Emir S, Kutluk MT, Göğüş S, Büyükpamukçu M. Paraneoplastic cerebellar degeneration and Horner syndrome: Association of two uncommon findings in a child with Hodgkin disease. Journal of Pediatric Hematology/Oncology. 2000;22(2):158-161. doi:10.1097/00043426-200003000-00015.
126. Pramstaller PP, Salerno A, Bhatia K, Prugger M, Marsden CD. Primary central nervous system lymphoma presenting with a parkinsonian syndrome of pure akinesia. J Neurol. 1999;246(10):934–8. doi:10.1007/s004150050479.
127. Benzing T, Rump LC, Kaiser R, Peter HH. Paraneoplastic cerebellar degeneration in Hodgkin's disease. Dtsch Med Wochenschr. 1998;123(16):493–6. doi: 10.1055/s-2007-1024000.
128. Batchelor TT, Platten M, Palmer-Toy DE, Hunter GJ, Lev MH, Dalmau J, et al. Chorea as a paraneoplastic complication of Hodgkin's disease. Journal of Neuro-Oncology. 1998;36(2):185–190. DOI: https://doi.org/10.1023/A:1005860103173.
129. Wyllie AR, Bayliff CD, Kovacs MJ. Myoclonus due to chlorambucil in two adults with lymphoma. Ann Pharmacother. 1997;31(2):171-4. doi:10.1177/106002809703100208.
130. Gambardella A, Zappia M, Valentino P, Aguglia U, Fera F, Pardatscher K, et al. Action palatal tremor in a patient with primary intestinal lymphoma. Mov Disord. 1997;12(5):794–7. doi:10.1002/mds.870120530.
131. Eder JP, Nguyen PL. A 67-year-old woman with a progressive movement disorder and a left–upper-quadrant mass. N Engl J Med. 1997;337(2):115–22. doi:10.1056/NEJM199707103370208.
132. Symonds RP, Hogg RB, Bone I. Paraneoplastic neurological syndromes associated with lymphomas. Leuk Lymphoma. 1994;15(5–6):487–90. doi:10.3109/10428199409049752.
133. Krishnan K, Bockenstedt P. Paraneoplastic cerebellar degeneration: a rare presentation of Hodgkin's disease. Clin Lab Haematol. 1994;16(4):359–62.
134. Howell SJL, Sagar HJ. A progressive parkinsonian syndrome developing after chemotherapy and radiotherapy for non-Hodgkin's lymphoma. Mov Disord. 1994;9(3):373–5. doi:10.1002/mds.870090325.
135. Kay CL, Davies-Jones GA, Singal R, Winfield DA. Paraneoplastic opsoclonus–myoclonus in Hodgkin’s disease. J Neurol Neurosurg Psychiatry. 1993;56(7):831–2. doi:10.1136/jnnp.56.7.831.
136. Topcu M, Gucuyener K, Topaloglu H, Renda Y, Akyuz C, Kale G. Paraneoplastic syndrome manifesting as chronic cerebellar ataxia in a child with Hodgkin disease. J Pediatr. 1992;120(2 Pt 1):275–7. doi:10.1016/S0022-3476(05)80329-1.
137. Schiff DE, Ortega JA. Chorea, eosinophilia, and lupus anticoagulant associated with acute lymphoblastic leukemia. Pediatric Neurology. 1992;8(6):466–468. doi:10.1016/0887-8994(92)90011-M.
138. Oliveras C, D’Olhaberriague L, Garcia J, Matias-Guiu X. Parkinsonism as first manifestation of lymphomatoid granulomatosis. Journal of Neurology, Neurosurgery & Psychiatry. 1988;51(7):999–1001. doi:10.1136/jnnp.51.7.999.
139. Poewe W, Kleedorfer B, Willeit J, Gerstenbrand F. Primary CNS lymphoma presenting as a choreic movement disorder followed by segmental dystonia. Mov Disord. 1988;3(4):320–5. doi:10.1002/mds.870030404.
140. Ascher DP, Delaney RA. Acute dystonia from etoposide. Drug Intelligence and Clinical Pharmacy. 1988;22(1):41–42. doi:10.1177/106002808802200109.
141. Gherardi R, Roualdes B, Fleury J, Prost C, Poirier J, Degos JD. Parkinsonian syndrome and central nervous system lymphoma involving the substantia nigra. Acta Neuropathol. 1985;65(3–4):338–43. doi:10.1007/BF00687019.
142. Bejar JM. Compazine-induced dyskinesia in a 14-month-old boy. Clinical Neuropharmacology. 1984;7(2):171–172. doi:10.1097/00002826-198406000-00009.
143. Sinniah D, Lin HP. Extrapyramidal tract syndrome following intensive MOPP therapy for stage IV-B Hodgkin’s disease in childhood. Cancer. 1979;43(5):1587–9. doi:10.1002/1097-0142(197905)43:5<1587::AID-CNCR2820430530>3.0.CO;2-J.
144. Bean SC, Ladisch S. Chorea associated with a subdural hematoma in a child with leukemia. J Pediatr. 1977;90(2):255–256. doi:10.1016/S0022-3476(77)80888-7.
145. Horwich L, Buxton PH, Ryan GMS. Cerebellar degeneration with Hodgkin’s disease. J Neurol Neurosurg Psychiatry. 1966;29(1):45–51. doi:10.1136/jnnp.29.1.45.
