# Supplementary material for: Spectrum of Movement Disorders in Hematological Malignancies: A Comprehensive Systematic Review of Clinical Phenotypes, Mechanisms, and Outcomes
Source: Tremor Other Hyperkinet Mov (N Y). 2026 Mar 19;16:18. doi: 10.5334/tohm.1147 (PMC13004057; doi:10.5334/tohm.1147)
Supplement: Supplementary File. — Supplementary Tables 1 to 4. [file tohm-16-1-1147-s1.zip › tohm-1147_garg-s1/Supplementary Table 2.docx]

**Supplementary Table 2: Structured Dataset Summarizing Demographics, Hematological Features, CNS Involvement, Movement Disorder Phenomenology, Ancillary Findings, Mechanisms, and Outcomes in 78 Reported Cases of Myeloid Hematological Malignancies**

| **Author/Year** | **Country** | **Age/Sex** | **Myeloid Hematological Category** | **Specific Diagnosis** | **Disease Stage** | **Molecular Markers** | **CNS Involvement** | **Movement Disorder Type/Ataxia** | **Phenomenology** | **Timing of Onset** | **Associated Neurologic Manifestations** | **Paraneoplastic Antibodies** | **Drug-Induced Neurotoxicity** | **CSF Findings** | **Neuroimaging Findings** | **PET-CT Findings** | **Hematology Treatment** | **Targeted Therapy** | **HSCT** | **Neurology Treatment** | **Neurological Outcome** | **Hematological Outcome** | **Proposed Mechanism** | **Follow-up Duration** |
| --- | --- | --- | --- | --- | --- | --- | --- | --- | --- | --- | --- | --- | --- | --- | --- | --- | --- | --- | --- | --- | --- | --- | --- | --- |
| Zhao et al., 2025 | China | 13/F | Acute Myeloid Leukemia | AML-M2 | Post–two allo-HSCT (2y & 5m earlier) | Not reported | No leukemic CNS relapse; CSF normal; drug toxicity | Extrapyramidal symptoms / dystonia | Tongue protrusion, blinking, grimacing, dystonia, altered mental status, irritability, lethargy | After prolonged metronidazole exposure | Altered mental status, dysarthria, dysphagia, ophthalmoplegia, urinary retention, neuropathy, absent reflexes | None | Metronidazole neurotoxicity | Normal | Symmetric T2/FLAIR/DWI hyperintensities in dentate nuclei, splenium, basal ganglia, thalamus, midbrain, pons, WM | Not reported | Post-HSCT status | None | HSCT x2 | Stop metronidazole + Vitamin B1 | Persistent extrapyramidal symptoms | Death due to GVHD | Metronidazole toxicity + hepatic impairment | 1 month |
| Vijayaraghavan et al., 2025 | India | 17/M | Myeloid malignancy (remote APML history) | APML (childhood; long-term remission) | Complete remission | Not reported | No CNS leukemia; autoimmune CNS involvement | Polyminimyoclonus / neuromyotonia | Akinetic mutism, fasciculations, cramps, irritability, insomnia, exfoliative dermatitis | Gradual onset after febrile illness | Hypotonia, areflexia, autonomic dysfunction, exfoliative skin lesions | Anti-CASPR2 + Anti-LGI1 positive | None | Not reported | Bilateral symmetric basal ganglia hyperintensities (T2/FLAIR) | Not reported | None (APML in remission) | None | No HSCT | Pulse steroids + monthly IVIG + Tenofovir | Complete recovery | APML stable in remission | Dual CASPR2/LGI1 Morvan syndrome triggered by HBV | 3 months follow-up |
| Sodemann et al., 2025 | Denmark | 82/F | Myeloproliferative Neoplasm (MPN) | Polycythemia Vera (PV) | Newly diagnosed | JAK2 V617F+ (82% allele burden), Low EPO | No CNS lesion; hyperviscosity-related chorea | Hemichorea | Left-sided chorea, tongue chorea, dysarthria, ataxia, behavioral changes | Acute onset over hours | Ataxia, dysarthria, disinhibition, broad-based gait | None | None | Normal CSF | CT normal; MRI only chronic ischemia | Not reported | Phlebotomy + Aspirin + Hydroxyurea | None | No | No specific neurotherapy; hematologic treatment improved symptoms | Complete recovery | Good hematologic response | PV hyperviscosity causing BG hypoperfusion | Few months follow-up |
| Sassani et al., 2025 | UK | 85/F | Myelodysplastic Syndrome (MDS) | JAK2V617F-positive 5q-MDS | Newly diagnosed | JAK2 V617F mutation; Megakaryocytic dysplasia | No CNS infiltration; cortical infarction | Palatal-lingual-facial myoclonus | Involuntary palatal/tongue movements, facial twitching, jerky speech, pulsatile throat sensation | Acute onset on awakening | No major associated neurologic signs | None | None | Not reported | Right primary motor cortex infarct; small occipital infarct | Not reported | Lenalidomide; Apixaban + Clopidogrel | None | No | No antiseizure therapy; spontaneous resolution | Complete neurological recovery | Hematologic stabilization | Cortical infarct due to JAK2 prothrombotic state | Short inpatient follow-up |
| Saibaba et al., 2025 | India | 51/M | Myeloproliferative Neoplasm | JAK2+ Polycythemia Vera with Secondary Myelofibrosis | Secondary MF; longstanding PV | JAK2 V617F; hypercellular marrow; erythroid+megakaryocytic hyperplasia | No CNS infiltration; CANVAS syndrome | Cerebellar ataxia + neuropathy + bilateral vestibular areflexia | Gait ataxia, oscillopsia, sensory ataxia, nystagmus, dysarthria | Progressive over 6–7 years | Sensory axonal neuropathy; vestibulopathy; dysarthria | Paraneoplastic panel negative | Thalidomide-induced neuropathy | Normal CSF | Cerebellar atrophy (vermis, paravermis) | Splenomegaly on CT | Thalidomide + Prednisolone (past); thalidomide restarted | None | No | Vestibular rehab + amitriptyline | Persistent imbalance | Progressive PV/MF; death from pneumonia/sepsis | CANVAS + thalidomide neurotoxicity + diabetes + alcohol | 1.5 years |
| Ma et al., 2025 | China | 38/M | Acute Myeloid Leukemia (High-risk AML) | AML with WT1+, FLT3-TKD, IKZF1, RUNX1, JAK1, ASXL2, SETD2 mutations | Induction → partial response → HiDAC intensification | WT1+, FLT3-TKD, IKZF1, RUNX1, JAK1, ASXL2, SETD2; CD34+, CD117+, MPO+ | No CNS toxicity except dysphonia; no cerebellar signs | Dysphonia (hoarseness) | Soft, hoarse voice starting Day 3 of HiDAC | Day 3 after HiDAC | No ataxia, dysarthria, nystagmus, dysmetria | None | High-dose cytarabine toxicity | Laryngoscopy normal | No neuroimaging abnormalities | Not performed | Venetoclax + Azacitidine → MA → HiDAC | None | No | Supportive care only | Resolved each time after stopping HiDAC | AML achieved complete remission | HiDAC dose-dependent neurotoxicity | Follow-up across 3 HiDAC cycles |
| Lee et al., 2025 | South Korea | 59/M | Myeloproliferative Neoplasm (MPN) | JAK2-positive Polycythemia Vera | Newly diagnosed | JAK2 V617F+; hypercellular marrow; ↑RBC/Hb/Hct/WBC/Platelets | No CNS leukemia; functional basal ganglia injury | Hemichorea-ballism (left-sided) | Irregular choreoballistic limb movements; worsened with gait | Sudden onset (1 day before admission) | No dystonia/ataxia/parkinsonism; normal cognition | None | None | Normal metabolic/genetic tests | Initial MRI normal → Repeat: right GP DWI+, FLAIR/T1 enhancement | SPECT: Thalamic hyperperfusion | Phlebotomy + Aspirin + Hydroxyurea | None | No | Tetrabenazine 62.5→25 mg | Near-complete resolution at 6 months | Good hematologic control | PV-related microvascular hypoperfusion | 6 months |
| Harrison et al., 2025 | USA | 75/F (Asian) | Myeloproliferative Neoplasm | Essential Thrombocythemia (JAK2V617F 24%) | Long-standing ET (3 years) | JAK2V617F+ (24%); Platelets 359k (prior 806k) | No CNS leukemia; hemorrhage-related | Hemichorea-hemiballismus | Right-sided chorea/ballism of arm>leg; stress-aggravated; momentarily suppressible | 5 days before presentation | Mild hyporeflexia; no sensory/speech deficits | None | None | Normal metabolic/infectious workup | Subacute left basal ganglia hemorrhage; 3‑month evolution seen | Not done | Hydroxyurea dose increased; platelet control | None | No | Risperidone 0.25 mg BID → 0.5 mg BID | Recurrence on taper; resolved with restart | ET controlled; platelets improved | Hemorrhage from ET-related vascular fragility | 3+ months |
| Rusche et al., 2024 | Switzerland | 61/M | Acute Myeloid Leukemia | NPM1‑mutated AML | Post‑allogeneic HSCT (14 months, remission) | NPM1 mutation; post‑HSCT immune dysregula楴湯(一 | No CNS leukemia; autoimmune encephalitis | Myoclonus → generalized super‑refractory status epilepticus | Persistent rhythmic myoclonus (0.5–1 Hz) left leg/abdomen → spread to arm | 5 days before admission | Confusion → drowsiness → stupor; focal EEG slowing | Anti‑GABAA receptor antibodies | None | Normal CSF (cells & protein normal; PCR negative) | Serial MRI: multifocal cortico‑subcortical FLAIR lesions; no diffusion restriction | Not done | No active leukemia therapy | None | Allogeneic HSCT (14 months prior) | Prednisolone, plasmapheresis, IVIG, rituximab, thiopental coma | Ambulatory with mild deficits at follow‑up | AML in remission | Post‑HSCT autoimmune encephalitis | 185‑day MRI follow‑up |
| Rocha-Cadman et al., 2024 | USA | 54/M | Myeloproliferative Neoplasm (Primary Myelofibrosis, JAK2−) | Primary Myelofibrosis → Allogeneic HSCT (HLA-matched sibling) | Post-HSCT complications | JAK2−; hepatosplenomegaly; CMV/EBV/Klebsiella/Enterobacter infections | No CNS leukemia; CNS toxoplasmosis | Hemichorea–hemiballismus (left-sided) | Violent flinging L arm; hyperkinetic gait difficulty; persistent | Acute onset with altered mental status and unsteady gait | Confusion, hallucinations, delirium, weakness | None | None | CSF PCR positive for T. gondii | MRI: ring-enhancing lesions in thalami, basal ganglia, frontal lobes; edema | Not done | HSCT conditioning (busulfan/melphalan/fludarabine); antimicrobials | None | Yes (allogeneic HSCT) | Clindamycin + pyrimethamine + leucovorin → later sulfadiazine regimen; Botox | Persistent chronic hemiballismus | HSCT engrafted; ongoing complications | Toxoplasma basal ganglia dysfunction | ~1 year |
| Butnariu et al., 2024 | Romania | 58/M | Myeloproliferative Neoplasm | JAK2V617F-positive pre-fibrotic Primary Myelofibrosis | Long-standing PMF (17 years) | JAK2V617F homozygous; severe splenomegaly; hypercellular marrow | No CNS infiltration; neurodegeneration + microangiopathy | Generalized chorea | Orofacial/limb chorea; intermittent; suppressible; worsened over 2 years | Progressive over 2 years | Cognitive decline, hypersomnia, depression, psychosis, suicidal behavior | None | None | LP not done (INR high) | MRI: fronto-parietal-insular atrophy; mild cerebellar atrophy; microangiopathy | Not done | Hydroxyurea restarted; folic acid; psychiatric treatment | None | No | Hydroxyurea + sertraline + memantine | Persistent cognitive deficits | Hematologic stability | JAK2-mediated neuroinflammation / microangiopathy | 10 months |
| Takahashi et al., 2023 | Japan | 44/F | Acute Myeloid Leukemia (AML M2) | AML → Allogeneic Cord Blood HSCT | Post-HSCT (11–30 months) | HLA mismatch 6/8; Tac/MMF/PSL prophylaxis | No CNS leukemia; chronic CNS–GVHD suspected | Cerebellar ataxia | Ataxic speech, gait ataxia, limb/truncal ataxia, downbeat/gaze-evoked nystagmus | Onset 11 months after HSCT | Numbness, diplopia, distal sensory loss, head tremors | Anti-ganglioside IgG (GM1, GD1a/b, GD3) positive | None | CSF: ↑protein 72 mg/dL; ↑IgG index 1.05; no cells; viral PCR– | MRI normal → later progressive cerebellar atrophy; SPECT hypoperfusion | Not done | Post-HSCT immunosuppression | Tacrolimus + MMF + Prednisolone | Yes (cord blood allogeneic HSCT) | mPSL pulses, Plasma Exchange, IVIG, Rituximab | Progressive worsening; bedridden at 30 months | AML in remission | CNS–GVHD (T-cell immune injury/microvasculitis) | 30 months follow-up |
| Shpilyukova et al., 2023 | Russia | 69/M | Myeloproliferative Neoplasm | Polycythemia Vera (JAK2V617F+) | Chronic untreated PV | JAK2 V617F; erythrocytosis, leukocytosis, thrombocytosis | No CNS lesion; mild cortical/subcortical atrophy | Generalized chorea | Orolingual and limb chorea, tongue biting, facial grimacing, hyperkinesia | Onset 1 year before presentation | Mild cognitive impairment, emotional dysregulation, memory loss | ANA mildly positive; autoimmune workup negative | None | No CSF (LP not done) | MRI: mild atrophy; no basal ganglia lesions | Not done | Phlebotomy + Hydroxyurea | None | No HSCT | Hydroxyurea + phlebotomy | Cognitive impairment persisted, non-progressive | Hematologic remission | Hyperviscosity + JAK2 inflammatory mechanism | 1 year |
| Hong et al., 2023 | South Korea | 88/F | Myeloproliferative Neoplasm | Polycythemia Vera (JAK2+) | Newly diagnosed | JAK2 V617F+; Hb 18.6 g/dL; Plt 520k; leukocytosis | No CNS lesion; functional hypermetabolic striatal involvement | Generalized chorea | Face → neck → limbs hyperkinesia; continuous chorea | Acute onset | No cognitive/speech deficits | None | None | Normal metabolic labs | MRI: atrophy + leukoaraiosis; no BG lesion | FDG-PET: putamen + precentral hypermetabolism → resolved | Phlebotomy x2 + Aspirin | None | No | Clonazepam 0.25 mg BID → TID | No recurrence at 3 months | Hematologic improvement | PV hyperviscosity → striatal hypermetabolism | 3 months |
| Wen et al., 2021 | China | 62/M | Myeloproliferative Neoplasm | Polycythemia Vera (JAK2 V617F+) | Long-standing PV (4 years) | JAK2 V617F+; RBC 7.31x10^12/L; Hct 0.678; Hb 183 g/L; WBC 15.56x10^9/L; Plt 256x10^9/L | No CNS leukemia; cerebellar venous thrombosis | Cerebellar ataxia | Vertigo, vomiting, limb/gait ataxia, mild dysarthria | Acute onset (<24 hours) | Lethargy; no cranial nerve deficits | None | None | CSF: OP 165 mmH2O; protein 106.8 mg/dL; normal glucose/chloride/cell count | MRI: mass-like left cerebellar lesion; edema; hemorrhagic components; dilated veins | CT: low density + hemorrhage; MRV normal; DSA: absent left transtentorial sinus | Hydroxyurea (chronic therapy) | None | No | Dabigatran anticoagulation | Near-complete radiologic resolution at 4 months | PV controlled | PV hyperviscosity → venous infarction | 4 months follow-up |
| Raza et al., 2021 | China | 78/F | Myeloproliferative Neoplasm | Polycythemia Vera (JAK2+) | Newly diagnosed | RBC 7.0x10^12/L; Hb 220 g/L; Hct high; BM erythroid hyperplasia; JAK2+ | No CNS leukemia; basal ganglia dysfunction | Generalized chorea | Face/trunk/limb jerks; exophthalmos; tongue protrusion; leg twisting | 7 days before admission | Slurred speech; poor appetite | None | None | Not reported | CT: periventricular hypodensities; MRI: bilateral patchy T1/T2/FLAIR hyperintensities | Not done | Hydroxyurea | None | No | Tiapride + diazepam + B12 + homocysteine-lowering + hydroxyurea | Complete resolution | Hematologic improvement | Hyperviscosity + microvascular ischemia | Not stated |
| Teive et al., 2020 | Brazil | 71/M | Myeloproliferative Neoplasm | Chronic Myeloid Leukemia (CML) on Imatinib | Long-term therapy | Imatinib mesylate; normal hematologic workup | No CNS leukemia; ocular surface dysfunction | Blepharospasm | Periorbital edema → excessive blinking → forceful eyelid closure | Gradual during imatinib therapy | Photophobia; ocular discomfort; foreign-body sensation | None | Imatinib toxicity | CSF normal | CT/MRI normal | Not done | Imatinib continued | None | No HSCT | Botulinum toxin (70U) | Significant symptomatic improvement | CML stable | Ocular irritation from imatinib | 2-year follow-up |
| Olivier et al., 2020 | Belgium | 76/M | Myeloid malignancy (MDS → AML) | AML transformed from MDS | On azacitidine; Crohn’s on azathioprine | Hydrea, anagrelide, azathioprine; azacitidine | No CNS leukemia; JC-virus PML | Cerebellar ataxia | Confusion, left arm ataxia, gait ataxia | Insidious (5 months) | Cognitive decline | None | Azathioprine + azacitidine immunosuppression | CSF: protein 68 mg/dL; no pleocytosis; JCV+ | CT: left cerebellar hypodensity | MRI: FLAIR hyperintensity pons/peduncle/cerebellum; DWI shine-through | Not done | Stopped azacitidine + azathioprine | None | No | Progressive worsening | Death (palliative care) | Months | JC-virus reactivation (immunosuppression) |
| Obeidat et al., 2019 | Qatar | 24/M | Myeloproliferative Neoplasm | CML – Chronic Phase | 3–4 years on Imatinib | BCR-ABL positive; complete hematologic/cytogenetic response | No CNS leukemia; normal exam except tremor | Fine intention tremor | Bilateral fine tremor; mild and non-progressive | Late onset after 3 years | No cognitive or sensory deficits | None | Imatinib toxicity | Normal metabolic tests | No imaging performed | Not done | Continued Imatinib (400 mg qod) | None | No | Observation only | Neurologically stable | CML stable in molecular remission | TKI neurotoxicity (cerebellar–thalamic pathway) | ~3 years follow-up |
| Betté & Moore, 2020 | USA | 86/M | Myeloproliferative Disorder | Early JAK2V617F-positive MPN (forme fruste PV) | Newly diagnosed | JAK2V617F+; leukocytosis 11.8→16.4; chronic anemia; low EPO (4) | No CNS leukemia; normal MRI | Generalized chorea | Oro-buccal-lingual chorea; facial grimacing; tongue chewing; shoulder shrugging; leg chorea | 1 month | Left hemiparesis (old cervical surgery); weight loss | None | None | Not done | MRI: normal except IAC enhancement | No PET-CT | Hydroxyurea + aspirin | None | No | Tetrabenazine + hydroxyurea | Significant improvement | Hematologic improvement | JAK2-mediated striatal inflammation | 6 months follow-up |
| Wang et al., 2019 | Canada | 68/M | Myeloid malignancy (MDS → HSCT) | Myelodysplastic syndrome (post–allo-HSCT day 742) | Complete remission | HLA 9/10 mismatched donor; on methotrexate; full donor engraftment | No CNS leukemia; biopsy-proven CNS-GVHD | Myoclonus | Right leg > arm myoclonus; progressed to akinesia/mutism | 1 week | Right pyramidal weakness; encephalopathy | None | None | CSF: protein 0.53 g/L; normal glucose; no cells; PCR– | MRI: multiple scattered cortical/subcortical T2/FLAIR lesions; no enhancement | No PET | HSCT prior | None | Yes (allo-HSCT) | High-dose methylprednisolone → prednisone + azathioprine | Relapse, death from infections | Hematologic remission | T-cell immune-mediated CNS-GVHD | 195 days follow-up |
| Safin et al., 2019 | Russia | 40/M | Myeloproliferative Neoplasm | Polycythemia Vera (JAK2+) | Long-standing PV (10 years) | Erythrocytosis: RBC 6.9x10^12/L; Hb 185 g/L; Hct 57%; Plt 717x10^9/L | No CNS leukemia; CANVAS syndrome | Cerebellar ataxia + sensory neuropathy + vestibular areflexia | Gait ataxia, oscillopsia, blurred vision, impaired depth judgment | Since 2007 (progressive) | Distal hypoesthesia, distal paraparesis, autonomic dysfunction | None | None | CSF not reported | MRI: progressive cerebellar atrophy (vermis + hemispheres) | No PET | Hydroxycarbamide + Aspirin + venesection + pentoxifylline | None | No | Supportive therapy + vestibular rehabilitation | Chronic persistent ataxia | Hematologic improvement | PV exacerbation + CANVAS dual pathology | Not stated |
| Rhee et al., 2019 | USA | 69/F | Myeloid malignancy (APL) | Acute Promyelocytic Leukemia | Induction therapy | PML-RARA t(15;17); CD13+, CD33+, CD64+, CD117+, HLA-DR–; WBC 1.3×10^9/L | No CNS leukemia; EEG toxic-metabolic pattern | Myoclonic jerks | Bilateral truncal + limb myoclonus every 1–20 min; cognitive fluctuation | Day 6 of ATRA/ATO | Right-sided hyperreflexia; encephalopathy | None | ATO toxicity | LP deferred; Cr rise 1.0→1.67; Ca 7.7 mg/dL | MRI normal | N/A | ATRA + ATO initially; both held on day 6 | ATRA + idarubicin | No | Levetiracetam; steroids | Full recovery | Remission by day 35 | ATO-induced encephalopathy | Not reported |
| Chan et al., 2019 | USA | 38/F | Myeloproliferative Neoplasm | CML (Chronic Myeloid Leukemia) | On nilotinib (after imatinib failure) | BCR‑ABL fusion; nilotinib 300 mg BID → 150 mg BID → 150 mg QD | No CNS leukemia; MRI/EEG/CSF normal | Dystonia | Left upper‑extremity dystonia; limb elevation/adduction/flexion; fist posturing; head tilt | Weeks after nilotinib start | Dysarthria; cognitive slowing; impaired fluency | Paraneoplastic panel negative | Nilotinib toxicity | CSF normal; no pleocytosis/protein rise | MRI/EEG normal | FDG‑PET normal | Nilotinib dose adjustments | None | No | Nilotinib dose reduction → cessation | Neurological recovery significant | Hematologic response maintained | Off‑target dopamine pathway modulation | Not stated |
| Bhattacharjee, 2019 | UK | 80/F | Myeloproliferative Neoplasm | Polycythemia Rubra Vera (JAK2 V617F+) | Newly diagnosed | Hct 57%; Hb 175 g/L; WBC 14x10^9/L; Plt 6.8x10^9/L; EPO normal; JAK2+ | No CNS leukemia; imaging normal | Generalized chorea | Orofacial/lingual chorea; upper‑limb movements; motor impersistence | 6 months | Headache; unsteadiness; no cognitive decline | Paraneoplastic panel negative | None | Normal metabolic/infectious labs | MRI T2/ADC/DWI normal | CT CAP normal | Phlebotomy + aspirin | None | No | Supportive only | Persistent chorea | Hematocrit improved (<45%) | Hyperviscosity vs irreversible lacunar injury | Not stated |
| Varghese et al., 2018 | India | 44/M | Myeloid malignancy (AML) | Acute Myeloid Leukemia | Consolidation (HIDAC) | HIDAC; prior 3+7 induction; renal dysfunction; gastritis | No CNS leukemia; HIDAC cerebellar syndrome | Cerebellar ataxia + nystagmus | Gait ataxia; left-sided nystagmus; persistent beyond 48–72 h | Day 5 of HIDAC | Vomiting; abdominal pain; hemorrhagic gastritis | None | Cytarabine toxicity | CSF normal | No MRI performed | Not done | HIDAC (stopped on day 5) | None | No | Methylprednisolone 125 mg x 3 days | Resolved | AML under treatment | Immune-mediated HIDAC cerebellar injury | Not stated |
| García-Cabo et al., 2018 | Spain | 96/M | Myeloproliferative Neoplasm | Polycythemia Vera (unbalanced) | Decompensated disease | Rising Hb/Hct/MCV; hydroxyurea recently stopped | No CNS lesion; CT normal; MRA normal; MRI not possible | Hemichorea | Left hemichorea; orofacial + brachial predominance | Acute onset 4 days prior | Frontal lobe syndrome; disinhibition; imitation behavior | None | None | Normal labs except elevated Hb/Hct/MCV | CT normal | Neurosonology normal | Restarted hydroxyurea | None | No | Haloperidol/clonazepam ineffective | Significant improvement | PV improved | Hyperviscosity affecting striatal/frontal perfusion | Not stated |
| Barow et al., 2018 | Germany | 73/M | Myeloproliferative Neoplasm | Polycythemia Vera (JAK2 V617F+) | Initially normal counts; PV diagnosed later | Hct 50.9%; WBC 13.6x10^9/L; Plt 915x10^9/L; BM: hypercellular panmyelosis; JAK2+ | No CNS leukemia; MRI parietal infarct; BG normal | Hemichorea → generalized chorea | Left hemichorea → orolingual chorea; tongue hypertrophy; dysarthria | Acute onset; 3-year evolution | Cognitive slowing; inattentiveness | None | None | Initial labs normal; later ↑WBC/Plt/Hct | MRI: right parietal infarct; BG normal | No PET | Phlebotomy + hydroxyurea + aspirin | None | No | Tetrabenazine (later stopped) | Improved chorea + tongue hypertrophy | Hematologic normalization | Hyperviscosity + microvascular ischemia | 18 months |
| Kmezic et al., 2017 | Sweden | 81/M | Myeloproliferative Neoplasm | Polycythemia Vera (JAK2+) | Long-standing PCV | Hydroxyurea long-term; ASA; phlebotomy; lymphopenia; FASCIA absent T-cell reactivity | No CNS leukemia; MRI consistent with PML | Ataxia + hemiparesis + neglect | Hemiparesis → limb ataxia → dysdiadochokinesia → bradykinesia → dysarthria → neglect | Subacute (days) | Left hemianopia; hemianesthesia; dysarthria; unresponsiveness | None | Hydroxyurea immunomodulation | CSF: JCV PCR 7,400 copies/mL; NfL 16,500 ng/L | MRI: widespread WM lesions; cerebellar peduncle/dentate involvement | No PET | Hydroxyurea + ASA + phlebotomy | None | No HSCT | Supportive care | Death (pneumonia) | No leukemic transformation | PML from JCV reactivation | 2 months |
| Enriquez-Marulanda et al., 2017 | Colombia | 45/F | Myeloid malignancy (AML → HSCT) | Acute Myelogenous Leukemia | Post-Haploidentical HSCT (Day 253) | TBI 13.2 Gy; cyclophosphamide; fludarabine; 5/10 HLA match; prior ocular/hepatic GVHD | No CNS leukemia; biopsy-proven CNS-cGVHD | Hemichorea–Hemiballismus | Right hemichorea with ballistic proximal movements | Acute onset | No cognitive decline initially; later worsening | None | Immunosuppression withdrawal preceding relapse | CSF normal; infectious work-up negative | MRI: occipital lesion + left GP lesion + later STN involvement | No PET | HSCT; immunosuppression tapered | None | Yes (allo-HSCT) | Haloperidol; clonazepam; steroids; amantadine; risperidone | Improved with residual mild chorea | No AML recurrence | T‑cell mediated CNS inflammation (cGVHD) | 7 months |
| Degnan et al., 2016 | UK | 84/M | Myeloproliferative Neoplasm | Polycythemia Vera (JAK2 V617F+) | Newly diagnosed | Hb 199 g/L; WBC 21.2x10^9/L; Hct 0.586; Plt 352x10^9/L; EPO low; JAK2+ | No CNS leukemia; CT normal | Generalized choreoathetosis | Orofacial/lingual/axial/limb chorea; dysarthria; coughing/grunting | 2 months | Dysphagia; bedbound; severe functional decline | Paraneoplastic panel negative | None | CSF normal; viral studies negative | CT: deep white‑matter SVD changes | CT CAP normal | Serial venesection x6 units in 2 weeks | None | No | Diazepam; procyclidine; tetrabenazine (minimal effect) | Mild residual dysarthria/lip‑smacking | Hematologic normalization | Hyperviscosity‑driven BG dysfunction | 18 months |
| Ssentongo et al., 2015 | USA | 48/M | Myeloid malignancy (Ph+ AML) | Acute Myeloid Leukemia (Philadelphia+) | Chronic GVHD, 4 years post-HSCT | 7+3 + imatinib → fludarabine/cyclophosphamide → allo‑HSCT (MUD) | No CNS leukemia; MRI WM lesions; EEG polyspikes | Myoclonic epilepsy | Generalized limb/neck myoclonus; photic‑triggered; falls | Chronic onset | Brief altered awareness; falls | None | None | CSF normal; infectious PCR negative | MRI: periventricular T2/FLAIR hyperintensity | No PET | Tacrolimus + mycophenolate + topical dexamethasone | None | Yes (allo-HSCT) | Multiple AEDs + VNS | Persistent refractory myoclonus | AML in molecular remission | CNS GVHD; WM injury | Not stated |
| Venkatesan et al., 2014 | India | 55/F | Myeloproliferative Neoplasm | Essential Thrombocythemia (JAK2 V617F+) | Newly diagnosed | Plt 1,092×10^3/µL; WBC 14.2×10^3/µL; Hb 15.1; Hct 46.3%; JAK2+; BCR‑ABL− | No CNS leukemia; MRI normal | Generalized chorea | Fleeting limb/face/tongue chorea; dysarthria; social embarrassment | Acute onset | No headache or psychiatric symptoms | ANA/dsDNA negative | None | CSF not done; metabolic tests normal | MRI normal | USG abdomen normal | Hydroxyurea + haloperidol | None | No | Haloperidol partial response | Full recovery | Platelets down to 562x10^3/µL | Hyperviscosity‑mediated neostriatal dysfunction | Not stated |
| Miyagawa et al., 2014 | Japan | 70/F | Myeloproliferative Neoplasm | Polycythemia Vera (JAK2 V617F+) | Long-standing (10 years) | Hct 57%; RBC 7.43M; Hb 17.6; WBC 15.7K; Plt 229K; low EPO; JAK2+ | No CNS leukemia; MRI normal; SPECT hypoperfusion | Generalized chorea | Orofacial–lingual–truncal–limb chorea (L>R); slowly progressive | Chronic | Mild splenomegaly; no family history | None reported | None | CSF not done; routine labs normal | MRI normal | SPECT: diffuse cortical hypoperfusion; frontal improvement post‑treatment | Serial phlebotomies (Hct <45%) | None | No | No specific anti-chorea drugs | No recurrence | PV stable | Hyperviscosity‑related cortical dysfunction | 2 years |
| Liu et al., 2014 | China | 70/F | Myeloproliferative Neoplasm | Polycythemia Vera (JAK2 V617F+) | Newly diagnosed | Hb 201 g/L; Hct 0.658; MCV 89.4 fL; high uric acid; bilirubin ↑; BM erythroid hyperplasia; low EPO; JAK2+ | No CNS leukemia; MRI mild ischemic WM lesions | Generalized chorea | Orofacial–lingual–limb chorea; grimacing; tongue writhing; dysarthria; dysphagia | Acute onset → relapse at 1 month | Euphoria; hypotonia; ↓ reflexes | Not reported | None | CSF not done | MRI: mild chronic ischemic WM lesions | CT chest: mild pulmonary artery dilatation | Hydroxyurea 1500 mg/day + clopidogrel | None | No | Haloperidol (initial response) | Full recovery | Normalization of Hct/Hb | Hyperviscosity + dopaminergic dysregulation | Not stated |
| Kim et al., 2014 | South Korea | 77/F | Myeloproliferative Neoplasm | Polycythemia Vera (JAK2 V617F+) | Newly diagnosed | RBC 7.98x10^12/L; Hb 221 g/L; Hct 0.661; Plt 451K; WBC 12.4K; JAK2+; low EPO | No CNS leukemia; MRI cortical vessel hyperintensity | Generalized chorea | Orofacial–lingual dyskinesia + generalized limb chorea; insomnia | 1 month | Weight loss; insomnia; hypertension | None reported | None | CSF normal; infectious tests negative | MRI: cortical vessel high-signal; no BG abnormality | None | Phlebotomy + hydroxyurea 1 g/day + aspirin | None | No | Chorea resolved after hematocrit control | Full recovery | Hct normalized | Hyperviscosity + dopamine dysregulation | 6 months |
| Adam et al., 2014 | Australia | 32/F | Myeloproliferative Neoplasm | Essential Thrombocythemia (JAK2 V617F+) | Long‑standing untreated (10 yrs) | Plt 975x10^9/L; APTT 37.9s; PT/INR normal; VWF not measured | No CNS leukemia; MRI cerebellar+SAH hemorrhage | None (hemorrhagic presentation) | Gait ataxia; past‑pointing; drowsiness; sensory leg changes | Acute | Headache 6 weeks; sensory symptoms | Not measured | None | CSF not reported | CT/MRI: multifocal cerebellar + SAH bleeds; SWI blooming | No PET | Hydroxyurea → aspirin | None | No | Supportive neurological care | Resolved | Plt normalized to 432x10^9/L | AVWS‑mediated hemorrhage | 4 months |
| Lew et al., 2013 | USA | 87/F | Myeloproliferative Neoplasm | JAK2V617F-positive MPN (unclassifiable) | No overt PV/ET | Hb 15.6; Hct 44.2%; WBC 8.6K; Plt 281K; EPO 12.7; JAK2 allele burden 35% | No CNS leukemia; MRI/MRA normal | Left hemichorea | Left facial/arm/leg chorea; motor impersistence; milkmaid’s grip | Subacute (3 weeks) | Dizziness; dysarthria; transient facial asymmetry | Not reported | None | CSF not reported | MRI/MRA normal | None | Phlebotomy + hydroxyurea + aspirin | None | No | Resolved with hematocrit control | Full recovery | Normal CBC (no PV) | JAK2‑endothelial dysfunction | Not stated |
| Bhargava et al., 2013 | India | 58/F | Myeloproliferative Neoplasm | Polycythemia Vera (JAK2 V617F+) | Newly diagnosed | Hb 175 g/L; WBC 13.48x10^9/L; Plt 305x10^9/L; EPO 4.14 (low); BM panmyelosis; JAK2+ | No CNS leukemia; MRI BG T2/FLAIR hyperintensity | Hemichorea | Right‑sided chorea; quasi‑purposeful; absent in sleep | Acute | No psychiatric/systemic symptoms; mild hepatosplenomegaly | Not reported | None | CSF not done | MRI: bilateral BG hyperintensity | No PET | Phlebotomy → hydroxyurea + aspirin | None | No | Resolved after phlebotomy | Asymptomatic | Hct maintained 42–45% | Hyperviscosity‑mediated | 1.5 years |
| Severs et al., 2012 | Netherlands | 73/F | Myeloproliferative Neoplasm | Polycythemia Vera (JAK2+) | Previously undiagnosed | Hb 18.7 g/dL; Hct 0.57; MCV 88 fL; rising 6 months; JAK2+ | No CNS leukemia; EEG normal; CSF normal | Chorea + frontal lobe syndrome | Oral automatisms; lip/tongue movements; UL chorea; restlessness | Subacute → acute flare | Disinhibition; imitation/utilization behavior; distractibility | Not reported | None | CSF normal | MRI: mild ischemic WM lesions | No PET | Phlebotomy twice weekly | Aspirin | No | Marked improvement after phlebotomy | Remission at 6 months | Hb/Hct normalized | Hyperviscosity mechanism | 6 months |
| Huang et al., 2011 | Taiwan | 70/F | Myeloproliferative Neoplasm | Polycythemia Vera (JAK2 V617F+) | Untreated at presentation | RBC 5.91M; Hb 16.8; Hct 51.7%; Plt 769K; EPO normal; JAK2+ | No CNS leukemia; MRI normal | Generalized chorea | Orofacial‑lingual + limb chorea; hypotonia; ↓ reflexes; erythromelalgia | Subacute | Erythromelalgia; hypotonia | Not reported | None | CSF normal | MRI normal | PET/SPECT abnormalities reversible | Phlebotomy monthly x5 | None | No | Improved after Hct control | Full recovery | Hct normalized | Hyperviscosity + reversible DAT deficit | 10 months |
| Ben Ghorbel et al., 2011 | Tunisia | 78/M | Myeloproliferative Neoplasm | Polycythemia Vera (JAK2V617F+) | Newly diagnosed | Hb 20 g/dL; Hct 62.3%; RBC mass x2; WBC/Plt normal; EPO normal; BM hypercellular; JAK2+ | No CNS leukemia; CT normal | Generalized chorea | Large‑amplitude limb + facial chorea; suppressible; absent in sleep | 1 month | Headache; splenomegaly on CT | Not reported | None | CSF not done | CT brain normal | CT abdomen: splenomegaly | Phlebotomy twice weekly + hydroxyurea 200 mg/day | None | No | Improved after Hct reduction | Asymptomatic | Hb 15.2; Hct 43.9 | Hyperviscosity mechanism | 15 months |
| Kumar et al., 2009 | Canada | 82/F | Myeloproliferative Neoplasm | Polycythemia Vera (JAK2 V617F+) | Newly diagnosed | RBC 6.92M; Hb 181 g/L; Hct 56%; Plt 492K; WBC 9.6K; EPO low; MRI: atrophy only | No CNS leukemia; MRI normal | Generalized chorea | Oro‑facio‑lingual → trunk/limb chorea; absent in sleep; continuous | Acute | No behavioral change; mild areflexia; plethora | Not reported | None | CSF not done | MRI: cortical atrophy only | No PET/SPECT | Hydroxyurea 1000 mg/day | None | No | Improved at 2 weeks | No recurrence | Hct normalized | Hyperviscosity + microvascular dopamine dysregulation | 6 months |
| Pratesi et al., 2008 | Italy | 65/M | Myeloproliferative Neoplasm | Polycythemia Vera (PV) | Long‑standing PV | JAK2 not reported (pre‑routine testing) | No CNS leukemia; MRI pallidal hyperintensity | Parkinsonism | Bradykinesia, rigidity, hypomimia, gait difficulty | Gradual | Mild cognitive slowing; gait freezing | Negative | None | Not reported | MRI: bilateral GP T1 hyperintensity | Not done | Phlebotomy + hydroxyurea | None | No | Levodopa trial | Persistent parkinsonism | Hct improved with therapy | Manganism (pallidal Mn deposition) | Several months |
| Lee et al., 2008 | South Korea | 58/F | Myeloproliferative Neoplasm | Polycythemia Vera (PV) | Newly diagnosed | JAK2 V617F homozygous; BM cellularity 60%; MF‑1 | No CNS leukemia; MRI/EEG normal | Hemichorea | Right UL/LL + facial chorea; action‑induced; absent in sleep | Acute (5 days) | Dizziness; mild headache; gait unsteadiness | Negative | None | Not done | MRI normal | SPECT: ↑ perfusion left basal ganglia | Phlebotomy 400 mL x multiple | None | No | Haloperidol 1.5–3 mg/day | Near complete resolution | Hct/Hb controlled | Hyperviscosity + striatal perfusion changes | 2 months |
| Kim et al., 2008 | South Korea | 65/M | Myeloproliferative Neoplasm | Polycythemia Vera (PV) | Newly diagnosed | Hb 18.8; Hct 56%; Plt 378K; WBC 9.7K; EPO 1.0; BM hypercellular | No CNS leukemia; MRI normal | Generalized chorea‑ballism | Ballistic, large‑amplitude, irregular movements; absent in sleep | Acute (2 days) | Facial erythrósis; splenomegaly | Negative | None | CSF not done | MRI normal | No PET | Phlebotomy x3 + Hydroxyurea 500 mg/day | None | No | Supportive care only | Complete resolution | Hb/Hct normalized | Non‑hypoperfusion neurotransmitter mechanism | 6 months |
| Dubow et al., 2008 | USA | 55/F | Myeloid malignancy | Acute Myelogenous Leukemia (post-autologous HSCT) | Post‑HSCT with chronic GVHD, CMV viremia | Molecular markers not reported | No CNS leukemia; MRI/MRA normal | Acute generalized dystonia | Facial/jaw/arm dystonia; severe rigidity; aphonia; painful | Acute (30 minutes after foscarnet) | Aphonia; pain; normal mental status | Not tested | Foscarnet neurotoxicity | CSF not done | MRI/MRA normal | None | AML therapy + immunosuppression; IV foscarnet for CMV | None | Yes (autologous HSCT) | IV diphenhydramine | Complete recovery in 24 hours | Stable post-transplant status | Drug-induced dystonia (foscarnet) | Not stated |
| Midi et al., 2006 | Turkey | 63/F | Myeloproliferative Neoplasm | Polycythemia Vera (PV) | Newly diagnosed | Hb 18.1; Hct 59.2%; WBC 12.8K; Plt 684K; EPO suppressed; JAK2 not tested | No CNS leukemia; MRI normal | Left hemichorea | Irregular jerks of face/arm/leg; worse with action; absent in sleep | Subacute | Mild dysarthria; no cognitive deficits | Not tested | None | Not done | MRI normal | Not done | Phlebotomy + Hydroxyurea 1 g/day + Aspirin | None | No | Haloperidol 1 mg/day | Complete recovery | Hematocrit normalized | Hyperviscosity mechanism | 3 months |
| Golla & Thier, 2005 | Germany | 56/F | Myeloid malignancy | Acute Myeloid Leukemia (post-allogeneic PBSCT) | Post-transplant day 89 | Molecular markers not reported | CSF leukemic meningitis with blasts; MRI normal | Ocular flutter + cerebellar ataxia | Horizontal flutter, truncal/limb tremor, oscillopsia, gait inability | Subacute | Vertigo, oscillopsia, dizziness | Not tested | Cyclosporin neurotoxicity | CSF: 54–94 cells; blasts 5%; leukemic meningitis | MRI brain/spine normal | None | Allo-PBSCT + cyclosporin + intrathecal chemo | None | Yes (allo-PBSCT) | Stopped cyclosporin | Near-complete resolution | AML relapse (meningitis treated) | Drug-induced brainstem dysfunction | 95 days |
| Bishton et al., 2005 | United Kingdom | 30/F | Myeloid malignancy | Acute Myeloid Leukemia (AML) | Post‑allo HSCT (day +60) | Molecular markers not reported; 83% donor chimerism | No CNS leukemia; MRI/CT/CSF normal | Opsoclonus–Myoclonus Syndrome | Opsoclonus, coarse tremor, ataxia, dysdiadochokinesis, dysmetria | Subacute | Ataxic gait; speech deterioration | Anti‑Hu negative | None | CSF normal | MRI/CT normal | None | Allo‑HSCT; conditioning with TBI/CY/CAMPATH; foscarnet trial | None | Yes (allo‑HSCT) | Supportive only | Partial recovery | AML relapse → death | Immune‑mediated OMS preceding relapse | 2–3 months |
| Tan et al., 2003 | Singapore | 49/F | Myeloid malignancy | Chronic Myeloid Leukemia (CML) | Chronic phase on IFN‑α2a | Variant Ph translocation (9;13;22) | No CNS lesion; MRI normal | Myorhythmia | Slow rhythmic 2–3 Hz facial tremor; synchronous contractions | Gradual | None | Not tested | Interferon‑α2a neurotoxicity | Not done | MRI normal | None | Interferon‑α2a up to 6 MU/day; later protein kinase inhibitor | Protein kinase inhibitor | No | Stopped interferon; acupuncture | Near-complete resolution at 1 month | Hematologic response maintained | Drug-induced myorhythmia mechanism | Several months |
| Sarasombath et al., 2002 | USA (Hawaii) | 79/M | Myeloid malignancy | Chronic Myelogenous Leukemia (CML) | Chronic phase on IFN-α2b | Philadelphia chromosome (Ph+) | No CNS leukemia; CT/MRI show only old infarcts | Interferon-induced parkinsonism | Rest/action tremor, bradykinesia, cogwheel rigidity, gait difficulty | Subacute | Confusion, postural instability, falls | Not tested | Interferon-α2b neurotoxicity | Not done | CT/MRI: mild atrophy + old BG/pontine infarcts | None | IFN-α2b therapy for CML | None | No | Stop IFN + levodopa | Returned to baseline | Stable CML | Dopaminergic suppression & unmasking effect | 1 month |
| Friedman & Shetty, 2001 | USA | 27/F | Myeloid malignancy | Acute Myeloid Leukemia (AML) | Post‑induction; consolidation with high‑dose Ara‑C | Not reported | No CNS leukemia; imaging normal | Cerebellar ataxia | Truncal ataxia, gait ataxia, dysmetria, dysarthria, nystagmus | Acute/Subacute | Dysarthria; severe functional impairment | Not applicable | Ara‑C cerebellar neurotoxicity | Normal | No abnormalities reported | Not done | High‑dose cytarabine | None | No | Supportive care + rehabilitation | Permanent cerebellar syndrome | AML outcome not detailed | Ara‑C‑induced Purkinje cell toxicity | Long‑term follow‑up |
| Nazabal 2000 | Spain | 74/F | Myeloproliferative neoplasm | Polycythaemia vera | Deterioration due to iron therapy | None reported | No CNS leukemia; movement disorder due to PV | Chorea | Generalized violent chorea with orofaciolingual movements | Acute onset (4 days) | Dysarthria; hypotonia; diminished reflexes | Clinical correlation; rapid response to venesection | None | No drug toxicity | Normal CSF | Normal MRI | Not done | Venesection | None | None | Marked improvement | Normal hematology post-treatment | Hyperviscosity; iron-induced worsening | Follow-up: several weeks |
| Apsner 1997 | Austria | 17/F | Hematologic malignancy | PML‑RARα positive acute myeloid leukemia | Second remission pre‑BMT | PML‑RARα positive | No CNS leukemia; ocular flutter due to CsA toxicity | Ocular flutter | Rapid symmetric horizontal conjugate saccadic oscillations | Day 51 post‑BMT | Vertigo, vomiting, drop attacks, blurred vision | Clinical diagnosis + resolution after CsA withdrawal | None | Cyclosporin A toxicity | Normal CSF | Normal MRI | Not done | CsA stopped; steroids given | None | None | Complete clinical resolution in 3 weeks | No recurrence; mild persistent EOG abnormality | Drug‑induced toxicity (CsA) | 10‑month follow‑up documented |
| Mott 1995 (Case series, 3 children) | USA | 12/M | Leukaemia | AML (Second CR) | Post-BMT | Not reported | Leukoencephalopathy + basal ganglia involvement | Parkinsonism | Rest tremor, rigidity, bradykinesia, shuffling gait, hyperreflexia, dysmetria | 7 weeks post-BMT | Oromotor dysfunction, cognitive decline | Not tested | High-dose amphotericin B | Protein 76 mg/dL; normal cells | MRI: basal ganglia signal + frontal/temporal WM lesions + cerebellar atrophy | Not done | HD Ara-C, cyclophosphamide, TBI, BMT, MTX | None | Allogenic BMT (HLA-identical) | Levodopa/carbidopa, selegiline | Near baseline | Engraftment with GVHD | AmB-induced BG + WM toxicity | Months |
|  |  | 8/M | Leukaemia | AML (Second CR) | Post-BMT | Not reported | Drug-induced leukoencephalopathy | Parkinsonism | Mask facies, bradykinesia, rigidity, dysmetria, mild ataxia, resting tremor | 2 weeks after AmB | Dysarthria, encephalopathy | Not tested | Amphotericin B + ABLC | Mildly ↑ protein; normal cells | MRI: ventriculomegaly + frontal WM changes | Not done | HD Ara-C, cyclophosphamide, TBI, BMT, steroids, ATG | None | Allogenic BMT | Stopping amphotericin | Residual mild cognitive impairment | Stable transplant | AmB/ABLC WM toxicity | 4 months |
|  |  | 8/M | Leukaemia | AML (Second CR) | Post-BMT | Not reported | Drug-induced leukoencephalopathy | Parkinsonism | Mask facies, bradykinesia, rigidity, dysmetria, mild ataxia, resting tremor | 2 weeks after AmB | Dysarthria, encephalopathy | Not tested | Amphotericin B + ABLC | Mildly ↑ protein; normal cells | MRI: ventriculomegaly + frontal WM changes | Not done | HD Ara-C, cyclophosphamide, TBI, BMT, steroids, ATG | None | Allogenic BMT | Stopping amphotericin | Residual mild cognitive impairment | Stable transplant | AmB/ABLC WM toxicity | 4 months |
| Leferink 1995 | Germany | 37/F | Hematologic malignancy | Acute myeloid leukemia (FAB M6) | During consolidation cycle; cumulative Ara-C 22.5 g/m2 | Not reported | No CNS leukemia; drug-induced cerebellar toxicity | Cerebellar syndrome | Severe dysarthria, scanning speech, marked limb ataxia, unable to stand/walk | During 3rd chemo protocol (March 1994) | No sensory/motor deficits; reflexes normal; no nystagmus | Clinical onset post-AraC + reversibility after withdrawal | None | Cytarabine neurotoxicity | CSF: 1 cell/μL, protein 11 mg/dL, normal cytology | MRI normal | Not done | Induction + HAM + consolidation | Daunorubicin, Thioguanine, Novantron | None | Full resolution in 3 weeks | Full neurological recovery | Purkinje cell dysfunction (literature-supported) | Follow-up 3 weeks–1 month |
| Krstenansky 1994 | USA | 35/M | Hematologic malignancy | Acute myelogenous leukemia (second remission) | During chemotherapy before autologous BMT | Not reported | No CNS leukemia; drug-induced movement disorder | Drug-induced extrapyramidal reaction | Involuntary flexing of arms, mouth distortion, lip twitching, severe jerking | After 10 doses of ondansetron over 3 days | Numbness, jerking of fingers; anxiety during reaction | Clinical temporal association; resolution after stopping drug | None | Ondansetron toxicity | Not performed | Not reported | Not done | Busulfan, phenytoin, amphotericin spray, pentoxifylline, allopurinol, lorazepam, TMP-SMX | None | None | Full resolution after lorazepam | Good neurological recovery | Serotonergic–dopaminergic imbalance (proposed) | Follow-up: remainder of chemotherapy without recurrence |
| Balmaceda 1994 | USA | 32/F | Hematologic malignancy | Acute promyelocytic leukemia | Post-BMT; cumulative AmB 1.64 g | Not reported | No CNS leukemia; drug-induced encephalopathy | Encephalopathy + parkinsonism | Bradykinesia, rigidity, resting tremor, dystonia, mutism, imbalance | Progression over 8 weeks after AmB | Dysphagia, frontal release signs, incontinence | Clinical association + improvement after stopping AmB | None | Amphotericin B toxicity | CSF normal | MRI normal | NA | Induction + TBI + BMT; antifungals | None | None | Gradual improvement after stopping AmB | Almost complete recovery except mild memory loss | Early reversible toxic leukoencephalopathy | Follow-up to June 1993 |
| Cohen 1989 | Israel | 65/F | Myeloproliferative neoplasm | Polycythemia vera | At presentation | None reported | No CNS leukemia; hyperviscosity-related chorea | Chorea (generalized) | Faciobuccal dyskinesia; widespread arrhythmic rapid jerky movements; hypotonia | At initial presentation | Dysarthria, dizziness, vertigo; splenomegaly; retinal vein dilation | Clinical response to phlebotomy + recurrent symptoms with high Hb | None | No drug toxicity | Not reported | CT scan normal (sulci, ventricles, basal ganglia) | Not done | Phlebotomy (500 mL biweekly x 4) | None | None | Complete resolution after phlebotomy; recurrence reversed on repeat phlebotomy | Good; asymptomatic at 2-year follow-up | Hyperviscosity → venous stasis → reduced cerebral blood flow | Follow-up: 2 years |
| Luque 1987 | USA | 64/F | Hematologic malignancy | Acute myelomonocytic leukemia | Relapse; treated with 72 g HD Ara-C | Not reported | No CNS leukemia; drug toxicity | Parkinsonism | Pill-rolling tremor, mask-like facies, cogwheel rigidity, jaw tremor, bradykinesia | Onset 3 weeks after HD cytarabine | Absent ankle jerks; hair loss; postural hypotension later | Temporal relation; resolution after drug withdrawal | None | Cytarabine toxicity | CSF normal | CT and MRI normal | Not done | Induction + relapse chemotherapy; antibiotics; antifungals | Tobramycin, mezlocillin, amikacin, amphotericin B, acyclovir | None | Improved then worsened; full resolution at 12 weeks | Returned to full activity; no residual tremor | Dopamine receptor/metabolic disruption suspected | Follow-up till complete recovery |
| Cersosimo 1987 | USA | 72/M | Hematologic malignancy | Non‑Hodgkin lymphoma receiving cytarabine | During low‑dose cytarabine 30 mg/m² q12h × 7 days | Not reported | No CNS leukemia; drug‑induced neurotoxicity | Acute cerebellar syndrome | Ataxia, dysarthria, nystagmus, imbalance, scanning speech | Day 6–7 of cytarabine therapy | Conjunctivitis, bilateral hearing loss, tinnitus, headache | Temporal relation + improvement after discontinuation | None | Cytarabine toxicity | CSF normal | CT normal; no cerebellar lesion | Not done | Cyclophosphamide, vincristine, prednisone; low‑dose Ara‑C | None | None | Marked improvement within 1 week | Residual mild hearing loss | Cytarabine‑induced cerebellar and auditory pathway dysfunction | Follow‑up: several months |
| Vilter 1986 | USA | 24/M | Myeloproliferative/post‑BMT condition | Chronic myeloid leukemia (post–bone marrow transplant) | 9 months post‑BMT; 7‑day vidarabine course | None reported | No CNS leukemia; drug‑induced encephalopathy | Encephalopathy + severe myoclonus | Whole‑body myoclonus, tremor, hallucinations, delirium, torticollis‑like posturing | Symptoms began 1 day after stopping vidarabine | Emotional lability, diaphoresis, agitation, neck pain, instability | Temporal association + improvement after withdrawal | None | Vidarabine toxicity | CSF: 40 lymphocytes/µL, protein 40 mg/dL, glucose 66 mg/dL | CT normal; carotid/basilar high attenuation (possible arteriosclerosis) | Not done | Bone marrow transplant regimen; antivirals; supportive | None | None | Marked improvement by day 6 | Full recovery by 9 months | Drug–viral interaction + hepatic dysfunction | Follow‑up: 9 months |
| Cold 1986 | Denmark | 56/F | Hematologic malignancy | Acute myelogenous leukemia (FAB M6) | Consolidation with HDCA; cumulative dose 32 g/m2 | None reported | No CNS leukemia; Ara‑C toxicity | Persistent cerebellar dysfunction | Ataxic dysarthria, limb ataxia, unsteady gait, dysdiadochokinesia | Day 6 of HDCA therapy | Cutaneous rashes earlier in therapy; no fever; platelets >90×10⁹/L | Strong temporal relationship + literature correlation | None | Cytarabine toxicity | Not done (no LP ever performed) | Not available (no CT/MRI performed) | Not done | Induction daunorubicin + Ara‑C; HDCA consolidation | Allopurinol | None | Marked improvement; residual ataxia at 10 months | Persistent mild cerebellar dysfunction | Purkinje cell toxicity due to high CSF Ara‑C levels | 10‑month follow‑up |
| Mas 1985 | France | 72/F | Myeloproliferative neoplasm | Polycythaemia vera | At presentation; several months symptoms | None reported | No CNS leukemia | Generalized chorea | Violent limb/oral chorea; dysarthria; grimacing | Gradual onset → worsening | Vertigo; visual blur; erythrosis; venous engorgement | Clinical response to venesection | None | No drug toxicity | CSF normal (literature data) | CT normal | No PET | Venesection + 32P | None | None | Marked improvement | Good outcome; recurrence when Hct ↑ | Hyperviscosity mechanism | 4-year follow-up |
|  |  | 73/F | Myeloproliferative neoplasm | Polycythaemia vera | At presentation; acute worsening | None | No CNS leukemia | Unilateral chorea (right‑sided) | Orofacial and right‑limb chorea; grimacing | Sudden worsening | Epistaxis; dizziness; venous engorgement | Xe133 CBF low bilaterally | None | No toxicity | CSF normal (lit) | CT normal | Xe133: decreased bilateral flow | Repeated venesections | None | None | Rapid improvement | Good, intermittent relapses with high Hct | Hyperviscosity | Follow-up reported |
| Dworkin 1985 | USA | 47/M | Hodgkin’s disease → secondary acute non‑lymphocytic leukemia | Secondary ANLL after HL therapy | High‑dose Ara‑C: 3 g/m² q12h; cumulative 30–36 g/m² | Not reported | No CNS leukemia; Ara‑C neurotoxicity | Severe cerebellar syndrome | Nystagmus, severe truncal ataxia, dysarthria, impaired coordination | Onset after 10 doses (30 g/m2 cumulative) | Hematologic improvement but persistent cerebellar dysfunction | Autopsy: extensive Purkinje cell loss | None | Ara‑C toxicity | CSF not reported | MRI/CT: not described (clinical diagnosis) | No PET | High‑dose Ara‑C cycles; subsequent consolidation | Daunomycin, 6‑thioguanine (later regimen) | None | Persistent, worsening on retreatment | Severe disability until death | Purkinje cell destruction documented | Follow‑up until death |
| Coppack 1985 | UK | NA | Myeloproliferative neoplasm | Transitional myeloproliferative disease (TMPD) | Polycythaemia → myelofibrosis → back to polycythaemia | No molecular markers (1985 era) | No CNS leukemia | Generalized chorea (orofacial emphasis) | Orofacial dyskinesia, limb chorea, hypotonia, stress‑induced worsening | Chorea since 1965; worsened whenever Hb/Hct increased | Attacks of unresponsiveness, memory decline, mouth ulcers from trauma | Improved when hematocrit lowered; worsened with Hb rise | None | No drug toxicity | CSF not performed | CT: cerebral atrophy + small infarcts R cerebellum, L parietal; basal ganglia normal | No PET | Radio‑phosphorus (1971); venesection (1972–73; 1983) | None | None | Improved with venesection; resolved when anaemic | Good overall; fluctuating with hematocrit | Hyperviscosity + prior rheumatic basal‑ganglia damage | Follow‑up over >15 yrs |
| Saper 1982 | USA | 31/F | Acute myelomonocytic leukemia (FAB M4) | Cerebellar leukemic infiltration | Relapse with CNS involvement | Myeloperoxidase deficiency; LAP score 0 | Yes – CSF with 59% myeloblasts; cerebellar mass | Cerebellar syndrome + meningismus | Unsteady gait, left-sided ataxia, dysmetria, hyperreflexia | Symptoms appeared July–Aug 1978 | Left facial numbness; limb weakness; vomiting; dysphasia later | CSF cytology + CT defining mass; resolution with chemo‑RT | None | No drug toxicity | CSF: WBC 605/mm3 (59% myeloblasts), protein 107 mg/dL, glucose 72 mg/dL | CT: enhancing cerebellar mass; resolved post‑therapy | No PET | IV Ara‑C + daunorubicin; whole‑brain RT 2400 rads; intraventricular Ara‑C + MTX | Lomustine, thioguanine, cyclophosphamide | Ommaya reservoir therapy | Neurologic resolution initially; later PML-related decline | Died from sepsis + leukemia relapse | CNS leukemia + subsequent PML | Follow‑up approx. 3 years |
| Morré et al., 1982 | Netherlands | 76/F | Myeloproliferative neoplasm | Polycythemia Vera | Longstanding PV with deterioration | Pre‑JAK2 era; Hb 12.2 mmol/L; Hct 64.5%; RBC 8.44×10^12/L; Plt 488K; BM hyperplasia | CT: small infarct near right claustrum/putamen; EEG abnormal | Generalized chorea | Facial grimacing, limb chorea, tongue protrusion, dysarthria | Gradual | Dysarthria, slight left arm weakness, dizziness | Not tested | None | Not performed | CT: lacunar infarct; dynamic perfusion normal | Not done | Phlebotomies ×7; P‑32 therapy | None | No | Supportive only | Complete resolution | Normalization of hematologic values | Hyperviscosity-mediated basal ganglia dysfunction | 1–2 months |
| Borg-Costanzi et al., 1981 | UK | 59/M | Myeloproliferative neoplasm | Primary Polycythaemia Vera | Newly diagnosed; severe neurological presentation | Pre-JAK2 era: Hb 21.8 g/dL; PCV 0.686; RBC 9.21x10^12/L; WBC 19.1x10^9/L; Plt 183x10^9/L; LAP 214; pan-hypercellular marrow | No CNS lesion on CT; EEG with nonspecific abnormalities | Generalized chorea | Severe choreiform movements of face and limbs; slurred speech; gait difficulty | Subacute | Severe dysarthria; inability to walk without support | Not tested | None | Not performed | CT normal; EEG abnormal | Not done | Venesections x3 initially; later x4; volume replacement | None | No | Tetrabenazine up to 75 mg/day | Marked improvement | Hematologic parameters improved after venesections | Hyperviscosity-related basal ganglia dysfunction | Several weeks |
| Voiculescu et al., 1979 | Romania | 53/F | Myeloproliferative neoplasm | Polycythemia Vera | Longstanding PV (1 year symptoms) | Hct 65%; Hb 19.6 g/dL; RBC 7.2M; WBC 12K; Plt 220K; LAP 96; RBC volume 75 ml/kg | No lesion; normal tone | Generalized chorea | Upper-limb purposeless movements; gait disturbance; tongue protrusion; sucking movements | Subacute | Headaches; itching; memory issues; bizarre behavior | Not tested | None | Not reported | Not performed | Not applicable | Venesections (400 ml ×2, then serial 350–500 ml) | None | No | None | Full recovery | Normalization | Hyperviscosity‑related basal ganglia dysfunction | 2 years |
|  |  | 72/M | Myeloproliferative neoplasm | Polycythemia Vera | Newly diagnosed; 6‑week history | Hct 68%; Hb 19 g/dL; RBC 6.8M; WBC 9K; Plt 260K; LAP 92; RBC volume 59 ml/kg | Xenon‑133: lowest BG flow bilaterally | Generalized chorea | Facial grimacing; limb chorea; sucking/swallowing; jaw jerks | Subacute | Red face/nails; itching; splenomegaly | Not tested | None | Not reported | Perfusion study abnormal | Not applicable | Venesections (400 ml x2; serial extractions) | None | No | None | Full recovery | Normalization | Hyperviscosity‑mediated BG dysfunction | 5 months |
| Edwards et al., 1975 | UK | 26/M (primary case reported) | Myeloproliferative/neurohematologic association | Polycythaemia + Cyanotic Congenital Heart Disease | Longstanding cyanotic CHD with secondary polycythaemia | No molecular markers available (pre‑JAK2 era); Hb markedly elevated; Hct ~70% | No CNS infiltrative disease; no focal lesion on neuro exam | Chorea | Generalized chorea with facial grimacing, limb jerks, dysarthria | Subacute | Dysarthria; exercise‑induced worsening; hypoxia‑related symptoms | Not tested | None | Not performed | No acute lesion reported | Not done | Venesection; oxygen therapy | None | No | Supportive management; oxygen; phlebotomy | Improved significantly | Hematologic improvement after venesection | Hyperviscosity + chronic hypoxia disrupting basal ganglia circuits | Weeks–months follow‑up reported |
| Ashenhurst et al., 1972 | Canada | 68/M | Myeloproliferative neoplasm | Polycythemia Rubra Vera | Long‑standing PV with 1971 deterioration | Pre‑JAK2 labs: Hb 17–21 g/dL; Hct ~65%; RBC 7–8M; Plt ~500K; B12↑; uric acid↑ | No focal CNS lesion reported | Generalized chorea | Violent limb chorea, facial chorea, dysarthria, flailing movements | Acute | Tinnitus, dizziness, hypersomnia, headaches, instability | Not tested | None | Not performed | No CT/MRI (1972 era) | Not applicable | Venesections; 32P therapy (1969–71) | None | No | Supportive only | Complete recovery | Hematologic normalization | Hyperviscosity‑mediated cerebral dysfunction | ≥1 month |
| Friedemann et al., 1965 | Switzerland | 62/F | Myeloproliferative neoplasm | Polycythaemia vera | Long‑standing PV; clinical worsening in 1964 | Pre‑JAK2: Hb 18–18.5 g/dL; RBC 6–6.5M; Hct 62%; low iron; ↑iron turnover; RBC mass 43.6 ml/kg | No structural CNS lesion; EEG diffuse dysrhythmia; CSF normal | Generalized chorea | Violent generalized chorea, grimacing, tongue movements with ulceration, gait inability | Subacute | Dysarthria; instability; tongue ulcerations | Not applicable | None | Normal CSF | Normal pneumoencephalogram; normal ventricles; no basal ganglia lesion | Not available | Phlebotomy; hydration; anticoagulation; 32P (5 mCi IV) | None | No | Librium; supportive therapy | Complete resolution | Hematologic remission (Hb 14 g/dL; Hct 47%) | Hyperviscosity‑induced basal ganglia dysfunction | 4.5 months follow‑up |

**ADC; Apparent Diffusion Coefficient; AEDs; Anti-Epileptic Drugs; ALL; Acute Lymphoblastic Leukemia; allo-HSCT; Allogeneic Hematopoietic Stem Cell Transplantation; AmB; Amphotericin B; AML; Acute Myeloid Leukemia; ANA; Antinuclear Antibody; APML; Acute Promyelocytic Leukemia; Ara-C; Cytarabine; ATO; Arsenic Trioxide; ATRA; All-Trans Retinoic Acid; AVWS; Acquired Von Willebrand Syndrome; BG; Basal Ganglia; BM; Bone Marrow; BMT; Bone Marrow Transplantation; CANVAS; Cerebellar Ataxia, Neuropathy, Vestibular Areflexia Syndrome; CASPR2; Contactin-Associated Protein-like 2; cGVHD; Chronic Graft-Versus-Host Disease; CHD; Cyanotic Congenital Heart Disease; CML; Chronic Myeloid Leukemia; CNS; Central Nervous System; CSF; Cerebrospinal Fluid; CT; Computed Tomography; DWI; Diffusion-Weighted Imaging; EEG; Electroencephalogram; ENMG; Electroneuromyography; EPO; Erythropoietin; ET; Essential Thrombocythemia; FAB; French-American-British classification; FDG-PET; Fluorodeoxyglucose Positron Emission Tomography; FLAIR; Fluid-Attenuated Inversion Recovery; FLT3-TKD; FLT3 Tyrosine Kinase Domain mutation; GP; Globus Pallidus; GVHD; Graft-Versus-Host Disease; Hb; Hemoglobin; Hct; Hematocrit; HDAC; High-Dose Cytarabine; HDAmB; High-Dose Amphotericin B; HiDAC; High-Dose Cytarabine; HIDAC; High-Dose Ara-C; HSCT; Hematopoietic Stem Cell Transplantation; IFN-α; Interferon-alpha; IVIG; Intravenous Immunoglobulin; JAK2; Janus Kinase 2; JCV; JC Virus; LGI1; Leucine-rich Glioma-Inactivated 1; LP; Lumbar Puncture; MDS; Myelodysplastic Syndrome; MF; Myelofibrosis; MMF; Mycophenolate Mofetil; MPN; Myeloproliferative Neoplasm; mPSL; Methylprednisolone; MRI; Magnetic Resonance Imaging; MUD; Matched Unrelated Donor; OMS; Opsoclonus-Myoclonus Syndrome; PCR; Polymerase Chain Reaction; PET-CT; Positron Emission Tomography–Computed Tomography; Ph+; Philadelphia chromosome positive; Plt; Platelets; PML; Progressive Multifocal Leukoencephalopathy; PMF; Primary Myelofibrosis; PV; Polycythemia Vera; RBC; Red Blood Cell; SPECT; Single-Photon Emission Computed Tomography; STN; Subthalamic Nucleus; TKI; Tyrosine Kinase Inhibitor; VEMP; Vestibular Evoked Myogenic Potential; VWF; Von Willebrand Factor; WBC; White Blood Cell; WM; White Matter2.3sFast**

**References**

1. Zhao C, Zhang Q, Zhang M, Zhang J, Bao X. Metronidazole-induced encephalopathy in a patient with acute myeloid leukemia type 2 and a literature review of pediatric case reports. Front Pediatr. 2025;13:1656227. DOI: 10.3389/fped.2025.1656227.
2. Vijayaraghavan A, Nair SS, Lalitha LP, Sukumaran S, Sundaram S. Morvan syndrome with bilateral basal ganglia hyperintensities and exfoliative skin rashes in a patient with chronic hepatitis B. Clin Neurol Neurosurg. 2025;257:109119. DOI: 10.1016/j.clineuro.2025.109119.
3. Sodemann NW, Brask-Thomsen PK, van Kooten Niekerk PB. Hemichorea caused by polycythemia vera. Ugeskr Laeger. 2025;187:V04250261. DOI: 10.61409/V04250261.
4. Sassani M, Liang D, Samra AD. Palatal-Lingual-Facial Myoclonus Due to Acute Cortical Infarction in JAK2V617F Positive Myelodysplastic Syndrome. Mov Disord Clin Pract. 2025. DOI: 10.1002/mdc3.70234.
5. **Saibaba J, Selvaraj J, Viswanathan S, Pillai V. Cerebellar Ataxia With Neuropathy and Bilateral Vestibular Areflexia Syndrome Coexisting With JAK2-Positive Polycythemia Vera and Myelofibrosis. Ochsner J. 2025;25(1):50–53. DOI: 10.31486/toj.24.0088.**
6. Ma L, Huang N, Zhang H, Liu J, Zhang Z. Case report: Dysphonia associated with high-dose cytarabine therapy. Front Pharmacol. 2025;16:1518298. DOI: 10.3389/fphar.2025.1518298.
7. Lee J-S, Jung D-E, Kwag D, Kim J-S, Yoo S-W. Ischemic penumbra-induced hemichorea complicated by polycythemia vera: Promotion of thalamic disinhibition. Neurol Sci. 2025;46(2):1035–1037. DOI: 10.1007/s10072-024-07804-1.
8. Harrison A, Zhang T, Batra G. A case of hemichorea–hemiballismus secondary to a subacute hemorrhage in a patient with essential thrombocythemia: a case report. *Journal of Medical Case Reports*. 2025;19(1):142. doi:10.1186/s13256-025-05010-9
9. Rusche T, Yaldizli Ö, Galbusera R, Mutke M, Halter JP, Lieb J, et al. Anti-GABAA receptor encephalitis 14 months after allogeneic haematopoietic stem-cell transplant for acute myeloid leukaemia. Lancet. 2024;403:469–470. DOI: 10.1016/S0140-6736(24)00197-2.
10. Rocha-Cadman X, Revilla AR, Cadman K, Root JC. Hemichorea–hemiballismus associated with a case of cerebral toxoplasmosis in a hematopoietic stem cell transplant recipient. Palliat Support Care. 2024;22(3):623–625. DOI: 10.1017/S1478951524000105.
11. Butnariu I, Antonescu-Ghelmez D, Moraru A, Anghel DN, Cojocaru FM, Tuţă S, et al. Chorea and Cognitive Impairment in JAK2V617F-Positive Myeloproliferative Disorders: A Case Report and Literature Review. Medicina (Lithuania). 2024;60(1):18. DOI: 10.3390/medicina60010018.
12. Takahashi K, Katayama T, Ichikawa T, Matsuoka S, Kakinoki Y, Yoneda M, et al. Possible chronic graft-versus-host disease in the central nervous system manifesting as cerebellar ataxia after allogeneic hematopoietic stem cell transplantation for acute myeloid leukemia. Intern Med. 2023;62(5):779–786. DOI: 10.2169/internalmedicine.0825-22.
13. Shpilyukova YA, Smetanina OV, Kolpina AA, Illarioshkin SN. A case of chorea with cognitive impairment associated with polycythemia vera. Neurology, Neuropsychiatry, Psychosomatics. 2023;15(6):101–108. DOI: 10.14412/2074-2711-2023-6-101-108.
14. **Hong CM, Yang S-Y, Cho M, Baek DW, Ryu H-S. Reversible striatal hypermetabolism of acquired chorea associated with polycythemia vera. Acta Neurol Belg. 2023;123(5):1997–2000. DOI: 10.1007/s13760-022-02225-z.**
15. Wen H, Jin D, Chen Y, Cui B, Xiao T. Cerebellar venous thrombosis mimicking a cerebellar tumor due to polycythemia vera: a case report. BMC Neurol. 2021;21:225. DOI: 10.1186/s12883-021-02261-1.
16. **Raza HK, Chansysouphanthong T, Singh S, Amir A, Raza MW, Zhang Z, et al. Polycythemia vera complicated by chorea: A case report and the review of Chinese and international literature. Rev Neurol. 2021;177(8):1025–1030. DOI: 10.1016/j.neurol.2020.11.013.**
17. Teive HAG, Germiniani FMB, Munhoz RP, Camargo CHF. Blepharospasm and periorbital edema after imatinib mesylate: improvement with botulinum toxin. Arq Neuropsiquiatr. 2020;78(1):58–59. DOI: 10.1590/0004-282X2019010.9.
18. Olivier PA, Salamon N, Casselman J, van Droogenbroeck J, Vanopdenbosch LJ. Cerebellar progressive multifocal leukoencephalopathy in a patient with a history of Crohn’s disease and acute myeloid leukemia. Acta Neurol Belg. 2020;120(3):759–760. DOI: 10.1007/s13760-019-01260-x.
19. Obeidat K, Alsaud A, Ashour A, Azrieh B, Abu-Tineh M, Mohamed SF, et al. Imatinib-induced tremor in a patient with chronic myeloid leukemia in chronic phase. Case Rep Oncol. 2019;12(3):913–917. DOI: 10.1159/000504937.
20. Betté S, Moore H. Generalized chorea and JAK2V617F mutation–positive myeloproliferative disorders. Mov Disord Clin Pract. 2020;7(4):462–463. DOI: 10.1002/mdc3.12935.
21. Wang X, Billick M, Monsour D, Liu J. Central nervous system graft-versus-host disease in a 68-year-old man presenting with myoclonus. CMAJ. 2019;191(39):E1078–E1081. DOI: 10.1503/cmaj.190216.
22. Safin SM, Bakirov BA, Derevyanko KP, Baǐkov DE, Khafizov MM. A clinical case of cerebellar ataxia with neuropathy and vestibular areflexia syndrome in the presence of polycythemia vera. Neurology, Neuropsychiatry, Psychosomatics. 2019;11(3):74–77. DOI: 10.14412/2074-2711-2019-3-74-77.
23. Rhee JY, Tremblay D, Chan AM, Tallman MS, Mascarenhas J. Myoclonic jerks complicating treatment of acute promyelocytic leukemia: Case report and literature review. Blood Adv. 2019;3(12):1854–1857. DOI: 10.1182/bloodadvances.2019000249.
24. Chan J, Shah P, Moguel-Cobos G. Nilotinib-Induced Dystonia and Cognitive Deficits in a Neurologically Normal Patient with Chronic Myeloid Leukemia. Case Rep Neurol Med. 2019;2019:3679319. DOI: 10.1155/2019/3679319.
25. Bhattacharjee S. Irreversible chorea as the initial presentation of polycythemia rubra vera in an elderly woman. Neurosciences (Riyadh). 2019;24(1):61–62. DOI: 10.17712/nsj.2019.1.20180301.
26. Varghese DR, Joseph D, Prabhu R, Anila KN. High-dose cytarabine–associated cerebellar toxicity: A case report. Natl J Physiol Pharm Pharmacol. 2018;8(1):142–143. DOI: 10.5455/njppp.2018.8.0724818072017.
27. García-Cabo C, Fernández-Domínguez J, Mateos V. Sudden hemichorea and frontal lobe syndrome: A rare presentation of unbalanced polycythaemia vera. BMJ Case Rep. 2018;2018:bcr2017223867. DOI: 10.1136/bcr-2017-223867.
28. Barow E, Hoppe J, Mainka T, Schneider SA, Ganos C. Hematologic follow-up as clue to polycythemia vera due to JAK2 mutation presenting as late-onset chorea. Mov Disord Clin Pract. 2018;5(1):83–85. DOI: 10.1002/mdc3.12548.
29. Kmezic I, Weinberg J, Hauzenberger D, Hashim F, Kollia E, Klimkowska M, et al. An unusual cause of fatal rapid-onset ataxia plus syndrome. Cerebellum Ataxias. 2017;4:5. DOI: 10.1186/s40673-017-0063-9.
30. Enriquez-Marulanda A, Sierra-Ruiz M, Jaramillo FJ, Escobar LA, Granados AM, Rodríguez-Rojas LX, et al. Hemichoreo-hemiballism as a manifestation of central nervous system chronic graft-versus-host disease. Mov Disord Clin Pract. 2017;4(4):495–498. DOI: 10.1002/mdc3.12497,
31. Degnan AJ, Capek E, Bowman A. Chorea in the older adult: A full blooded answer. J R Coll Physicians Edinb. 2016;46(4):244–246. DOI: 10.4997/JRCPE.2016.407.
32. Ssentongo A, Ssentongo P, Sather MD, Claxton DF, Gilliam FG. Intractable myoclonic seizures in an allogeneic stem cell transplant recipient: A rare case of myoclonic epilepsy. Epilepsy Behav Case Rep. 2015;4:48–51. DOI: 10.1016/j.ebcr.2015.06.005.
33. Venkatesan EP, Ramadoss K, Ramasamy R, Prakash B. Essential thrombocythemia: Rare cause of chorea. Ann Indian Acad Neurol. 2014;17(1):106–107. DOI: 10.4103/0972-2327.128569.
34. Miyagawa T, Ueda K, Ohtomo R, Sasaki T, Takahashi M, Momose T, et al. Treatable chorea associated with polycythemia vera. Neurol Clin Neurosci. 2014;2(3):90–91. DOI: 10.1111/ncn3.94.
35. Liu GD, Chang J, Liu ZJ, Qiang Q, Gu CH, Zhang YY, et al. Chorea disclosing a polycythemia vera. Neuropsychiatr Dis Treat. 2014;10:563–565. DOI: 10.2147/NDT.S60694.
36. Kim SW, Yu HM, Lee YJ, Kim HJ, Hur M, Lee HG. Polycythemia vera presenting with generalized chorea: a case report and review of the literature. Korean J Med. 2014;87(5):619–624. DOI: 10.3904/kjm.2014.87.5.619.
37. Adam R, Priglinger M, Harrington T, Gottlieb D, Krause M. An unusual cause of cerebellar hemorrhage in a young patient: Essential thrombocythemia. J Stroke Cerebrovasc Dis. 2014;23(5):e373–e374. DOI: 10.1016/j.jstrokecerebrovasdis.2013.12.013.
38. Lew J, Frucht SJ, Kremyanskaya M, Hoffman R, Mascarenhas J. Hemichorea in a patient with JAK2V617F blood cells. Blood. 2013;121(7):1239–1240. DOI: 10.1182/blood-2012-11-467795.
39. Bhargava R, Dass J, Singh S, Vaid A. Hemichorea, a rare presenting feature of polycythemia vera. Neurol India. 2013;61(1):86–87. DOI: 10.4103/0028-3886.108027.
40. Severs M, Boelens HM, Diraoui SB, Schuur J. Chorea and a frontal lobe syndrome: A rare neurological presentation of polycythemia vera. J Am Geriatr Soc. 2012;60(3):589–590. DOI: 10.1111/j.1532-5415.2011.03850.x.
41. Huang H-C, Wu Y-C, Shih L-Y, Lo W-C, Tsai C-H, Shyu W-C. Reversible abnormal functional neuroimaging presentations in polycythemia vera with chorea. J Neurol. 2011;258(11):2054–2057. DOI: 10.1007/s00415-011-6069-y.
42. Ben Ghorbel I, Ben Salem T, Lamloum M, Khanfir M, Braham A, Miled M, et al. Chorea revealing a polycythemia vera. Rev Med Interne. 2011;32(6):e79–e80. DOI: 10.1016/j.revmed.2010.06.015.
43. Kumar H, Masiowski P, Jog M. Chorea in the elderly with mutation positive polycythemia vera: A case report. Can J Neurol Sci. 2009;36(3):370–372. DOI: 10.1017/S0317167100007149.
44. Pratesi A, Vella A, Pasini E, Salvi F, Mascalchi M. Parkinsonism in polycythaemia vera probably due to manganism. Mov Disord. 2008;23(16):2420–2421. DOI: 10.1002/mds.22302.
45. Lee JE, Shin H-W, Sohn YH. Chorea as an Initial Manifestation of Polycythemia Vera. J Mov Disord. 2008;1(2):82–85. DOI: 10.14802/jmd.08020.
46. Kim W, Kim JS, Lee KS, Kim YI, Park CW, Chung YA. No evidence of perfusion abnormalities in the basal ganglia of a patient with generalized chorea-ballism and polycythaemia vera: Analysis using subtraction SPECT co-registered to MRI. Neurol Sci. 2008;29(5):351–354. DOI: 10.1007/s10072-008-0994-2.
47. Dubow JS, Panush SR, Rezak M, Leikin J. Acute dystonic reaction associated with foscarnet administration. Am J Ther. 2008;15(2):184–186. DOI: 10.1097/MJT.0b013e31802f5e7d.
48. Midi I, Dib H, Köseoglu M, Afşar N, Günal DI. Hemichorea associated with polycythaemia vera. Neurol Sci. 2006;27(6):439–441. DOI: 10.1007/s10072-006-0727-3 .
49. Golla H, Thier P. Ocular flutter—A sign of brain-stem pathology as rare consequence of cyclosporin A treatment. Neuro-Ophthalmology. 2005;29(2):81–84. DOI: 10.1080/01658100590933406.
50. Bishton MJ, Das Gupta E, Byrne JL, Russell NH. Opsoclonus myoclonus following allogeneic haematopoietic stem cell transplantation. Bone Marrow Transplant. 2005;36:923. DOI: 10.1038/sj.bmt.1705153.
51. Tan EK, Chan LL, Lo YL. “Myorhythmia” slow facial tremor from chronic interferon alpha-2a usage. Neurology. 2003;61(9):1302–1303. DOI: 10.1212/01.WNL.0000094310.62850.0C.
52. Sarasombath P, Sumida KN, Kaku DA. Parkinsonism associated with interferon alpha therapy for chronic myelogenous leukemia. Hawaii Med J. 2002;61:48–57.
53. Friedman JH, Shetty N. Permanent cerebellar toxicity of cytosine arabinoside (Ara-C) in a young woman. *Movement Disorders*. 2001;16(3):575–577. DOI: 10.1002/mds.1098 ,
54. Nazabal ER, López JM, Pérez PA, del Corral PR. *Chorea disclosing deterioration of polycythaemia vera.* Postgrad Med J. 2000;76(900):658-9. doi: 10.1136/pmj.76.900.658.
55. Apsner R, Schulenburg A, Steinhoff N, Keil F, Janata K, Kalhs P, et al. *Cyclosporin A-induced ocular flutter after marrow transplantation.* Bone Marrow Transplant. 1997;20(3):255-256. doi: 10.1038/sj.bmt.1700809.
56. Mott SH, Packer RJ, Vezina LG, Kapur S, Dinndorf PA, Conry JA, et al. *Encephalopathy with parkinsonian features in children following bone marrow transplantations and high-dose amphotericin B.* Ann Neurol. 1995;37(6):810-814. doi: 10.1002/ana.410370616.
57. Leferink J, Ferbert A, Hirschmann WD. *Severe cerebellar syndrome caused by cytosine arabinoside (Alexan®) in therapy of leukaemia.* Aktuelle Neurologie. 1995;22(5):186–188.
58. Krstenansky PM, Petree J, Long G. *Extrapyramidal reaction caused by ondansetron.* Ann Pharmacother. 1994;28(2):280. doi: 10.1177/106002809402800223.
59. Balmaceda CM, Walker RW, Castro-Malaspina H, Dalmau J. *Reversal of amphotericin-B-related encephalopathy.* Neurology. 1994;44(6):1183–1184. doi: 10.1212/WNL.44.6.1183.
60. Cohen AM, Gelvan A, Yarmolovsky A, Djaldetti M. *Chorea in polycythemia vera: a rare presentation of hyperviscosity.* Blut. 1989;58(1):47–48.
61. Luque FA, Selhorst JB, Petruska P. *Parkinsonism induced by high-dose cytosine arabinoside.* Mov Disord. 1987;2(3):219-222. doi: 10.1002/mds.870020309.
62. Cersosimo RJ, Carter RT, Matthews SJ, Coderre M, Karp DD. *Acute cerebellar syndrome, conjunctivitis, and hearing loss associated with low-dose cytarabine administration.* Drug Intell Clin Pharm. 1987;21(10):798-803. doi: 10.1177/106002808702101007.
63. Vilter RW. *Vidarabine-associated encephalopathy and myoclonus.* Antimicrob Agents Chemother. 1986;29(5):933-935. doi: 10.1128/aac.29.5.933.
64. Cold S. *Cerebellar dysfunction during high-dose cytosine arabinoside therapy in a case of acute myelogenous leukaemia.* Scand J Haematol. 1986;36(2):165–167.
65. Mas JL, Guéguen B, Bouche P, Dérouesné C, Varet B, Castaigne P. *Chorea and polycythaemia.* J Neurol. 1985;232(3):169–171. doi: 10.1007/BF00313895.
66. Dworkin LA, Goldman RD, Zivin LS, Fuchs PC. *Cerebellar toxicity following high-dose cytosine arabinoside.* J Clin Oncol. 1985;3(5):613-616. doi: 10.1200/JCO.1985.3.5.613.
67. Coppack SW, Gibberd FB. *Chorea with transitional myeloproliferative disease.* J R Soc Med. 1985;78(10):864–866. doi: 10.1177/014107688507801017.
68. Saper CB, Jarowski CI. *Leukemic infiltration of the cerebellum in acute myelomonocytic leukemia.* Neurology. 1982;32(1):77–80. doi: 10.1212/WNL.32.1.77.
69. Morré HHE, van Woerkom TCAM, Endtz LJ. A case of chorea due to polycythaemia vera. *Clinical Neurology and Neurosurgery*. 1982;84(2):125–130.
70. Borg-Costanzi JM, Mohr PD, Lewis D. Primary polycythaemia presenting with chorea. *Postgraduate Medical Journal*. 1981;57(666):228–231.
71. Voiculescu V, Ionescu DA, Lazar G, Alecu C, Predescu C, Popa C, et al. Two cases of choreatic syndrome caused by polycythemia vera. *European Neurology*. 1979;18(2):96–100. doi:10.1159/000115061
72. Edwards PD, Prosser R, Wells CEC. Chorea, polycythaemia, and cyanotic heart disease. *Journal of Neurology, Neurosurgery, and Psychiatry*. 1975;38(8):729–739. doi:10.1136/jnnp.38.8.729.
73. Ashenhurst EM. Chorea complicating polycythemia rubra vera. *Canadian Medical Association Journal*. 1972;107(5):434–437.
74. Friedemann M, Mumenthaler M, Kummer H. *Chorea bei Polycythaemia vera.* Zeitschrift für Neurologie. 1965;187:585–594.
