# Supplementary material for: Spectrum of Movement Disorders in Hematological Malignancies: A Comprehensive Systematic Review of Clinical Phenotypes, Mechanisms, and Outcomes
Source: Tremor Other Hyperkinet Mov (N Y). 2026 Mar 19;16:18. doi: 10.5334/tohm.1147 (PMC13004057; doi:10.5334/tohm.1147)
Supplement: Supplementary File. — Supplementary Tables 1 to 4. [file tohm-16-1-1147-s1.zip › tohm-1147_garg-s1/Supplementary Table 3.docx]

**Supplementary Table 3: Systematic Case Compilation of Movement Disorders, CNS Involvement, and Immunologic/Drug-Related Mechanisms in Plasma Cell Neoplasms (n = 22)**

| **Author/Year** | **Country** | **Age/Sex** | **Hematological Category** | **Specific Diagnosis** | **Disease Stage (ISS/R-ISS)** | **Molecular Markers / Cytogenetics** | **CNS Involvement (Evidence)** | **Movement Disorder / Ataxia Type** | **Phenomenology** | **Timing of Onset** | **Associated Neurologic Manifestations** | **Evidence Supporting Mechanism** | **Paraneoplastic Antibodies** | **Drug-Induced Neurotoxicity** | **CSF Findings** | **Neuroimaging Findings** | **PET-CT Findings** | **Hematology Treatment** | **Targeted / Novel Therapy** | **HSCT** | **Neurology Treatment** | **Neurological Outcome** | **Hematological Outcome** | **Proposed Mechanism** | **Follow-up Duration** |
| --- | --- | --- | --- | --- | --- | --- | --- | --- | --- | --- | --- | --- | --- | --- | --- | --- | --- | --- | --- | --- | --- | --- | --- | --- | --- |
| Wu AS et al., 2025 | Canada | 77/M | Plasma-cell neoplasm | IgA-λ Multiple Myeloma (R-ISS 3) | R-ISS 3 | Not reported | No structural lesions; CSF negative; CAR-T neurotoxicity | Parkinsonism | Rigidity, bradykinesia, tremor, stooped posture, shuffling gait, hypophonia, micrographia | 19 days post CAR-T infusion | Bilateral facial palsy; perioral paresthesia | CAR‑T neurotoxicity; immune‑mediated; pregabalin susceptibility | Not reported | Possible pregabalin contribution | 12 WBC/µL, protein 0.58 g/L, glucose 5 mmol/L; cytology negative | Normal MRI except microangiopathic changes | Not reported | Bortezomib + lenalidomide + dexamethasone; BCMA CAR‑T (cilta‑cel) | Ciltacabtagene autoleucel | Not reported | Dexamethasone taper; stop pregabalin | Complete resolution | Not stated | CAR‑T immune neurotoxicity | At least 46 days post infusion |
| Wirk B & Lim J, 2025 | USA | 53/F | Plasma-cell neoplasm | Multiple myeloma (R-ISS I), κ light chain, +1q, del17p13, DNMT3A mutation | R-ISS I | 46XX, +1q, del17p13, DNMT3A mutation | MRI basal ganglia T1 hyperintensity; FDG-PET caudate/frontal hypometabolism; DAT normal | Parkinsonism | Hypomimia, hypophonia, rigidity, bradykinesia, micrographia, shuffling gait | Day 30 post CAR-T | Stage 4 parkinsonism; no ICANS | CAR-T expansion + IEC-HS inflammation | Negative | None | Normal CSF; negative infectious/autoimmune/paraneoplastic | MRI T1 hyperintensity; PET hypometabolism | Whole-body PET negative | Fludarabine + cyclophosphamide; Cilta-cel | Ruxolitinib | No | IVIG, steroids, anakinra, ruxolitinib | Fully resolved | Stringent CR; MRD-negative | IEC-HS cytokine hyperinflammation | 1 year |
|  |  | 70/M | Plasma-cell neoplasm | IgA-κ MM; R-ISS I; del17p13, del1p36, del14q32 | R-ISS I | 46XY, del17p13, del1p36, del14q32 | FDG-PET frontal hypometabolism; MRI normal; DAT normal | Parkinsonism | Bradykinesia, rigidity, shuffling gait, hypomimia, micrographia | Day 17 post CAR-T | Stage 4 parkinsonism; no ICANS | IEC-HS hyperinflammation | Negative | None | Normal CSF; negative panels | MRI normal; PET hypometabolism | Whole-body PET MRD-negative | Fludarabine + cyclophosphamide; Cilta-cel | Ruxolitinib | No | IVIG, steroids, anakinra, ruxolitinib | Complete recovery; independent ADLs | Stringent CR; MRD-negative | IEC-HS cytokine-driven parkinsonism | 6 months |
| Cheng Z et al., 2025 | China | 60/F | Plasma-cell neoplasm | IgG-λ multiple myeloma (ISS II) | ISS II | IGH/FGFR3 fusion (40%) | GluK2 antibody positivity; weak TBA positivity; CSF later negative | Involuntary movements | Lower-limb jerks → face; eye squeezing, lip smacking, chewing, slurred speech, brief unresponsiveness; hysterical shouting | 2-year duration, worsening last 2 months | Mild cognitive impairment; bilateral Babinski & Chaddock signs | GluK2 antibodies affecting basal ganglia/cortical circuits | GluK2 antibody positive (1:100 → 1:10) | None | Initial CSF refused; later CSF without plasma cells; GluK2 negative | MRI: small vessel disease; no AE lesions | ECT: pulmonary embolism; no PET CNS findings | VCD regimen (Bortezomib, Cyclophosphamide, Dexamethasone) | None | No | Supportive care; AE workup | Full neurological recovery | Improved MM parameters; decreased antibody titers | Paraneoplastic GluK2-mediated autoimmune encephalitis | Several months follow-up |
| Schneider S et al., 2024 | USA | 54/F | Plasma-cell neoplasm | Relapsed/refractory multiple myeloma, Stage III | Not explicitly stated | Not reported | Normal MRI; DAT-SPECT normal; ICANS present | Hypokinetic parkinsonism | Hypomimia, hypophonia, bradykinesia, rigidity, shuffling gait, micrographia, cognitive slowing | 3 weeks post CAR-T | Cognitive dysfunction, expressive aphasia during ICANS | Cytokine-mediated basal ganglia dysfunction; CAR-T-related MNT | Not reported | Levodopa and trihexyphenidyl ineffective | Not reported | MRI normal basal ganglia & midbrain | Not reported | Cilta-cel; multiple prior regimens; ASCT | None | Yes, prior ASCT | Steroids, IVIG, levodopa, trihexyphenidyl, amantadine | Persistent but improved by 17 months | MM controlled | CAR-T–associated MNT; cytokine/inflammatory mechanism | 17 months |
| Kandemir M & Atalay F, 2024 | Turkey | 67/M | Plasma-cell neoplasm | Multiple myeloma (2019), post-HSCT; on lenalidomide | Not reported | Not reported | No CNS involvement; tremor due to drug + hypoxia + anemia | High-frequency postural tremor | Bilateral high-frequency tremor (R>L), balance issues, dizziness | 72 hours after TMP-SMX began | Balance difficulty; exertional dyspnea; dizziness | TMP-SMX toxicity + hypoxia + anemia + familial predisposition | Not applicable | TMP-SMX neurotoxicity | Not performed | Not performed | Not performed | CyBorD; HSCT (2022); lenalidomide maintenance | None | Yes | Dose reduction of TMP-SMX; RBC transfusion | Near-complete resolution | Hb improved from 8.2 → 11.5 g/dL | Drug toxicity potentiated by hypoxia + anemia | 3 weeks |
| Aliakbar R et al., 2024 | USA | 67/M | Plasma-cell neoplasm | Multiple myeloma, R-ISS II, extramedullary disease | R-ISS II | 1q gain; oligo-secretory EMD | CSF 71 WBC (CD3+); PET deep nuclei hypermetabolism | Parkinsonism (MNT) | Apathy, masked facies, bradykinesia, rigidity, resting tremor, falls | Day +19 after CAR-T | Cognitive slowing, inattention, apathy | CAR-T–mediated presynaptic dopaminergic injury; BCMA on-target CNS effect | Negative panels | None (no dopamine blockers) | 71 WBC, all T cells; AE panel negative; later CSF acellular | FDG-PET: diffuse caudate/putamen/thalami hypermetabolism | Whole-body PET: stringent CR | Fludarabine/cyclophosphamide → Cilta-cel | Cilta-cel | Yes (prior autologous HSCT) | IVIG, steroids, cyclophosphamide, levodopa, ropinirole, amantadine | Near-resolution on dopaminergic regimen | Stringent CR | Presynaptic dopaminergic terminal dysfunction | Until Day +94 |
| Kürtüncü M & Tüzün E, 2021 | Turkey | 65/M | Plasma-cell neoplasm | Multiple myeloma (kappa light chain) | Not reported | Kappa light-chain monoclonal protein | No CNS infiltration; PCD based on MRI + CSF OCB | Cerebellar ataxia | Truncal ataxia, mild appendicular ataxia, tremor, dysarthria, memory loss | 3 months | Short-term memory loss, dysarthria | Paraneoplastic: MRI cerebellar atrophy, OCB, negative antibodies | Negative full neuronal antibody panel | None | CSF: 3 cells/mm3; Protein 45 mg/dL; Glucose 58 mg/dL; IgG index 0.47; OCB pattern 5; no malignant cells | MRI: cerebellar atrophy; WM hyperintensities; no enhancement | Whole-body PET normal | Bortezomib + cyclophosphamide + steroids | None | No | High-dose methylprednisolone | Progressive deterioration | MM confirmed; treated | Paraneoplastic cerebellar degeneration | 6 months (until death) |
| Yanagihara W et al., 2020 | Japan | 33/F | Plasma-cell neoplasm (extramedullary plasmacytoma) | Primary cerebellar plasmacytoma | Not applicable | CD38+, κ-restriction, Ki-67 6.2% | Direct cerebellar tumor; no MM | Cerebellar ataxia | Vertigo, left ataxia, resolved facial palsy | 6 days postpartum → progression over 2 months | Previous transient facial palsy | Mass effect + plasma cell tumor infiltration | Not applicable | None | Not reported | MRI: T1 low, FLAIR high, strong enhancement, high DWI & ASL | Not performed | Surgical resection + 50 Gy radiotherapy | Bortezomib maintenance | No | Surgical decompression | Full neurological recovery | No MM at 16 months | Local mass effect from plasmacytoma | 16 months |
| Sagar F et al., 2018 | USA | 71/F | Plasma-cell neoplasm | IgA-κ Multiple Myeloma | ISS II | FISH: loss 1p, +7, +9, +11, IgH gain, +1q21 | None; MRI/CT normal | Tardive dyskinesia-like EPS | Lip-smacking, jaw/tongue movements, blinking, frowning, chewing, choreoathetoid limb movements | 3 months after starting lenalidomide | Speech difficulty; AIMS score diagnostic | Temporal association; IMiD-related dopaminergic dysfunction | Not tested | Lenalidomide neurotoxicity | Not done | MRI & CT normal | Not performed | KRd → Auto-HSCT → LEN maintenance | Lenalidomide | Yes (autologous) | Diphenhydramine, diazepam, clonazepam; stopped LEN | Improved; no persistent TD | MM in remission | IMiD dopaminergic dysfunction | Short-term |
| Argente-Escrig H et al., 2018 | Spain | 64/F | Plasma-cell neoplasm | IgG-κ Multiple Myeloma, Stage IIIa | ISS IIIa | Not reported | No CNS MM; MRI/EEG consistent with CJD | Parkinsonism + dystonia | Bradykinesia, rigidity, dystonia, neglect, hallucinations, confusion | 2 weeks after starting lenalidomide | Rapid dementia, hallucinations, gait disturbance | MRI DWI hyperintensity; EEG periodic discharges; LEN-triggered CJD activation | Not reported | Lenalidomide neurotoxicity / CJD unmasking | 14-3-3 negative; CSF infectious & autoimmune negative | DWI cortical ribbon hyperintensity (frontal, parietal, occipital) | Not performed | LEN + Dex (relapse regimen) | Lenalidomide | Yes (autologous HSCT) | Supportive; stop LEN | Relapse later → akinetic mutism → death | MM in remission before decline | LEN-triggered exacerbation of preclinical sCJD | ~4 months |
| Aquino et al., 2018 | Canada | 66/M | Plasma cell dyscrasia | Smoldering multiple myeloma → overt MM | Progressive (with anemia, IgG rise) | IgG-κ monoclonal protein; IgG 360→593 g/L; κ/λ ratio 4.7→6 | Diffuse leukoencephalopathy on MRI | Holmes tremor + ataxia | Rest + postural + action tremor; dysmetria; pyramidal signs; cognitive deficits | Over ~2 years | Ataxia, pyramidal signs, mild cognitive impairment | MRI improvement with myeloma therapy | Negative | None (treatment-naïve before symptoms) | Elevated protein 1.04 g/L; normal cells | T2/FLAIR confluent WM hyperintensity; improved after therapy | Whole-body CT normal | Melphalan + prednisone | None | No | Primidone, levodopa, propranolol, clonazepam, gabapentin, topiramate (no benefit); clozapine partial benefit; thalamotomy; DBS | Stabilized; tremor improved | Good hematologic response | Myeloma-associated immune-mediated leukoencephalopathy | ≥1 year follow-up; DBS at 2.5 years |
| Zis et al., 2017 | United Kingdom (Sheffield) | 65/F | Plasma cell dyscrasia | Light-chain multiple myeloma (lambda) | Not reported; systemic involvement | Lambda light chains | Paraneoplastic cerebellar dysfunction + sensory ganglionopathy | Cerebellar ataxia + sensory ganglionopathy | Gaze-evoked nystagmus, gait/heel-shin ataxia, slurred speech, sensory loss | Unsteadiness x6 yrs; slurred speech x2 yrs | Sensory ganglionopathy | Paraneoplastic (no other cause identified) | None reported | None | Not reported | Progressive cerebellar atrophy; abnormal MRS | Not reported | Referred; regimen not described | Not reported | Not reported | Supportive care only | Progressive worsening | New myeloma diagnosis (poor prognosis) | Paraneoplastic mechanism | 54 months |
| Guner et al., 2015 | Turkey | 65/M | Plasma cell dyscrasia | Multiple myeloma | Not reported | Not reported | No CNS myeloma; drug‑induced toxicity | Multifocal myoclonus | Shock‑like jerks impairing daily activity | 7 days after high‑dose melphalan + ASCT | Sensory neuropathy (diabetic + bortezomib) | Gabapentin toxicity (high serum level) | None | Gabapentin toxicity | Not reported | Not reported | Not reported | Bortezomib → melphalan → ASCT | Bortezomib | Autologous HSCT | Gabapentin stopped | Complete resolution | Not reported | Drug toxicity | Not reported |
| Aricò et al., 2013 | Italy | 78/F | Plasma cell dyscrasia | Multiple myeloma | Not reported | Monoclonal plasma cells 25–30% | No CNS involvement | Restless legs syndrome (RLS) | Unpleasant leg sensations, urge to move, severe PLMS | 8 months before presentation | Lumbar-sacral radiculopathy; pruritus | Iron deficiency + anemia causing RLS | None | None | Not reported | Brain CT normal; spine CT L3–L4 disc protrusion | Not reported | Iron replacement; MM referred to hematology | None | None | Gabapentin + pramipexole | Major improvement; PSG normalized | MM newly diagnosed; no response data | Secondary RLS due to iron deficiency | 1 month |
| Fickweiler et al., 2009 | Netherlands | 61/F | Plasma cell dyscrasia | Multiple myeloma | Not reported | Not reported | Cerebellar cryptococcoma; CSF positive | Cerebellar syndrome | Nausea, vertigo, downbeat nystagmus, dysmetria, tremor | 3 weeks | Hydrocephalus; mass effect | Severe CD4 lymphopenia → opportunistic infection | None | None | India ink + CSF culture positive | Cerebellar mass with enhancement + hydrocephalus | Not reported | Multiple prior chemo regimens incl. melphalan + auto-SCT | Thalidomide | Autologous HSCT | Third ventriculostomy | Near-complete regression on MRI | Ongoing MM (no remission data) | Opportunistic fungal infection (CD4 loss) | Not reported |
| Clow et al., 2008 | Canada | 31/F | Plasma cell dyscrasia | IgG-κ multiple myeloma + sacral plasmacytoma | Progressive → autologous BMT → long remission | IgG-κ monoclonal protein | No CNS infiltration; autoimmune SPS | Stiff-person syndrome | Axial & proximal stiffness; spasms; hyperreflexia; myoclonus; stiff gait; EMG continuous activity | 1–2 years after autologous BMT + interferon | Myoclonic jerks, gait impairment, stiffness | Anti‑GAD65 positive; EMG findings; response to IVIG; post‑transplant immune reconstitution | Anti‑GAD65 strongly positive; others negative | None reported | Not examined | CT head normal; spine MRI normal except scoliosis | Not done | Radiotherapy, TBI 1200 cGy, melphalan 140 mg/m2, interferon maintenance | Interferon‑α | Autologous HSCT | IVIG, steroids, baclofen, diazepam | Improved but persistent SPS | Excellent remission >10 years | Aberrant immune reconstitution + interferon; autoimmune anti‑GAD SPS | >10 years |
| Chiruka & Chapman, 2005 | United Kingdom | 66/F | Plasma cell dyscrasia | Multiple myeloma | Relapsed | Not reported | None | Severe tremors | Incapacitating tremors of limbs | 2 months / 2 weeks on rechallenge | None | Temporal association; reproducible | Not tested | Thalidomide neurotoxicity | Not reported | Not reported | Not reported | VAD prior; thalidomide | None | No | Stopping thalidomide | Reversible | Relapsed MM | Thalidomide neurotoxicity | Not stated |
|  |  | 65/M | Plasma cell dyscrasia | Multiple myeloma | Progressive | Not reported | None | Tremors | Debilitating rest/action tremors | 1 month | None | Temporal relation | Not tested | Thalidomide neurotoxicity | Not reported | Not reported | Not reported | Multiple regimens; thalidomide+cyclophosphamide | None | Autologous SCT | None specific | Chronic tremors | Progressive MM | Thalidomide neurotoxicity | Not stated |
| Fleming & Mangino, 1997 | USA | 54/M | Plasma cell dyscrasia | Multiple myeloma | Refractory | Not reported | No CNS infiltration; normal CT & CSF | Parkinsonian syndrome | Bradykinesia, resting tremor, cogwheel rigidity, limited voluntary motion | Within days after high‑dose chemotherapy | Encephalopathy; noncommunicative state | Temporal relationship; renal failure altering drug clearance | Not tested | Cyclophosphamide/etoposide neurotoxicity | Normal | Normal CT | Not reported | High‑dose cyclophosphamide + etoposide + other chemo | None | None | Sinemet | Improved; regained meaningful conversation | Not detailed | Drug-induced parkinsonism (high-dose chemo + renal failure) | Short inpatient follow‑up |
| Fahn et al., 1996 | USA | 45/M | Plasma cell dyscrasia | IgM monoclonal gammopathy; later lytic lesions (MM) | Progressive neurodegeneration | IgM monoclonal gammopathy | No CNS infiltration; neurodegenerative | Rapidly progressive parkinsonism | Akinetic‑rigid, poor levodopa response, moaning, autonomic dysfunction | Onset at 45 years | Autonomic neuropathy, pyramidal signs, bulbar dysfunction, confusion | Clinical picture consistent with MSA | Not tested | Levodopa‑induced moaning | Not reported | Not detailed (MSA suspected) | Not reported | Supportive; myeloma detected later | None | None | Levodopa (poor response) | Progressive decline | Progression to MM with lytic lesions | Probable MSA (striatonigral degeneration) | Several years |
| Akpinar et al., 1990 | Turkey | 57/F | Plasma cell dyscrasia | Multiple myeloma (IgG-κ) | Initially occult, later progressive | IgG monoclonal gammopathy; κ Bence-Jones | Paraneoplastic cerebellar degeneration | Generalized cerebellar ataxia | Gait, limb & truncal ataxia; dysarthria; titubation; tremor; myoclonus | Subacute over 3 months | Late sensory–motor polyneuropathy | PCT + MRI findings + gammopathy evolution | Not tested | None | Normal CSF except mild ↑γ-globulin | CT normal then cerebellar atrophy; MRI vermian atrophy | Not done | Vincristine, melphalan, cyclophosphamide, prednisone | None | No | Amitriptyline, piracetam, propranolol, prednisolone | Progressive irreversible cerebellar syndrome | Progressive myeloma with IgG rise, proteinuria, anemia | Autoimmune paraneoplastic cerebellar degeneration | 3.4 years |
|  |  |  |  |  |  |  |  |  |  |  |  |  |  |  |  |  |  |  |  |  |  |  |  |  |  |

**AIMS – Abnormal Involuntary Movement Scale; AL – Amyloid Light-chain; AL (κ) – Kappa-type Amyloid Light Chain; Anti-CASPR2 – Anti–Contactin Associated Protein-2 Antibody; Anti-GAD65 – Anti–Glutamic Acid Decarboxylase-65 Antibody; Anti-LGI1 – Anti–Leucine-Rich Glioma Inactivated-1 Antibody; ASCT – Autologous Hematopoietic Stem Cell Transplantation; ASL – Arterial Spin Labeling; BCMA – B-Cell Maturation Antigen; BMT – Bone Marrow Transplant; CAR-T – Chimeric Antigen Receptor T-cell Therapy; CD – Cluster of Differentiation; CDC – Centers for Disease Control and Prevention; CJD – Creutzfeldt–Jakob Disease; CNS – Central Nervous System; CR – Complete Response; CSF – Cerebrospinal Fluid; CT – Computed Tomography; CyBorD – Cyclophosphamide + Bortezomib + Dexamethasone; DAT-SPECT – Dopamine Transporter Single-Photon Emission Computed Tomography; Dex – Dexamethasone; DWI – Diffusion-Weighted Imaging; EMD – Extramedullary Disease; EMG – Electromyography; FDG-PET – Fluorodeoxyglucose Positron Emission Tomography; FGFR3 – Fibroblast Growth Factor Receptor-3; FLAIR – Fluid-Attenuated Inversion Recovery; FLC – Free Light Chains; Hb – Hemoglobin; HIV – Human Immunodeficiency Virus; HSCT – Hematopoietic Stem Cell Transplant; ICANS – Immune Effector Cell–Associated Neurotoxicity Syndrome; IEC-HS – Immune Effector Cell–Associated Hemophagocytic Syndrome; IgA – Immunoglobulin A; IgG – Immunoglobulin G; IgM – Immunoglobulin M; IMiD – Immunomodulatory Drug; ISS – International Staging System; K/L ratio – Kappa/Lambda Ratio; LEN – Lenalidomide; MGUS – Monoclonal Gammopathy of Undetermined Significance; MNT – Movement Disorder Neurotoxicity; MM – Multiple Myeloma; MoCA – Montreal Cognitive Assessment; MRI – Magnetic Resonance Imaging; MRD – Minimal Residual Disease; MRS – Magnetic Resonance Spectroscopy; MSA – Multiple System Atrophy; OCB – Oligoclonal Bands; PCD – Paraneoplastic Cerebellar Degeneration; PCR – Polymerase Chain Reaction; PET-CT – Positron Emission Tomography–Computed Tomography; PLMS – Periodic Limb Movements of Sleep; PSG – Polysomnography; R-ISS – Revised International Staging System; RLS – Restless Legs Syndrome; RT – Radiotherapy; sCJD – Sporadic Creutzfeldt–Jakob Disease; SCT – Stem Cell Transplant; SPS – Stiff-Person Syndrome; TBA – Tissue-Based Assay; TBI – Total Body Irradiation; TMP-SMX – Trimethoprim–Sulfamethoxazole; T2/FLAIR – T2-weighted/Fluid-Attenuated Inversion Recovery; VAD – Vincristine + Adriamycin + Dexamethasone; VCD – Bortezomib + Cyclophosphamide + Dexamethasone; VIM DBS – Ventral Intermediate Nucleus Deep Brain Stimulation; WBC – White Blood Cell; WM – White Matter.**

**References**

1. Wu AS, Hophing L, Gosse P, Motamed M, Bhella SD, Stewart K, et al. Parkinsonism and bilateral facial palsy after chimeric antigen receptor T-cell therapy. *Movement Disorders Clinical Practice.* 2025;12(3):371–374. doi:10.1002/mdc3.14335.
2. Wirk B, Lim J. Ruxolitinib is an effective therapy for ciltacabtagene autoleucel-associated parkinsonism in multiple myeloma. *Journal of Hematology.* 2025;14(3):146–151. doi:10.14740/jh2046.
3. Cheng Z, Song Y, Zhao S, Sui X, Xie L, Zhao H, et al. Anti-GluK2 antibody-positive autoimmune encephalitis concurrent with multiple myeloma: a case report. *BMC Neurology.* 2025;25(1):27. doi:10.1186/s12883-025-04037-3.
4. Schneider S, Aamodt WW, Pruitt AA, Berger JR. Parkinsonism associated with anti–B-cell maturation antigen chimeric antigen receptor T-cell therapy. *Movement Disorders Clinical Practice.* 2024;11(12):1625–1628. doi:10.1002/mdc3.14239.
5. Kandemir M, Atalay F. Contributing factors in trimethoprim–sulfamethoxazole–induced tremor in *Pneumocystis jirovecii* pneumonia. *Turk Noroloji Dergisi (Turkish Journal of Neurology).* 2024;30(3):193–194. doi:10.55697/tnd.2024.111.
6. Aliakbar R, Manouvakhova O, Wong C, Htut M, Pulst-Korenberg J, Janakiram M, et al. Treatment of parkinsonism secondary to ciltacabtagene autoleucel using a combination dopaminergic regimen. *Frontiers in Immunology.* 2024;15:1444010. doi:10.3389/fimmu.2024.1444010.
7. Kürtüncü M, Tüzün E. Paraneoplastic cerebellar degeneration in a patient with multiple myeloma. *Leukemia & Lymphoma.* 2021;62(10):2556–2557. doi:10.1080/10428194.2021.1927022.
8. Yanagihara W, Beppu T, Ogasawara Y, Ito S, Sato Y, Sugai T, et al. Primary plasmacytoma in the cerebellum: A case report and literature review.*World Neurosurgery.* 2020;134:10–13. doi:10.1016/j.wneu.2019.10.143.
9. Sagar F, Malik SU, Soontornprueksa S, Ijaz A, Usman M, Khan AY, et al.Extrapyramidal symptoms with administration of lenalidomide maintenance therapy for multiple myeloma. *Cureus.* 2018;10(9):e3349. doi:10.7759/cureus.3349.
10. Argente-Escrig H, Martinez JC, Gómez E, Balaguer A, Sevilla T, Bataller L.Lenalidomide-induced reversible parkinsonism, dystonia, and dementia in subclinical Creutzfeldt–Jakob disease. *Journal of the Neurological Sciences.* 2018;393:140–141. doi:10.1016/j.jns.2018.08.016.
11. Aquino CC, Connolly B, Lang AE. Smoldering multiple myeloma–associated leukoencephalopathy presenting with Holmes tremor, ataxia, and pyramidal syndrome.*Movement Disorders Clinical Practice.* 2018;5(4):433–435. doi:10.1002/mdc3.12664.
12. Zis P, Rao DG, Wagner BE, Nicholson-Goult L, Hoggard N, Hadjivassiliou M. Cerebellar ataxia and sensory ganglionopathy associated with light-chain myeloma. *Cerebellum & Ataxias.* 2017;4(1):1. doi:10.1186/s40673-016-0060-4.
13. Guner SI, Pamukçuoglu M, Sucak G. A patient with multiple myeloma who developed severe myoclonus after stem cell transplantation. *Journal of Leukemia.* 2015;3(4):193. doi:10.4172/2329-6917.1000193.
14. **Aricò D, Raggi A, Siragusa M, Zucconi M, Ferri R. Restless legs syndrome as the presenting symptom of multiple myeloma.** Journal of Clinical Sleep Medicine. **2013;9(4):383–385. doi:10.5664/jcsm.2596.**
15. Fickweiler W, Ariës MJH, Enting RH, Vellenga E, De Keyser J. Cryptococcal cerebellitis after chemotherapy and autologous stem cell re-infusion in a patient with multiple myeloma. *Journal of Neurology.* 2009;256(1):145–146. doi:10.1007/s00415-009-0127-8.
16. Clow EC, Couban S, Grant IA. Stiff-person syndrome associated with multiple myeloma following autologous bone marrow transplantation. *Muscle & Nerve*. 2008;38(6):1649–1652. doi:10.1002/mus.21153.
17. Chiruka S, Chapman CS. Severe tremors associated with use of thalidomide. *American Journal of Hematology*. 2005;78(1):81–82. doi:10.1002/ajh.20216.
18. Fleming DR, Mangino PB. Parkinsonian syndrome in a dialysis-supported patient receiving high-dose chemotherapy for multiple myeloma. *Southern Medical Journal*. 1997;90(3):364–365.
19. Fahn S, Brin MF, Dwork AJ, Weiner WJ, Goetz CG, Rajput AH. What is it? Case 1, 1996: Rapidly progressive parkinsonism, incontinence, impotency, and levodopa-induced moaning in a patient with multiple myeloma. *Movement Disorders*. 1996;11(3):298–310.
20. Akpinar S, Berk O, Karaca L. Paraneoplastic cerebellar degeneration associated with multiple myeloma: 3.4 years follow-up. *Journal of Neuro-Oncology*. 1990;9(1):63–68.
